# Long-term atorvastatin improves cognitive function by modulating SIRT2-mediated dynamic transition of NFL lysine 272 crotonylation to ubiquitination in naturally aging rats

**DOI:** 10.1038/s41420-025-02764-7

**Published:** 2025-10-16

**Authors:** Tian-Ce Xu, Ji-Ru Cai, Hui-Sheng Chen

**Affiliations:** Department of Neurology, General Hospital of Northern Theater Command, Shenyang, China

**Keywords:** Cognitive ageing, Preclinical research

## Abstract

Proteins’ reversible post-translational modifications (PTMs) are essential for cellular regulation, but their involvement in statin-mediated neuroprotection remains elusive. Our previous research demonstrated that long-term atorvastatin intervention ameliorates cognitive decline in naturally aged rats, although the underlying mechanisms were unknown. Here, we employed a proteomic and PTMomic approach using mass spectrometry-based quantitative proteomics and bioinformatics analyses to elucidate the molecular underpinnings. We also utilized isolated and cultured rat hippocampal neuronal cells to investigate the regulation of neurofilament light chain (NFL) modifications, including crotonylation and ubiquitination, using techniques such as immunoprecipitation, cell transfection, protein imprinting, and PCR. We identified several novel findings: (1) Long-term atorvastatin treatment significantly reduced NFL protein levels in both brain tissue and serum compared to controls; (2) This intervention decreased NFL crotonylation while enhancing ubiquitination via SIRT2 upregulation; (3) SIRT2 reversibly modulated NFL crotonylation and ubiquitination at lysine 272 (NFLK272); (4) Increased NFL ubiquitination promoted its degradation, reducing neurofibrillary tangle (NFT) formation, which colocalizes with NFL in the brain. These results suggest that long-term atorvastatin enhances NFL ubiquitination through SIRT2-mediated reversible regulation at NFLK272, leading to reduced NFT pathology and improved cognitive function. These findings not only redefine the pleiotropic neuroprotective actions of statins but also nominate SIRT2-mediated PTM interplay as a druggable node for mitigating neurofilamentopathy-driven cognitive decline.

## Background

Preventing and ameliorating cognitive decline in the aging process is a great challenge for an aging society due to the fact that cognitive decline is one of the most obvious features of aging [1]. As early as the third decade of life, human core cognitive abilities begin to decline, including processing speed, reasoning, situational memory, and spatial visualization [2]. Multivariate growth curve models show that cognitive decline is not a sharp decline in old age, but rather a small, sustained decline throughout the lifespan [3]. Furthermore, neuropathological changes were found to occur 20 years or more before the onset of dementia symptoms, which were difficult to reverse once they had occurred [4]. Therefore, early initiation and long-term intervention strategies should be desired to prevent and ameliorate cognitive decline due to aging.

Prior studies have suggested the potential benefit of statins in reducing cognitive decline and neurodegenerative diseases [5–8]. However, epidemiological studies investigating the impact of statins on cognitive function have yielded divergent findings in recent years. A large-scale epidemiological study found that statins could not reduce the risk of cognitive decline or dementia, and there was no significant difference in risk outcomes between hydrophilic and lipophilic statins [9]. Several factors likely contribute to the discrepancies observed in epidemiological research. The primary reason may be that the enrolled populations were generally older, and the observation periods were relatively short. Many participants already exhibited cognitive decline at enrollment, making it difficult to assess the effect of statins on disease progression. Lipophilic statins like atorvastatin exhibit enhanced blood-brain barrier permeability compared to hydrophilic analogs, as established by early transport studies [10]. Direct evidence confirms brain penetration of orally administered atorvastatin, with detectable brain levels via LC-MS/MS and functional modulation of neural gene expression and hippocampal signaling pathways in chronic rodent models [11, 12]. In clinical practice, statin therapy is initiated for a substantial number of individuals during middle age, primarily to lower cholesterol or stabilize plaques, not because of declining cognitive ability. To simulate a clinical scenario, we recently explored the effect of the long-term atorvastatin intervention on cognitive decline in naturally aging rats. The results showed that long-term high-dose atorvastatin can improve cognitive impairment in naturally aging rats, possibly through reducing the inflammatory response of the nervous system by regulating the intestinal flora and the metabolism of retinoic acid [13].

Protein post-translational modifications (PTMs) are critical for governing protein expression, localization, functions, and interactions with other cellular molecules, which notably build up the diversity and complexity of the proteome [14]. Lysine crotonylation is a recently discovered PTM of histone proteins that can be involved in various biological pathways, including cell metabolism, cell cycle regulation, and cellular reorganization [15]. Previous studies have reported that crotonylation may play a role in modulating epigenetics in the context of cognitive dysfunction [16]. In our preliminary screening of multiple relevant PTMs using Western blot, we identified lysine crotonylation as exhibiting the most pronounced alterations following atorvastatin treatment. This study will characterize proteomic and histological changes in crotonylation within naturally aged rat models following long-term atorvastatin intervention. By defining how these modifications regulate cognitive aging pathways, we aim to establish a mechanistic foundation for optimizing early initiation, sustained administration, and dosing of atorvastatin in dementia prevention strategies, while generating clinically translatable evidence for cognitive resilience interventions [13].

## Results

### NFL was selected as the target protein by combining proteomics and bioinformatics analysis

**Fig. 1:**
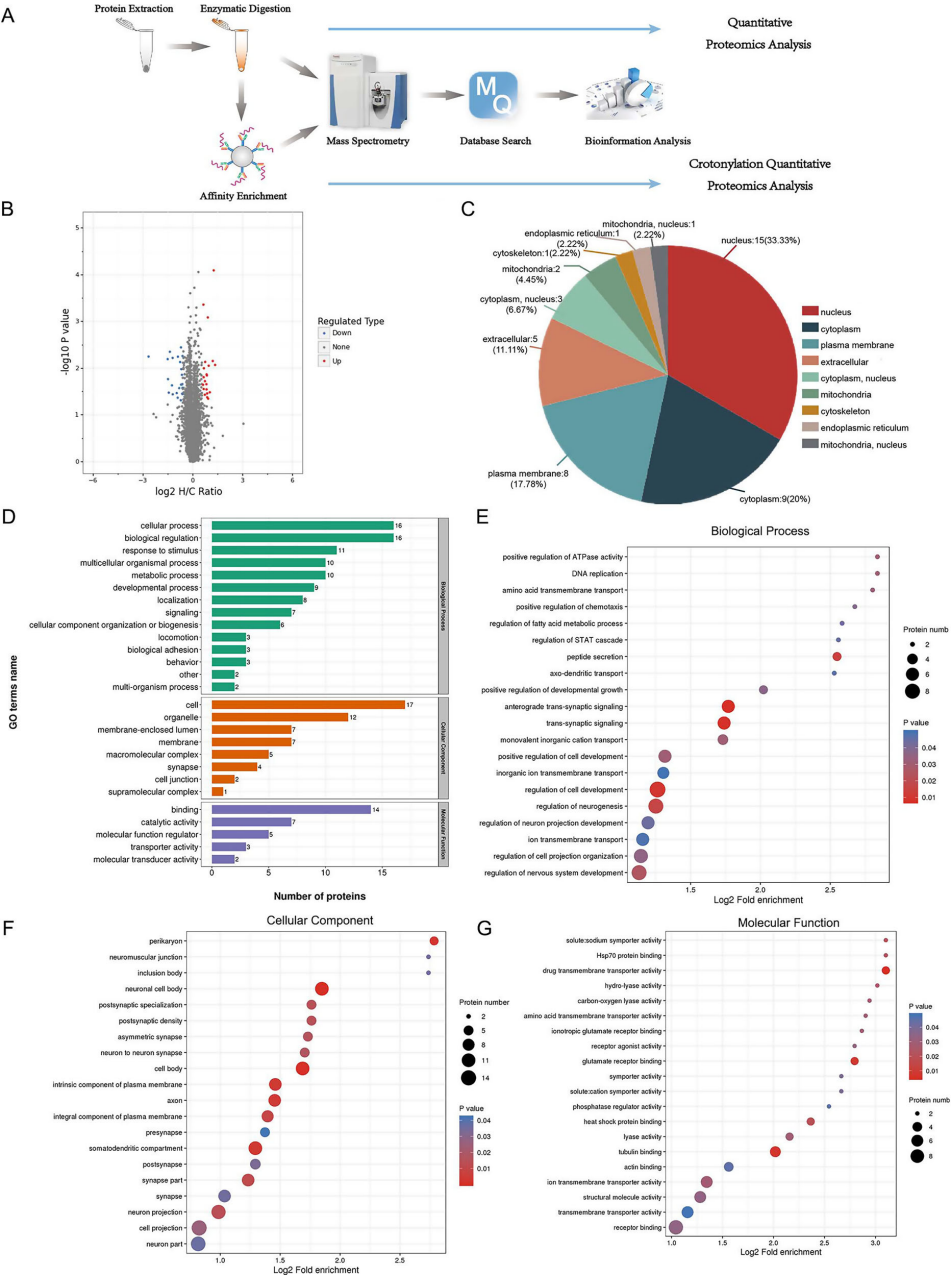
Analysis of differential protein expression and functional enrichment in response to high-dose treatment. **A** Technical wiring diagrams. **B** Quantitative volcano plot of differentially expressed proteins in the high-dose and control groups. **C** Classification of differential protein subcellular structural localization. **D** Statistical distribution of differentially expressed proteins in gene ontology (GO) secondary classification. **E**–**G** GO enrichment analysis of differentially expressed proteins. Containing three main categories: biological process, cellular component, and molecular function.

We performed a proteomic study of rat brain tissue from atorvastatin intervention and control groups using mass spectrometry analysis (Fig. 1A). A total of 6793 proteins were identified, and 5992 proteins could be quantified using mass spectrometry. A threshold of change was established for determining significant up-regulation or down-regulation: a change in differential expression exceeding 1.5 was considered significant up-regulation, while a change below 1/1.5 was considered significant down-regulation. We identified a total of 21 proteins with significantly up-regulated expression and 24 proteins with significant down-regulated expression in the atorvastatin intervention group compared to the control group (Fig. 1B, Table 1). These differential proteins hold potential significance in understanding the impacts of the atorvastatin treatment.

**Table 1:** Differential protein between the high-dose group and the control group.

Gene name	H/C ratio	H/C *P* value	Regulated type
Gh1	0.0005	0.001588	Down
Mpz	0.1599	0.005682	Down
Prph	0.3545	0.006447	Down
Slc6a5	0.3598	0.017268	Down
Avil	0.3721	0.033647	Down
Scg2	0.3886	0.004497	Down
Dnajb14	0.4358	0.006086	Down
Wrnip1	0.4383	0.036539	Down
Map1a	0.5178	0.034354	Down
Adcy6	0.5388	0.043297	Down
Sncg	0.544	0.005713	Down
Cox6c2	0.5748	0.026924	Down
Acad11	0.5967	0.007448	Down
Rpl36a	0.6149	0.048902	Down
Anlnl1	0.6179	0.003621	Down
Slc7a11	0.6193	0.022689	Down
NFM	0.628	0.010571	Down
NFH	0.6436	0.013983	Down
NFL	0.6716	0.027937	Down
Ap1s2	0.6496	0.035402	Down
Dipk1b	0.6546	0.022137	Down
Ggt1	0.6581	0.036102	Down
Hebp2	0.6584	0.026656	Down
Pola2	0.66	0.005531	Down
Uros	1.5566	0.02283	Up
Zbtb20	1.5773	0.00044	Up
Bmp2k	1.5932	0.015732	Up
Pbrm1	1.596	0.028139	Up
Ankrd63	1.6074	0.010132	Up
P2rx4	1.6753	0.03726	Up
Slc6a3	1.68	0.019144	Up
Cdk5r1	1.6984	0.007414	Up
Mapk9	1.7878	0.022177	Up
Dnajc2	1.7986	0.028626	Up
Mpped2	1.8005	0.013699	Up
Uvrag	1.8235	0.014427	Up
Gabra6	1.8425	0.034971	Up
Dnajb5	1.8473	0.042234	Up
Grid2ip	1.8938	0.000825	Up
Cdh4	1.9179	0.045335	Up
Pde8a	1.9652	0.008918	Up
Fat2	2.0614	0.033073	Up
Ppp1r17	2.3303	0.007072	Up
Car7	2.5656	0.008591	Up

The differentially expressed proteins were analyzed for subcellular structural localization and were classified statistically. The analysis revealed that these proteins are primarily located in the nucleus, cytoplasm, and plasma membrane (Fig. 1C). The differential proteins were subjected to GO functional annotation. In the biological process category, the proteins most abundant were associated with the regulation of nervous system development. In the cellular component category, the proteins most prevalent were related to the neuronal cell body. Within the molecular function category, the proteins most abundant were linked to tubulin binding (Fig.1D–G). Through GO enrichment analysis, we found that neurofilament light chain protein (NFL) was significantly different and involved in multiple functions across all three fractions (Table 2).

**Table 2:** Gene ontology enrichment analysis results.

GO term	GO terms description	Related proteins
Cellular Component	neuronal cell body	A0A0G2K0T6 **F1LRZ7** F1M7P4 **P12839 P19527** P23977 P30191 P34926 P35234 Q6P727 P51577 P61810
Cellular Component	cell body	A0A0G2K0T6 **F1LRZ7** F1M7P4 **P12839 P19527** P23977 P30191 P34926 P35234 Q6P727 P51577 P61810
Cellular Component	neurofibrillary tangle	**F1LRZ7 P12839 P19527**
Cellular Component	intermediate filament	**F1LRZ7** F1M7P4 **P12839 P19527**
Cellular Component	neurofilament	**F1LRZ7 P12839 P19527**
Cellular Component	perikaryon	**F1LRZ7 P12839 P19527** P35234 Q6P727
Cellular Component	somatodendritic compartment	A0A0G2K0T6 **F1LRZ7** F1M7P4 **P12839 P19527** P23977 P30191 P34926 P35234 Q6P727 P51577 P61810
Molecular Function	toxic substance binding	**F1LRZ7 P12839 P19527**
Molecular Function	tubulin binding	A0A0G2K0T6 **F1LRZ7 P12839 P19527** P34926 P61810
Biological Process	neurofilament bundle assembly	**F1LRZ7 P12839 P19527**
Biological Process	neurofilament cytoskeleton organization	**F1LRZ7 P12839 P19527**
Biological Process	cellular response to estradiol stimulus	**F1LRZ7 P12839 P19527**
Biological Process	axon regeneration	**F1LRZ7 P12839 P19527**

P19527:NFL, F1LRZ7:NFM, P12839:NFH.

### Long-term atorvastatin intervention reduces the level of crotonylation modification at the NFL K272 locus in the brain tissue of naturally aging rats

**Fig. 2:**
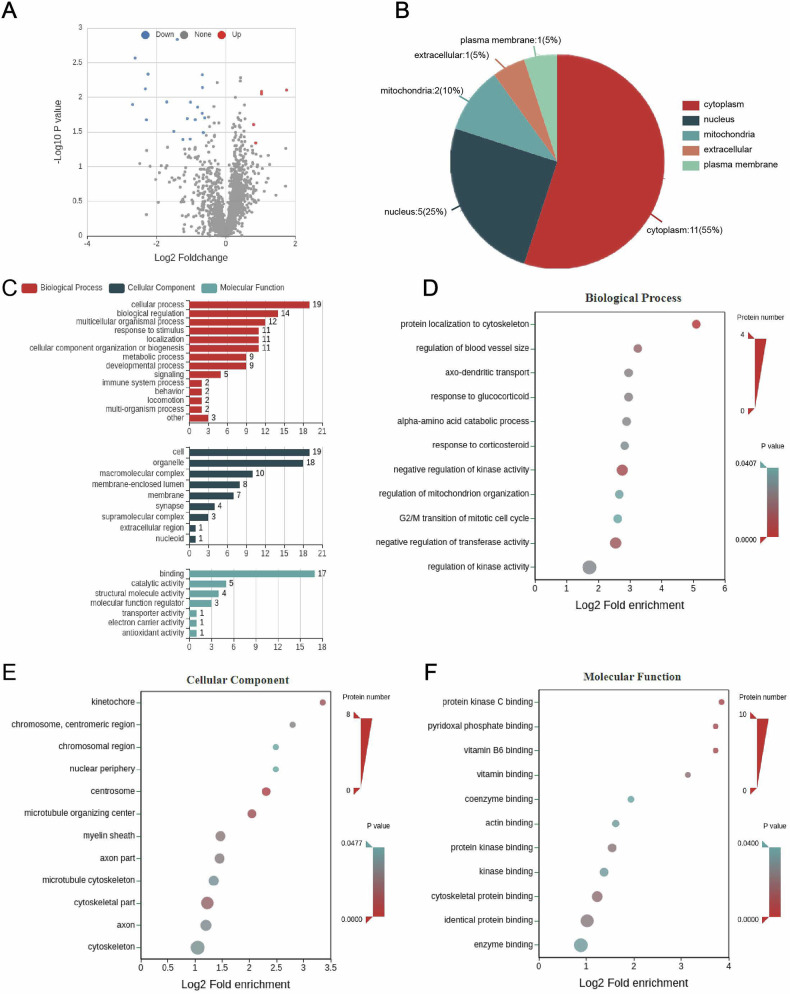
Crotonylomic analysis of high-dose treatment effects. **A** Quantitative volcano plot of differentially expressed modification sites for crotonylation modification in the high-dose group and control group. **B** Classification of subcellular structural localization. **C** Statistical distribution of differentially expressed proteins with crotonylation modifications in GO secondary classification. **D**–**F** GO enrichment analysis of differentially expressed proteins modified by crotonylation. Containing three main categories: biological processes, cellular components, and molecular functions.

We examined the histology of crotonylation modifications in brain tissue from the hippocampal region of atorvastatin- and saline-treated rats using mass spectrometry, and a total of 17 significantly different proteins for crotonylation modifications were screened in the atorvastatin intervention group compared with the control group (Table 3, Fig. 2A). The proteins differentially expressed by crotonylation modifications were analyzed for subcellular structural localization and statistically classified. The analyses showed that these proteins were mainly located in the cytoplasm, nucleus, and mitochondria (Fig. 2B). The differentially expressed proteins underwent GO functional annotation and enrichment analysis. The functional annotation results revealed that, in the biological process category, proteins associated with the regulation of kinase activity were the most abundant. In the cellular component category, proteins related to the cytoskeleton were the most prevalent. Within the molecular function category, proteins linked to enzyme binding were the most abundant (Fig. 2C–F). Based on the above proteomics findings, NFL was selected as a target protein for further study, and a search of the crotonylation modification histology database revealed a significant down-regulation of crotonylation at the NFL K272 locus (Table 3).

**Table 3:** Results of crotonylation modification in high-dose and control groups.

Gene name	Position	H/C ratio	H/C *P* value	Regulated type
Dctn2	K140	0.379	0.001467215	Down
LOC103692829	K59	0.656	0.019823035	Down
Map1a	K734	0.49	0.040190238	Down
Map1a	K1809	0.463	0.020320802	Down
Sptbn1	K302	0.627	0.007242427	Down
Tppp	K77	1.818	0.045607475	Up
Snx6	K359	0.568	0.013856573	Down
Ndufv3	K57	0.626	0.004733513	Down
Pvalb	K28	0.2	0.007573402	Down
Alb	K264	2.039	0.008946452	Up
Alb	K103	1.746	0.024736999	Up
Qdpr	K99	0.424	0.040639018	Down
NFL	K272	0.353	0.031059295	Down
Cbs	K386	0.624	0.017057574	Down
Stx1b	K125	0.204	0.021150783	Down
Stx1b	K188	0.163	0.002711647	Down
Ywhag	K120	0.155	0.012714139	Down

### Long-term high-dose atorvastatin intervention reduces protein expression of NFL in brain tissue and blood

**Fig. 3:**
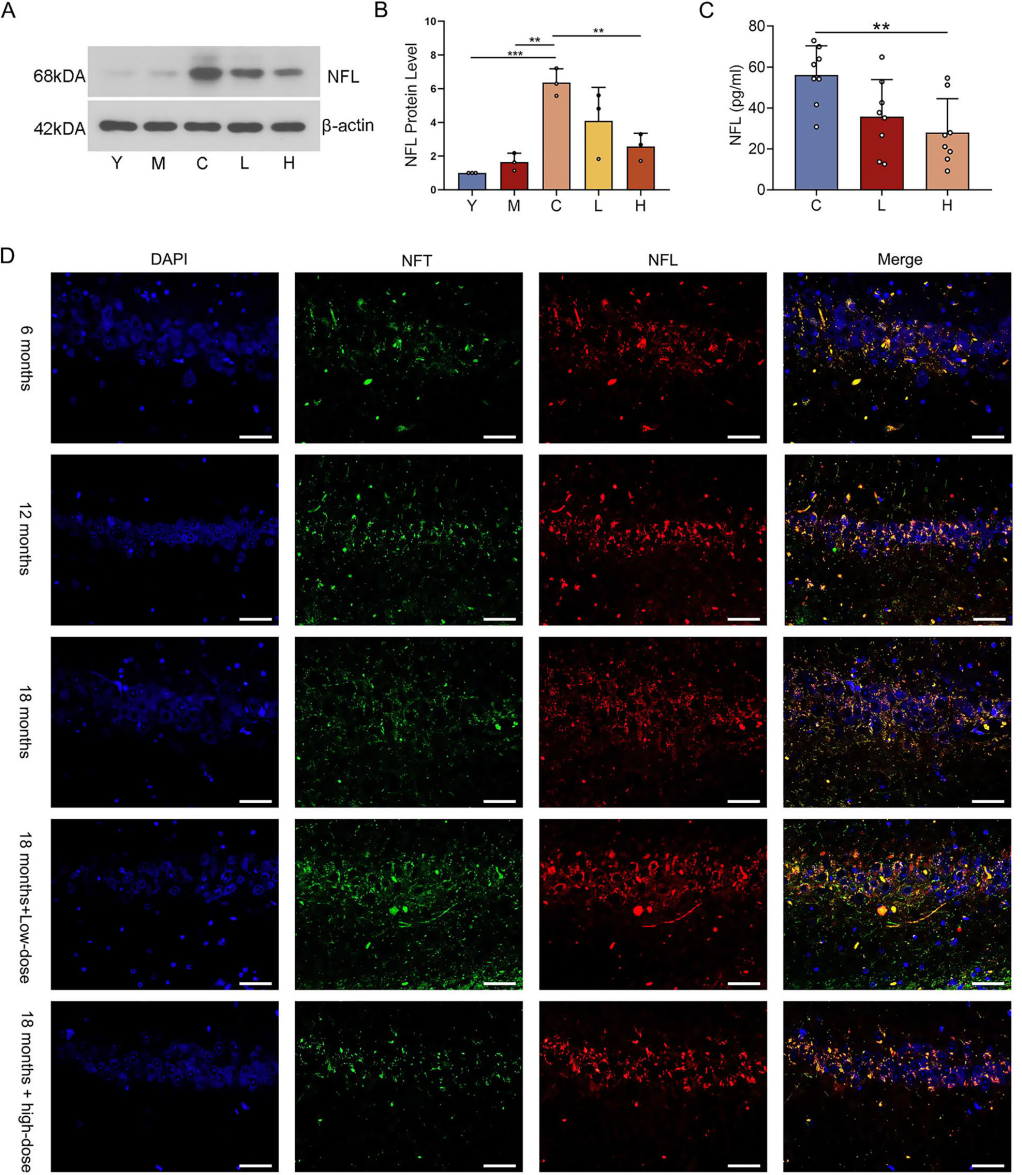
Effects of atorvastatin on NFL in the aging rat brain and serum. **A**, **B** Relative protein levels of NFL in the brain tissue of each group. Y: Youth group (6-month-old rats); M: Middle-aged group (12-month-old rats); C: Aged group (18-month-old rats); L: Low-dose atorvastatin intervention in the aged group; H: High-dose atorvastatin intervention in the aged group. Data are expressed as mean ± standard deviation (*n* = 3 per group) and were analyzed using one-way ANOVA and LSD-*t* test. *P < 0.05, **P < 0.01, ***P < 0.001. **C** Determination of NFL in the serum of each group by the method of Simoa. Data are expressed as mean ± standard deviation (*n* = 8 per group) using one-way ANOVA and LSD-*t* test. **P < 0.01. **D** Immunofluorescence staining: Localization of NFL and NFT immunofluorescence double staining in different groups of hippocampal regions, photographed at 400× mirror, scale length 50 μm, NFT (green light), NFL (red light).

The changes in protein expression of NFL in each group in the above histological results were verified by Western blot. To further clarify the changes in the NFL in brain tissue at different ages, two additional groups of different ages were added as controls: the young (Y) group (6-month-old rats) and the middle-aged (M) group (12-month-old rats). It was found that the expression of NFL in group C was significantly up-regulated compared with group Y and group M (Fig. 3A, B, *P* < 0.001), which suggests that the expression of NFL gradually increases with age. Compared with control, long-term high-dose atorvastatin treatment significantly down-regulated NFL expression (Fig. 3A, B, group H vs group C, *P* < 0.01). To better investigate the alteration of NFL in the aging rat model, we applied the fourth generation of single-molecule array (Simoa) assay technology to detect NFL in blood. Simoa results showed that the serum NFL protein content was significantly down-regulated in group H compared with group C (Fig. 3C, *P* < 0.01).

### Immunofluorescence staining reveals high overlap and co-variation of NFL and neurofibrillary tangles (NFT)

To investigate the relationship and change between NFL and NFT, immunofluorescence double staining was applied to detect co-localization of NFL with NFT in the hippocampus of brain tissues. The results revealed that both NFL and NFT in the hippocampus increased with age, with the highest in the 18-month-old rat group, which was significantly decreased by high-dose atorvastatin administration (Fig. 3D).

### Long-term atorvastatin intervention down-regulates crotonylation levels and up-regulates ubiquitination levels in the NFL

**Fig. 4:**
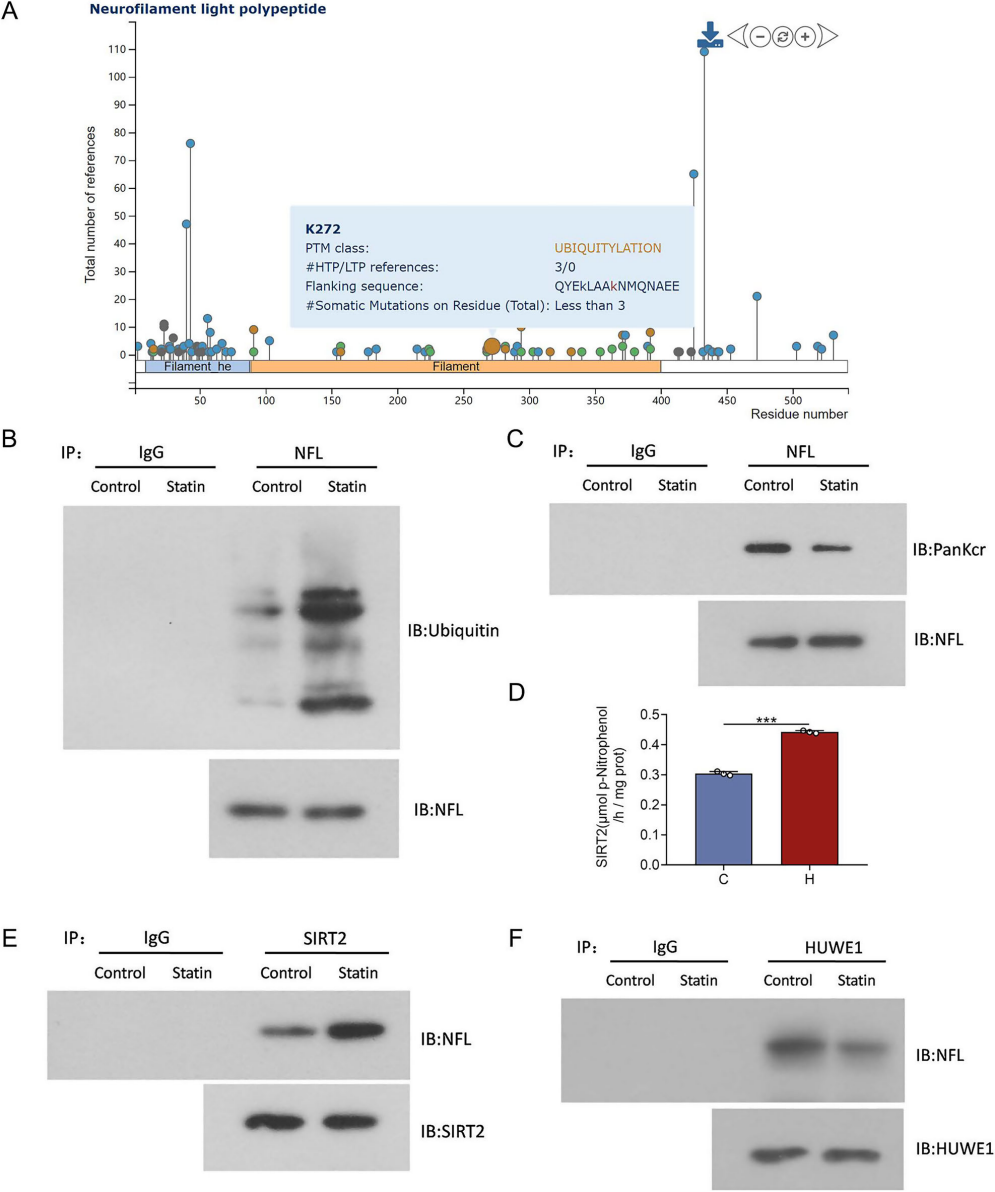
Effects of long-term atorvastatin intervention on NFL modification levels and associated proteins. **A** The presence of ubiquitination modifications at the K272 locus of NFL was identified by database PhosphoSitePlus analysis. **B** Long-term atorvastatin intervention was found to up-regulate the level of ubiquitination modification of NFL by Co-IP. **C** Long-term atorvastatin intervention was found to down-regulate the level of crotonylation modification of NFL by Co-IP. **D** Long-term atorvastatin intervention increases the enzymatic activity of SIRT2. **E** Long-term atorvastatin intervention was found to improve the interactions between SIRT2 and NFL by Co-IP. **F** Long-term atorvastatin intervention did not affect HUWE1-NFL interactions found by Co-IP approach. *P < 0.05, **P < 0.01.

To explore the reasons for the reduced NFL expression, we speculated that it might be related to the altered level of ubiquitination modification of the NFL. In this study, Co-IP experiments for ubiquitination were performed in the elderly control group and the high-dose atorvastatin intervention group. The results suggested that long-term atorvastatin intervention could increase the level of ubiquitination modification in NFL (Fig. 4B). Co-IP experiments of crotonylation were also performed on the elderly control group and the high-dose atorvastatin intervention group. The results suggest that long-term atorvastatin intervention can reduce the level of crotonylation modification in NFL (Fig. 4C).

### Co-IP combined with mass spectrometry analysis screened SIRT2 and HUWE1 can interact with NFL

To investigate the reasons for the changes in the levels of NFL crotonylation and ubiquitination modifications, we mined upstream regulatory enzymes that may regulate both modifications by combining mass spectrometry. We detected proteins interacting with NFL by Co-IP combined with mass spectrometry. It was found that 1220 interacting proteins were identified in the control group, and 1322 interacting proteins were identified in the high-dose group. To clarify the reasons for the reduced protein expression of NFL and the down-regulation of its crotonylation modification levels, the analysis of the database revealed that there was also a concomitant ubiquitination modification at the K272 locus of NFL (Fig. 4A). To search for the upstream proteins affecting its crotonylation modification and ubiquitination modification, SIRT2, a deacetylase associated with crotonylation, and HUWE1, an E3 ligase associated with ubiquitination, were found as a result of Co-IP co-mass spectrometry analysis.

### Long-term atorvastatin intervention increased SIRT2 interactions with the NFL, and did not significantly affect HUWE1 interactions with the NFL

We have screened the protein SIRT2, which has an interacting relationship with NFL, by Co-IP combined with mass spectrometry analysis. Verification of whether atorvastatin regulates crotonylation modification of NFL via SIRT2 by Co-IP. The results showed a higher level of SIRT2 and NFL interactions in the high-dose group (Fig. 4E). The enzyme activity of SIRT2 was also assayed, and the results showed that the enzyme activity was significantly higher in the high-dose group compared with the control group (Fig. 4D, *P* < 0.01). The homology was continued to verify whether atorvastatin modulates ubiquitination modification of NFL through HUWE1. The results showed no significant change in the bands in the high-dose group compared to the control group (Fig. 4F), suggesting that long-term atorvastatin does not increase the ubiquitination level of the NFL by modulating HUWE1.

### Inhibition or overexpression of SIRT2 in neuronal cells both modulate the level of crotonylation modifications in the NFL, which in turn affects the level of ubiquitination

**Fig. 5:**
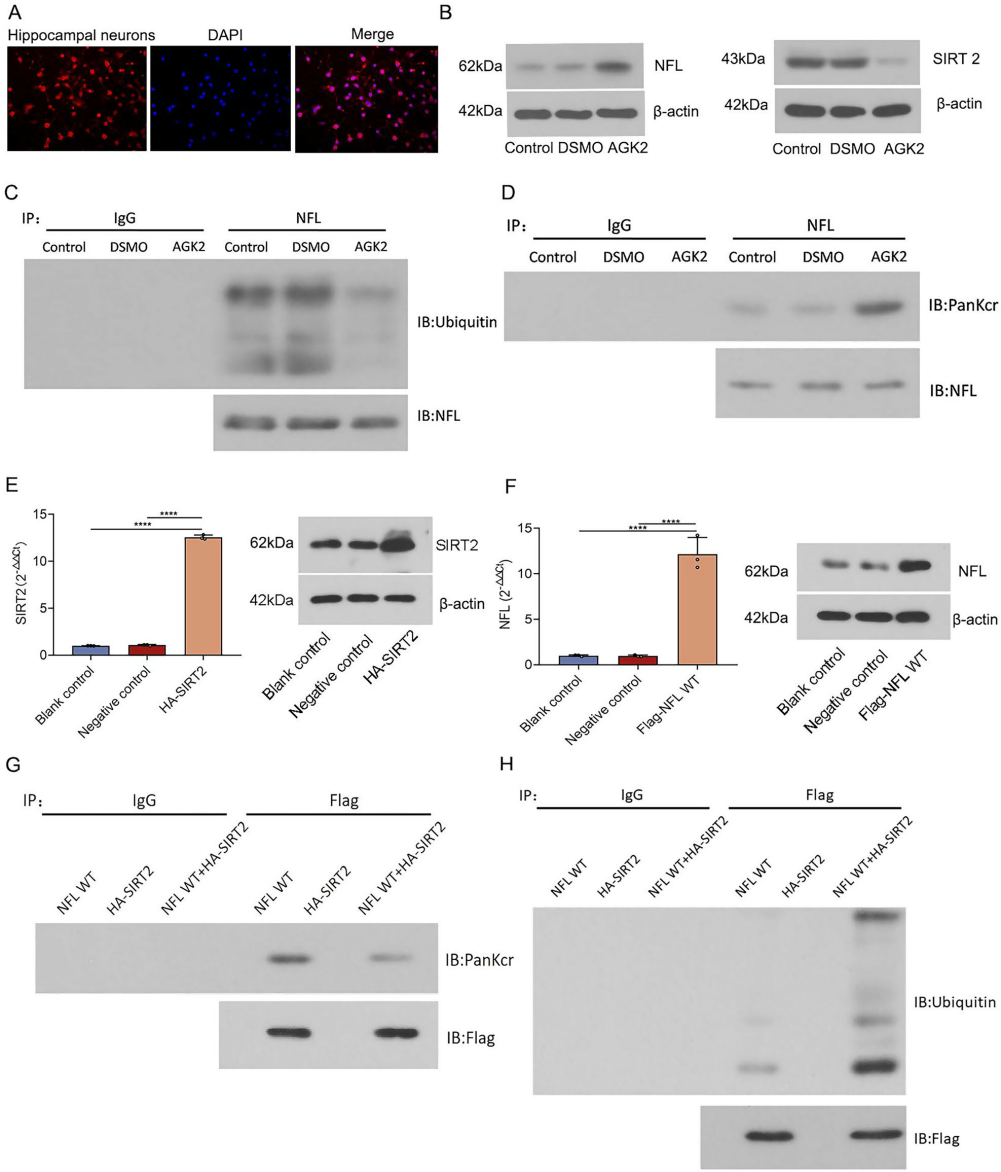
Effects of SIRT2 inhibition on NFL ubiquitination and crotonylation in hippocampal neurons. **A** Neuronal purity was determined by immunofluorescence detection of the expression of the neuron-specific marker MAP2 in hippocampal neurons cultured for 9 days. **B** Western blot detected a significant decrease in SIRT2 expression level and a significant increase in NFL expression after AGK2 intervention in hippocampal neuronal cells. **C** Decreased ubiquitination level of NFL was detected by Co-IP in AGK2-treated rat hippocampal neuronal cells. **D** Increased level of crotonylation of NFL was detected by Co-IP in AGK2-treated rat hippocampal neuronal cells. **E** Real-time PCR to detect the mRNA expression level of SIRT2 in cells; Western blot detection of protein concentration levels of SIRT2 in cells. **F** Real-time PCR to detect the mRNA expression level of NFL in cells; Western blot detection of protein concentration levels of NFL in cells. **G** The level of crotonylated modification of NFL in each group of cells was detected by Co-IP. **H** The level of ubiquitination modification of NFL in each group of cells was detected by Co-IP.

Purified neurons were obtained by isolating and culturing bilateral hippocampal tissues from neonatal SD rats within 24 h. Neuronal purity was determined by immunofluorescence detection of the expression of the neuron-specific marker MAP2 in hippocampal neurons cultured for 9 days (Fig. 5A). Hippocampal neuronal cells were treated with AGK2, a SIRT2-specific inhibitor. Western blot detection of SIRT2 expression levels showed that SIRT2 expression levels were reduced in the AGK2 group compared to the control and solvent control groups (Fig. 5B). Western blot detection of NFL expression levels showed that NFL expression levels were increased in the AGK2 group compared to the control and solvent control groups (Fig. 5B). The levels of crotonylation modification and ubiquitination modification of NFL in the cells of each group were identified by Co-IP, and the results showed that the AGK2-treated group had higher levels of crotonylation modification and lower levels of ubiquitination modification compared to the control group and the solvent control group (Fig. 5C, D).

SIRT2 overexpressing lentivirus (HA-SIRT2) and negative control empty lentivirus were constructed. The mRNA expression level of SIRT2 was detected by Real-time PCR at 24 h of infection, and the protein concentration level of SIRT2 was detected by Western blot at 48 h. The results showed that the SIRT2 overexpression lentivirus was successfully constructed and infected rat hippocampal neuronal cells (Fig. 5E). Rat hippocampal neuronal cells were taken, and the cells were infected with rat NFL overexpressing lentivirus (NFL WT) or negative control lentivirus. The same method was validated to show that the NFL overexpressing lentivirus had been successfully constructed and successfully infected rat hippocampal neuronal cells (Fig. 5F).

The level of crotonylation modification of NFL in each group of cells was identified by Co-IP. The results showed that the band of NFL WT + HA-SIRT2 was lighter as compared to that of NFL WT, which indicates that the crotonylation of NFL WT + HA-SIRT2 has a lower level (Fig. 5G). This suggests that the level of crotonylation in NFL of hippocampal neuronal cells decreased after SIRT2 overexpression. The same method was used to determine the level of ubiquitination modification of NFL in each group. The results showed darker bands in the NFL WT + HA-SIRT2 compared to the NFL WT, indicating a higher level of ubiquitination in the NFL WT + HA-SIRT2 (Fig. 5H). This suggests that the ubiquitination level of NFL in hippocampal neuron cells increased after SIRT2 overexpression.

### Atorvastatin affects crotonylation levels and ubiquitination levels in NFL via SIRT2

**Fig. 6:**
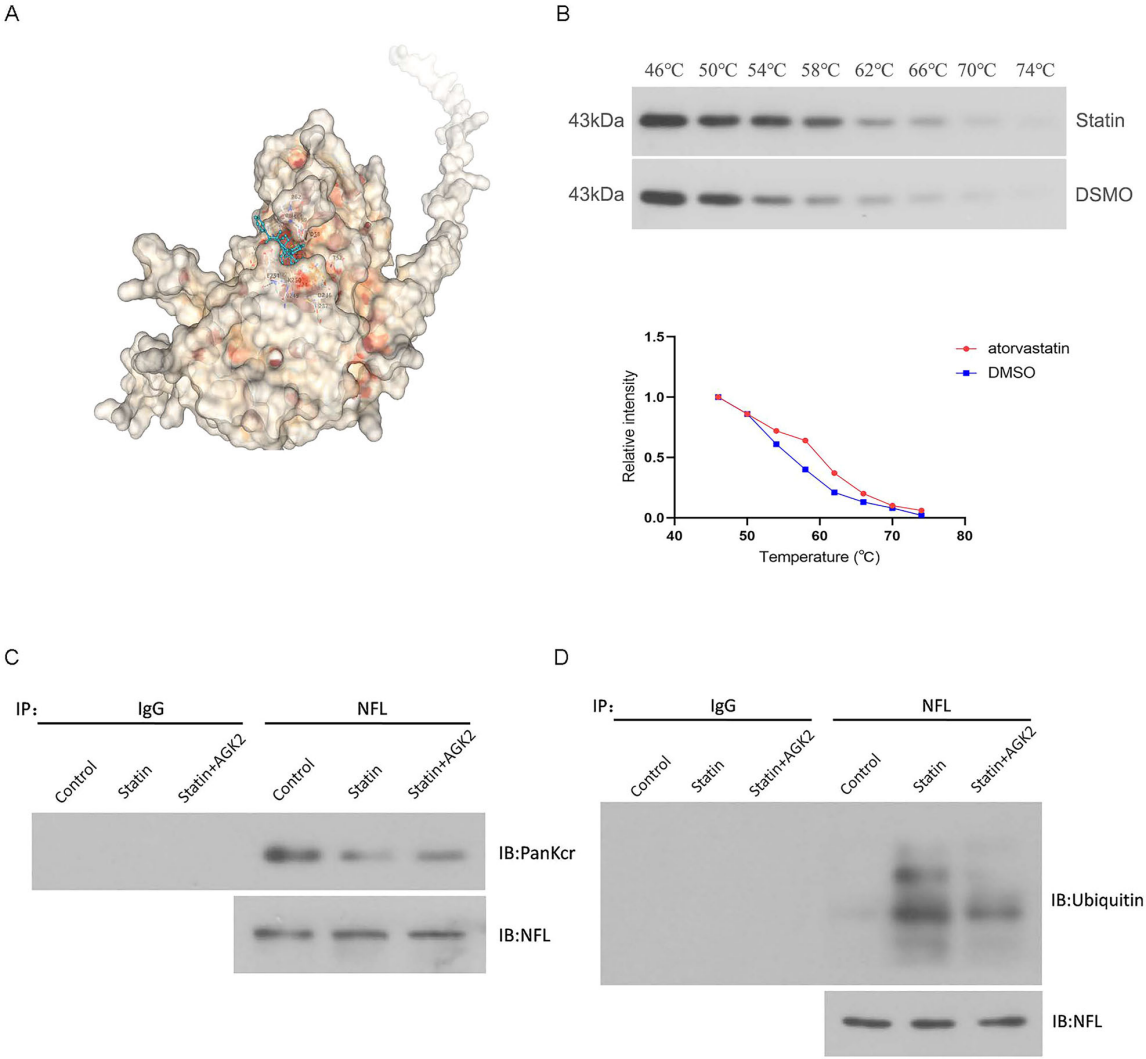
Atorvastatin affects the level of crotonylation and ubiquitination in NFL via SIRT2. **A** The CB-DOCK2 molecular docking results revealed that atorvastatin interacts with 31 amino acid residues of SIRT2. **B** WB and graph analysis of cellular thermal shift assay. **C** The level of crotonylated modification of NFL in each group of cells was detected by Co-IP. **D** The level of ubiquitination modification of NFL in each group of cells was detected by Co-IP.

The CB-DOCK2 molecular docking results revealed that atorvastatin interacts with 31 amino acid residues of SIRT2, including ALA48, ILE50, THR52, HIS150, GLU251, ASP286, and others (Fig. 6A). Additionally, CETSA experiments demonstrated that atorvastatin enhances the thermal stability of SIRT2 (Fig. 6B). These findings suggest that SIRT2 may be a potential drug target of atorvastatin.

To verify whether atorvastatin affects the crotonylation level of NFL via SIRT2, we identified the crotonylation modification level of NFL in each group using the Co-IP method. The results showed that the bands in the statin group were lighter than those in the control group. This indicated that the intervention of atorvastatin reduced the crotonylation level of NFL. The bands in the statin+AGK2 group were darker than the statin group (Fig. 6C). This suggests that the ability of atorvastatin to down-regulate NFL crotonylation modification was reduced after AGK2 inhibition of SIRT2. This suggests that atorvastatin controls the level of crotonylation modification in the NFL via SIRT2.

The results of ubiquitination experiments showed darker bands in the statin group than in the control group, and lighter bands in the statin+AGK2 group than in the statin group (Fig. 6D). This suggests that the ability of atorvastatin to up-regulate NFL ubiquitination modifications was reduced after AGK2 inhibition of SIRT2. This part of the results suggests that atorvastatin can indirectly affect the ubiquitination modification of NFL through the direct modulation of NFL crotonylation modification by SIRT2.

### SIRT2 regulates NFL ubiquitination through the NFL K272 locus

**Fig. 7:**
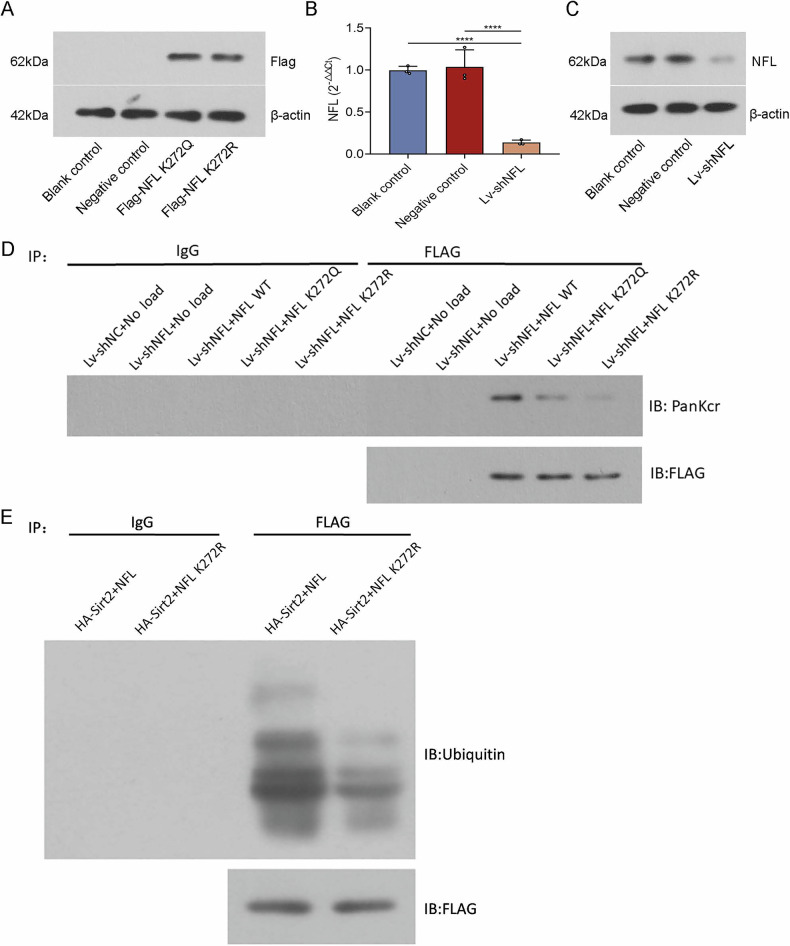
Probing changes in crotonylation levels and ubiquitination levels in the NFL after mutation at the NFL K272 locus. **A**–**C** Real-time PCR and Western blot were used to verify that successful constructs have been constructed to construct mutant lentivirus 1 (NFL K272Q), mutant lentivirus 2 (NFL K272R), Lv-shNFL, and Lv-shNC. **D** The level of crotonylated modification of NFL was detected by CO-IP in each group of cells. **E** SIRT2 regulates through the NFL K272 locus, the NFL’s Ubiquitination level: the level of ubiquitination modification of NFL in each group of cells was detected by Co-IP. ****P < 0.0001. NFL K272Q: mutant lentivirus 1; NFL K272R: mutant lentivirus 2; NFL WT: wild-type NFL group; Lv-shNC: NFL control lentivirus; Lv-shNFL: NFL-infected lentivirus; HA-SIRT2: rat Sirt2 overexpressing lentivirus.

The expression level of Flag in the cells of each group was detected by Western blot. The results showed no bands in the blank control and negative control groups, and bands in the NFL K272Q and NFL K272R groups (Fig. 7A). This indicates that NFL K272Q as well as NFL K272R have been successfully constructed. Validation by Real-time PCR and Western blot indicated that Lv-shNFL and Lv-shNC had been successfully constructed (Fig. 7B, C).

The level of crotonylation modification of NFL in each group of cells was identified by Co-IP. Compared with Lv-shNFL+NFL WT, Lv-shNFL + NFL K272Q, and Lv-shNFL + NFL K272R have lighter bands, which suggests that the level of crotonylation of NFL decreases after mutation of the NFL K272 locus (Fig. 7D). This suggests that the NFL K272 locus has a greater effect on the regulation of crotonylation level.

The level of ubiquitination modification of NFL in each group of cells was identified by Co-IP. The results showed that the bands of HA-SIRT2 + NFL K272R were shallower compared with those of HA-SIRT2 + NFL, which indicated that the ubiquitination level of HA-SIRT2 + NFL K272R had a lower level of ubiquitination (Fig. 7E). This suggests that the level of ubiquitination modification of NFL can be up-regulated after overexpression of SIRT2. However, after mutation at the NFL K272 locus, the degree of increase in the level of ubiquitination modification caused by up-regulation of SIRT2 was significantly reduced. In conclusion, this suggests that SIRT2 regulates the level of ubiquitination modification in the NFL through the NFL K272 locus.

## Discussion

Our recent study found that long-term atorvastatin intervention produced a significant improvement in cognitive function in naturally aging rats [9]. To gain insight into the potential mechanisms underlying the cognitive decline-alleviating effects of atorvastatin, we conducted a comprehensive proteomic examination of rat brain tissue. Our proteomics and modification histology studies showed that long-term atorvastatin significantly down-regulated the expression of neurofilament light chain (NFL) and its crotonylation modification level in the brain tissues of naturally aging rats. Additionally, our study uncovered a significant down-regulation of the lysine crotonylation (Kcr) modification at the K272 site of NFL in the group of rats receiving long-term atorvastatin intervention when compared to the control group. Subsequently, we found concomitant ubiquitination modifications at the K272 locus of NFL by PhosphoSitePlus database prediction, and the level of ubiquitination modifications in NFL was elevated after long-term atorvastatin intervention. These findings collectively indicate that the NFL protein likely undergoes crotonylation and ubiquitination modifications at the K272 site. Biochemical evidence indicates that ubiquitination serves as the predominant pathway mediating proteasomal degradation of NFL [17]. The elevated ubiquitination at this site appears to predominantly contribute to reduced NFL protein expression, potentially linking to the cognitive decline-alleviating effects of atorvastatin.

NFL is an intermediate filament protein of myelinated axons, and a small amount of NFL is released from axons under normal physiological conditions, but under pathological conditions, when neurons are damaged, the release of NFL is significantly increased, and the NFL in tissue fluid can diffuse through the cerebrospinal fluid into the blood [18, 19]. Its elevated levels in cerebrospinal fluid and blood are proportional to the extent of axonal damage in various neurological disorders, including neurodegenerative, inflammatory, traumatic, and cerebrovascular diseases [20]. Increasing evidence suggests that the NFL can be used as a biomarker for the diagnosis, prognosis, and monitoring of neurological disorders and is one of the most promising biomarkers for future clinical and research studies [21]. In view of the previous histological findings, to explore the mechanism of action of atorvastatin affecting crotonylation modification and ubiquitination modification of the NFL, we performed a screening analysis of proteins interacting with the NFL by immunoprecipitation (Co-IP) coupled with mass spectrometry. In our findings, we identified the de-crotonylase SIRT2 associated with crotonylation and the E3 ligase HUWE1 associated with ubiquitination. We speculate that SIRT2 and HUWE1 may be implicated in the alterations of NFL crotonylation and ubiquitination modifications, as well as the reduction in protein expression. SIRT2, an NAD+-dependent deacetylase, exhibits wide expression across various tissues and organs, with notably higher levels in the brain, particularly in the cortex, striatum, hippocampus, and spinal cord [22, 23], which was strongly linked to anti-aging effects, including the extension of lifespan in various organisms [24]. Furthermore, SIRT2 has demonstrated the ability to ameliorate cognitive dysfunction in mouse models of neurodegenerative disease and accelerated aging [25, 26]. Cumulatively, this body of evidence indicates an active role for SIRT2 in delaying aging and enhancing cognition, while atorvastatin has been reported to up-regulate SIRT2 expression [27, 28]. Our study finds that atorvastatin enhances SIRT2 enzyme activity, facilitates the interaction between SIRT2 and NFL, and modulates the crotonylation modification level of NFL, but does not increase the level of HUWE1-NFL interactions, which is a marker of ubiquitination. It has been found that the ubiquitination process is independent of the problem of aging-induced hypo-ubiquitination, and that ubiquitin ligase activity during ubiquitination hardly changes with age [29, 30]. We therefore speculate that atorvastatin may not regulate ubiquitination levels through HUWE1, but through other pathways. PTMs can occur simultaneously on proteins, where they can positively or negatively affect each other’s behavior, called PTM crosstalk [31]. Prior studies have shown that the crotonylation and ubiquitination of histone H2A at lysine 119 (H2AK119) were reversibly regulated by replication stress. Crotonylation of H2AK119 antagonized ubiquitination on the same lysine residue during replication stress, and SIRT1 facilitated de-crotonylation of H2AK119, with ubiquitination modifications occurring for each crotonyl group removed [32]. Similar models for different modifications of the same lysine residue have been reported in the regulation of other proteins [33, 34]. Thus, we hypothesize that the competitive regulation of crotonylation modifications may be related to the elevated level of ubiquitination in NFL, which is supported by further cell experiments.

Without the NFL, the most important component of neurofilament light (NF), neither the neurofilament medium chain nor the neurofilament heavy chain can be assembled into a functional neurofilament [35]. Patients with cognitive dysfunction exhibit significant accumulation of NF in the cell bodies and proximal axons of affected neurons [18, 19]. Transgenic mouse models provide additional evidence supporting the idea that these atypical NF accumulations lead to the degeneration of affected neurons, rather than serving as a mere byproduct of the pathological process [36–38]. NF is closely linked to aging, as evidenced in studies showing alterations in the neuronal cytoskeleton in the hippocampus of aging rats, which align with increased NF protein levels [39]. The aforementioned studies indicate a significant contribution of the NFL to the pathophysiological mechanism of cognitive decline. Consequently, we investigated the expression of NFL and associated pathological alterations in the brain tissue of naturally aging rats. Our study identified co-localization of NFL and NFT in the hippocampal region of rat brain tissues. In the natural aging group, both NFL and NFT levels increased with age, whereas long-term administration of atorvastatin showed a declining trend in NFL and NFT levels. Previous studies have confirmed through immunohistochemical analysis combined with mass spectrometry analysis that NFL is an important structural component of NFT [40]. As one of the characteristic pathological changes of AD, NFT can also occur with age in elderly individuals who never develop AD, accompanied by significant cognitive decline and neurodegenerative changes [1]. This implies that age-related cognitive decline may share common pathophysiological mechanisms with AD. Taken together, we believe that atorvastatin may reduce the formation of NFT by lowering NFL, thereby improving cognitive function.

**Fig. 8:**
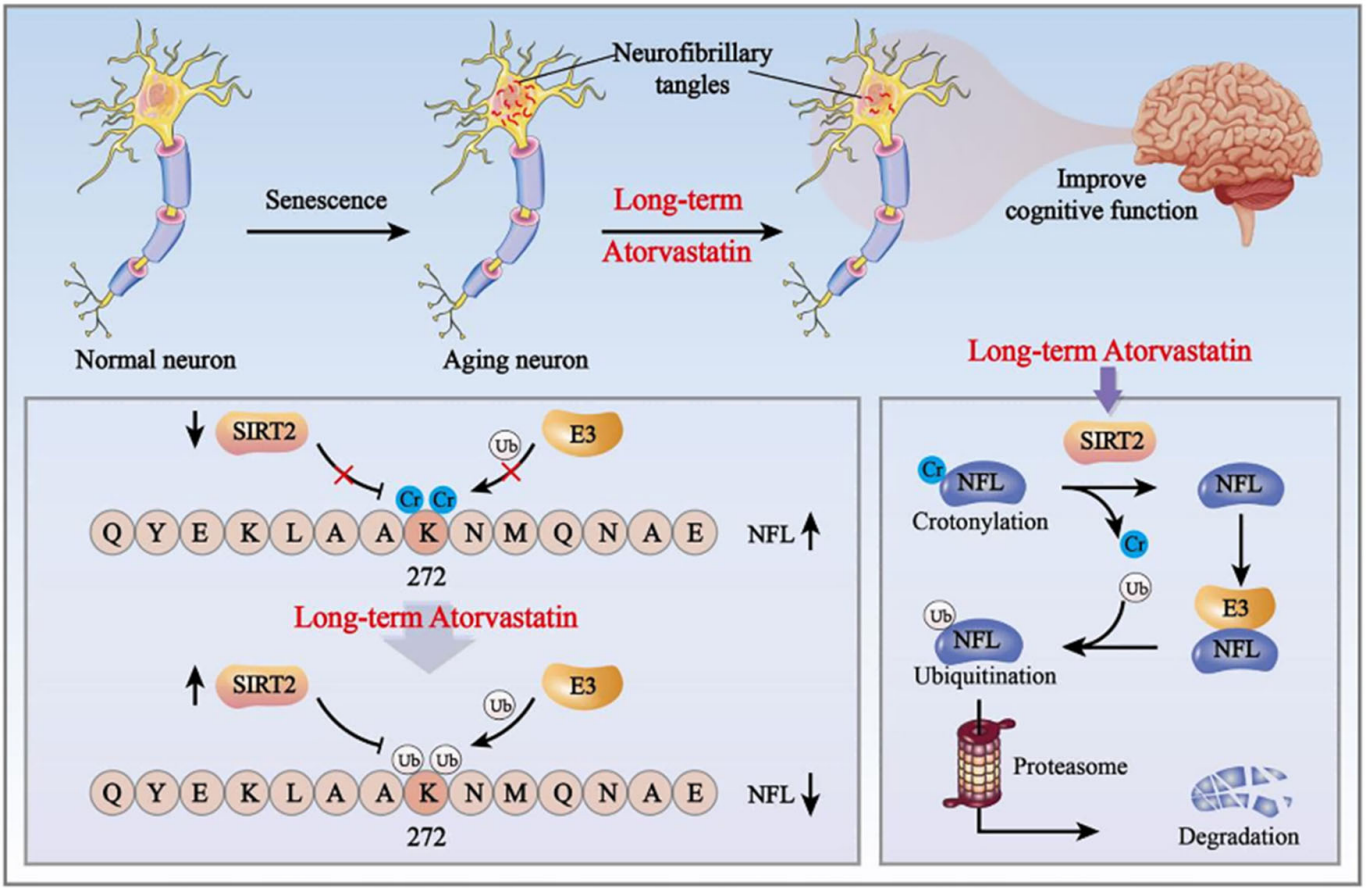
Diagram of the scientific mechanism hypothesis.

Based on the above discussions, we propose the following scientific hypothesis (Fig. 8). Previous studies and our work suggest that aging may lead to reduced ubiquitination levels of NFL [41]. Prolonged administration of atorvastatin will diminish the crotonylation modification level of NFL through the up-regulation of SIRT2, consequently facilitating a greater number of lysine residues at the K272 site to bind with ubiquitin molecules, thereby augmenting the overall level of NFL ubiquitination and facilitating the degradation of NFL protein. Ultimately, this leads to a reduction in NFT formation, which results in an improvement in cognitive function in elderly rats.

### Limitations

While our study elucidates novel mechanisms of atorvastatin-regulated NFL crotonylation in hippocampal neurons, several constraints merit consideration. First, the current study focused on examining statin effects within this defined aging window and therefore did not extend to 24 months. We fully acknowledge the importance of investigating later time points to model advanced aging better. Second, the neuronal isolation paradigm can effectively eliminate direct systemic medication confounders, but this cannot completely preclude potential modulation of neuronal proteostasis via statin-induced alterations in circulating factors that may exert pleiotropic influences. Third, although we demonstrate reduced NFL levels, the physiological relevance of this finding requires clarification through future studies establishing direct causal links between NFL dynamics and cognitive outcomes. Fourth, although both NFL and MAP1A demonstrated significant alterations in our proteomic profiling, we prioritized NFL for in-depth mechanistic investigation due to its well-documented evidence in neurodegenerative disease. In contrast, MAP1A’s direct involvement in cognitive function remains less well-characterized, which warrants further investigation with regard to its role in statin-mediated neuroprotection. Finally, the exclusive use of male cohorts precludes assessment of sex-dependent therapeutic profiles. This design choice, while justified by the need to avoid estrogen-mediated neuroprotective confounders, limits translation to female physiology. Future investigations will incorporate both sexes to comprehensively map sex-stratified responses.

In conclusion, this is the first report to suggest that long-term atorvastatin intervention may enhance the ubiquitination of NFL by upregulating SIRT2-mediated reversible regulation at NFL K272, ultimately leading to a reduction in NFT and improvement in cognitive function. These findings provide novel and important information to support the use of prolonged atorvastatin in the middle-aged population with indications of atorvastatin.

## Materials and methods

### Experimental model and subject details

Adult male SD rats (SPF level, 9 months of age) were housed in an environment with free access to food and water, a 12-h light/12-h dark cycle, and a temperature of 24 ± °C. These experiments were approved by the Animal Care and Use Committee of the General Hospital of Northern Theater Command and conformed to the principles outlined in the National Institutes of Health guidelines. The number of animals used at each step was minimized. Computer-generated block randomization stratified by weight, implemented by an independent technician. Allocation concealment via cage coding. A total of 12 rats per group were initially included in the experiment, and 2 rats per group died of age-related complications (confirmed at necropsy) during the 9-month atorvastatin intervention. No animals were excluded due to technical failures. The final cohort analyzed consisted of 10 rats per group that met the predefined inclusion criteria.

As described in our recent study [9], a total of 30 rats were randomly divided into 3 groups: (1) control (C) group (*n* = 10): intragastric administration of 0.5 ml saline each day for 9 consecutive months; (2) low-dose (L) statin group (*n* = 10): intragastric administration of atorvastatin (2.1 mg/kg, dissolved in 0.5 ml saline) each day for 9 consecutive months; (3) high-dose (H) statin group (*n* = 10): intragastric administration of atorvastatin (8.4 mg/kg, dissolved in 0.5 ml saline) each day for 9 consecutive months. Assuming a human body weight of 60 kg, the drug dose is based on the low dose (20 mg/day) and the high dose (80 mg/day). According to the equivalent dose conversion method for experimental animals, the conversion factor for SD rats was 6.3, and therefore, the low dose for SD rats was 2.1 mg/kg/day, and the high dose was 8.4 mg/kg/day. Atorvastatin is a gift from Pfizer (Pfizer Inc.). Investigators were blinded to group allocation during the animal experiments.

### Mass spectrometry

An equal amount of each sample protein was taken for enzymatic digestion, and the volume was adjusted to be consistent with the lysate. Slowly add the final concentration of 20% TCA, vortex mixing, and precipitate at 4 °C for 2 h. 4500 × *g*, centrifuge for 5 min, discard the supernatant, and wash the precipitate with pre-cooled acetone 2–3 times. After drying the precipitate, add a final concentration of 200 mM TEAB, sonicate to break up the precipitate, add trypsin at a ratio of 1:50 (protease: protein, m/m), and digest overnight. Dithiothreitol (DTT) was added to a final concentration of 5 mM and reduced at 56 °C for 30 min, after which iodoacetamide (IAA) was added to a final concentration of 11 mM and incubated for 15 min at room temperature, protected from light. The peptides were solubilized by liquid chromatography in mobile phase A and then separated on a NanoElute Ultra-High-Performance Liquid Chromatography (UHPLC) System. Mobile phase A was an aqueous solution containing 0.1% formic acid and 2% acetonitrile; mobile phase B was a solution containing 0.1% formic acid and 100% acetonitrile. The liquid-phase gradient was set at 0–42 min, 6%–22% B; 42–54 min, 22%–32% B; 54–57 min, 32%–80% B; and 57–60 min, 80% B. The flow rate was maintained at 350.00 nL/min. The peptide fragments were separated by the UHPLC system and injected into the capillary Ion Source for ionization and then into the times-TOF Pro mass spectrometry for analysis. The ion source voltage was set at 2.0 kV, and the peptide parent ion and its secondary fragments were detected and analyzed using a high-resolution TOF. The scanning range of the secondary mass spectra was set to 100–1700, and the data were acquired in parallel accumulated serial fragmentation (PASEF) mode. One primary mass spectrum was acquired, followed by 10 secondary spectra in PASEF mode with parent ion charges in the range of 0–5, and the dynamic exclusion time of the tandem mass spectrometry scans was set to 30 s to avoid duplicate scans of the parent ions.

### Isolation, characterization, and intervention of hippocampal neuronal cells

The brain tissue was removed, and the bilateral hippocampus was obtained by isolation under a dissecting microscope, separated into small pieces with scissors and added with 0.125% trypsin, digested for 15 min in a cell culture incubator at 37 °C, and then added with 10% fetal bovine serum in DMEM/F12 culture medium to terminate the digestion and then beaten into a cell suspension, which was filtered through a 200-mesh sieve, and then re-suspended for 24 h in full volume and replaced with neurobasal culture medium. Neurobasal culture medium containing 2% B27, 100 U/mL penicillin, and 100 µg/mL streptomycin was replaced by half of the culture medium every 3 days, and the cells were cultured for 9 days. The neurons were identified by immunofluorescence staining to observe the expression level of the neuronal marker MAP2.

Hippocampal neuronal cells were treated as follows: 4 μM AGK2 (Sirt2-specific inhibitor) was added for 48 h; at the same time, 2.5 μM atorvastatin and 4 μM AGK2 were added for 48 h; lentivirus infection for 24 h or 48 h. Lentivirus infection was performed for 24 h or 48 h at a multiplicity of infection of 10, using viruses with the following titers: Sirt2 overexpression lentivirus at 3.2 × 10⁸ TU/mL, NFL overexpression lentivirus (NFL WT) at 2.8 × 10⁸ TU/mL, negative control lentivirus at 3.8 × 10⁸ TU/mL, NFL K272Q lentivirus at 2.6 × 10⁸ TU/mL, and NFL K272R lentivirus at 2.4 × 10⁸ TU/mL. As non-fluorescent lentiviruses were employed, transduction efficiency was validated through real-time PCR for quantitative gene expression analysis and Western blot for protein expression confirmation. Cells were collected for subsequent assays after treatment. To address potential off-target effects, we validated transduction efficiency through both real-time PCR and Western blot analyses. These verification methods allowed the detection of unintended molecular alterations while confirming target-specific expression. Regarding possible cellular functional abnormalities induced by gene overexpression, all lentiviral constructs were deliberately designed to contain only the coding sequence (CDS) region of target genes, excluding both 5′UTR and 3′UTR segments. This design strategy minimizes artifacts because non-coding regions can unpredictably alter exogenous mRNA stability and protein translation processes, thereby reducing risks of non-physiological cellular responses. Based on the information of the rat Sirt2 gene, overexpression lentivirus and negative control empty lentivirus were constructed with an HA tag; rat hippocampal neuron cells were taken, and rat Sirt2 overexpression lentivirus (HA-Sirt2) or negative control lentivirus was infected into the cells. Based on the information of the rat NFL gene, overexpression lentivirus and negative control airborne lentivirus were constructed with a Flag tag; that is, rat NFL wild-type expression lentivirus (NFL WT). Based on the information of rat NFL gene, mutant lentivirus 1 (NFL K272Q) and mutant lentivirus 2 (NFL K272R) were constructed with Flag tags, and the same negative control airborne lentivirus of NFL WT was used as a control, rat hippocampal neuronal cells were taken, and the constructed lentiviruses were grouped according to the grouping, with non-transduced neurons and empty vector controls included in all experiments to monitor potential off-target effects. The cells were infected and routinely cultured for 48 h for subsequent assays. Based on the information of the rat NFL gene, we constructed its interfering lentivirus (Lv-shNFL) and its control lentivirus (Lv-shNC); rat hippocampal neuron cells were taken, and the constructed lentiviruses were infected into cells according to the grouping, and routinely cultured for 48 h for the subsequent tests.

### Immunofluorescence

Tissue paraffin sections (5 μm), dewaxed to water and placed in boiling antigen repair solution, low-fire repair for 10 min, rinsed with PBS, goat serum was added dropwise until the tissue was completely covered, and incubated at room temperature for 15 min in a wet box; P-TauS202/T205 and NFL primary antibody were added dropwise, and incubated at 4 °C overnight in a wet box; FITC-labeled goat anti-mouse IgG and Cy3-labeled fluorescent secondary antibody were added dropwise. Goat anti-mouse IgG and cy3-labeled rabbit IgG fluorescent secondary antibody were added and incubated in the wet box at room temperature for 60 min; the nuclei were rinsed and wiped with PBS, DAPI was added dropwise to re-stain the nuclei, and an anti-fluorescence quencher was added dropwise to clean the nuclei with PBS, the coverslips were sealed, and the staining effect of P-TauS202/T205 and NFL in hippocampus was observed under the fluorescence microscope at 400×.

### Western blot

The total protein in the sample was extracted, and the protein was quantified using the BCA protein concentration determination kit. 40 μg of protein was sampled, subjected to SDS-PAGE electrophoresis, and transferred to the PVDF membrane, which was blocked with 5% skimmed milk powder for 1 h. The sample was put into the NFL, GH1, Sirt2, and Flag primary antibody working solution for overnight incubation at 4 °C, and then washed with TBST buffer to clean the PVDF membrane, and then put into the IgG-HRP secondary antibody working solution for incubation at 37 °C for 45 min. The PVDF membrane was washed with TBST buffer and incubated in IgG-HRP secondary antibody working solution at 37 °C for 45 min, and the ECL substrate was luminescent. β-actin was used as an internal reference. The optical density values of the target bands were analyzed using a gel image processing system (Gel-Pro-Analyzer software).

### Real-time PCR

Total RNA was extracted from the samples, and cDNA was obtained under the action of Super M-MLV reverse transcriptase. The real-time PCR reaction system was constructed according to the instructions of the SYBR green kit, and the constructed PCR reaction system was put into the fluorescence quantification instrument for fluorescence quantification. And β-actin was used as an internal reference to analyze the quantification of mRNA by using the 2^−ΔΔCT^ method.

### Cellular thermal shift assay

Hippocampal neuronal cells were treated with either 2.5 μM atorvastatin or DMSO for 1 h. The harvested cells were then lysed using a lysis buffer containing 10% phosphatase inhibitors. Subsequently, the lysates were centrifuged at 16,000 × *g* at 4 °C for 10 min to separate the soluble proteins from cellular debris. The supernatant was divided into eight equal aliquots, each of which was subjected to heating at different temperatures (46, 50, 54, 58, 62, 66, 70, and 74 °C) for 3 min using a PCR machine. Following the heating process, the samples were centrifuged again at 16,000 × *g* at 4 °C for 20 min. The resulting supernatants were collected for subsequent Western blot analysis.

### Molecular docking

Molecular docking was performed using CB-Dock2 to conduct blind docking of atorvastatin with SIRT2 to identify potential druggable binding pockets. This web server is equipped with a robust computational algorithm for cavity detection based on curvature, which guides the docking process. Initially, the 3D structure of atorvastatin in SDF format was obtained from PubChem, and the 3D structure of the SIRT2 protein in PDB format was retrieved from AlphaFold. Subsequently, the 3D structures of atorvastatin and SIRT2 were input into CB-Dock2 for molecular docking analysis.

### Co-Immunoprecipitation and mass spectrometry

Total protein of the samples was extracted, and anti-Flag antibody, NFL, or IgG was added and incubated at 4 °C overnight; then 60 μl Protein A agarose beads were added and incubated at 4 °C for 2 h; then the agarose beads-antigen-antibody complexes were collected by centrifugation, and the immunoprecipitation complexes were separated by SDS-PAGE electrophoresis after heating at 96 °C for 10 min. Subsequently, the expression levels of Huwe1, NFL, Ubiquitin, PanKcr, and Sirt2 in the immunoprecipitated complexes were detected by Western blot, respectively; after staining with Caulobacter Brilliant Blue, the protein bands were excised from the gel and analyzed by mass spectrometry. For Ubiquitin detection, cells were pretreated with 20 μM MG-132 for 4 h before total protein extraction.

### LC-MS analysis and bioinformatics methods

Protein extraction and trypsinization were performed as detailed in the section “Results.” LC-MS instrumentation parameters and data acquisition settings (including mobile phases, gradient program, ionization voltage, and PASEF mode configuration) are identical to those described in the section “Results” and therefore not repeated here. For bioinformatics analysis, raw MS data were processed using MaxQuant (v1.6.6.0). Differentially expressed proteins were annotated into three Gene Ontology (GO) categories: biological processes, cellular components, and molecular functions. Statistical significance of enrichment was determined by Fisher’s exact test with a *P* < 0.05 threshold.

### Quantification and statistical analysis

Statistical analysis is described in each figure legend. Data were analyzed using Prism software (GraphPad 8.0). Data are expressed as the mean ± SEM for normally distributed data. Differences between the groups in the behavioral experiments were analyzed by repeated-measure ANOVA. One-way ANOVA was used for multiple group comparisons. *p* values < 0.05 were considered significant, <0.01 very significant, and <0.001 highly significant.

## Supplementary information

original WB

## Data Availability

Requests for data collected for the study can be made to the corresponding authors and will be considered on reasonable request.
