# Supplementary material for: Long-term atorvastatin improves cognitive function by modulating SIRT2-mediated dynamic transition of NFL lysine 272 crotonylation to ubiquitination in naturally aging rats
Source: Cell Death Discov. 2025 Oct 16;11:463. doi: 10.1038/s41420-025-02764-7 (PMC12533114; doi:10.1038/s41420-025-02764-7)

figure 3A—NFL

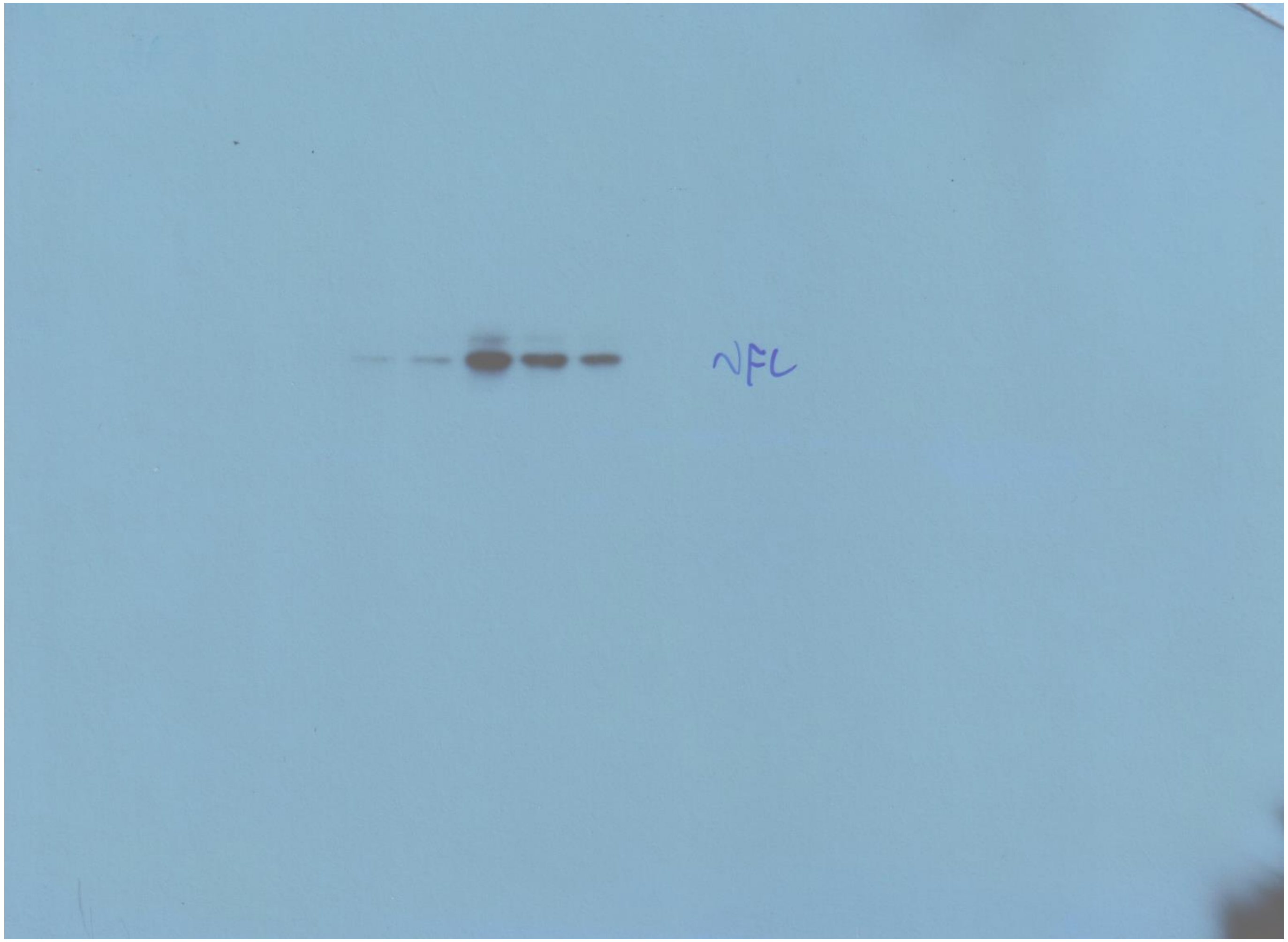

figure 3A—β-actin

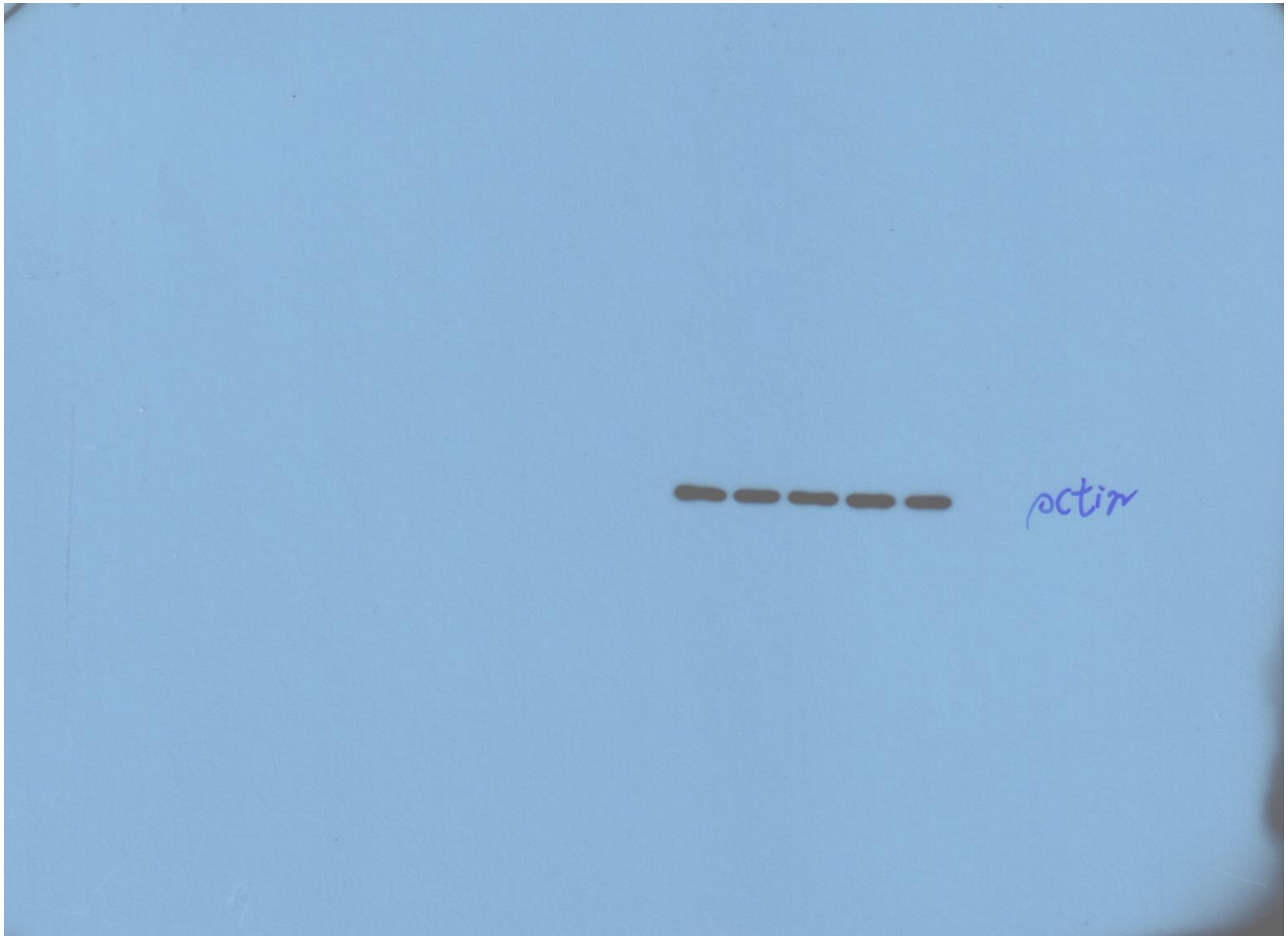

figure 4B—Ubiquitin

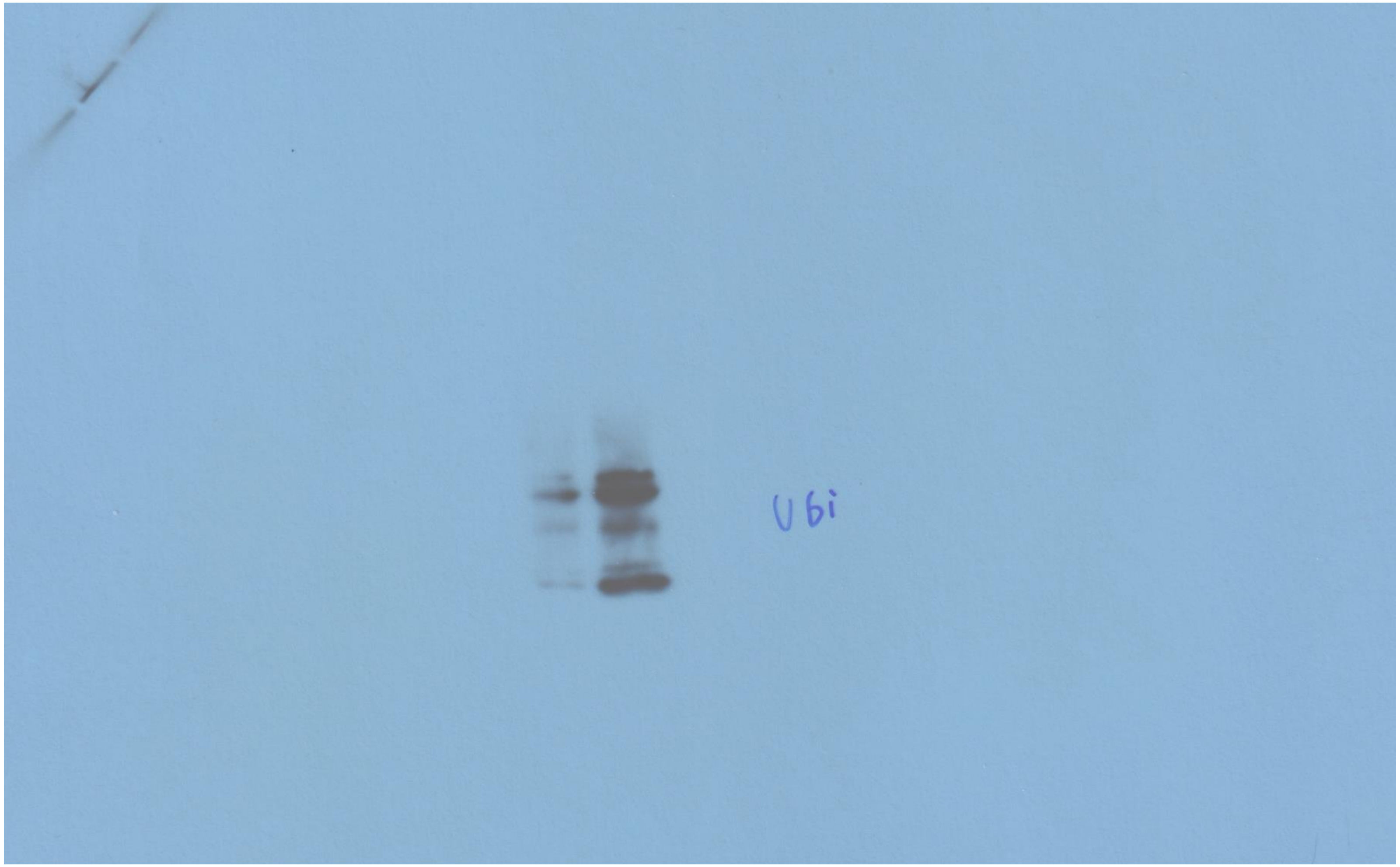

figure 4B—NFL

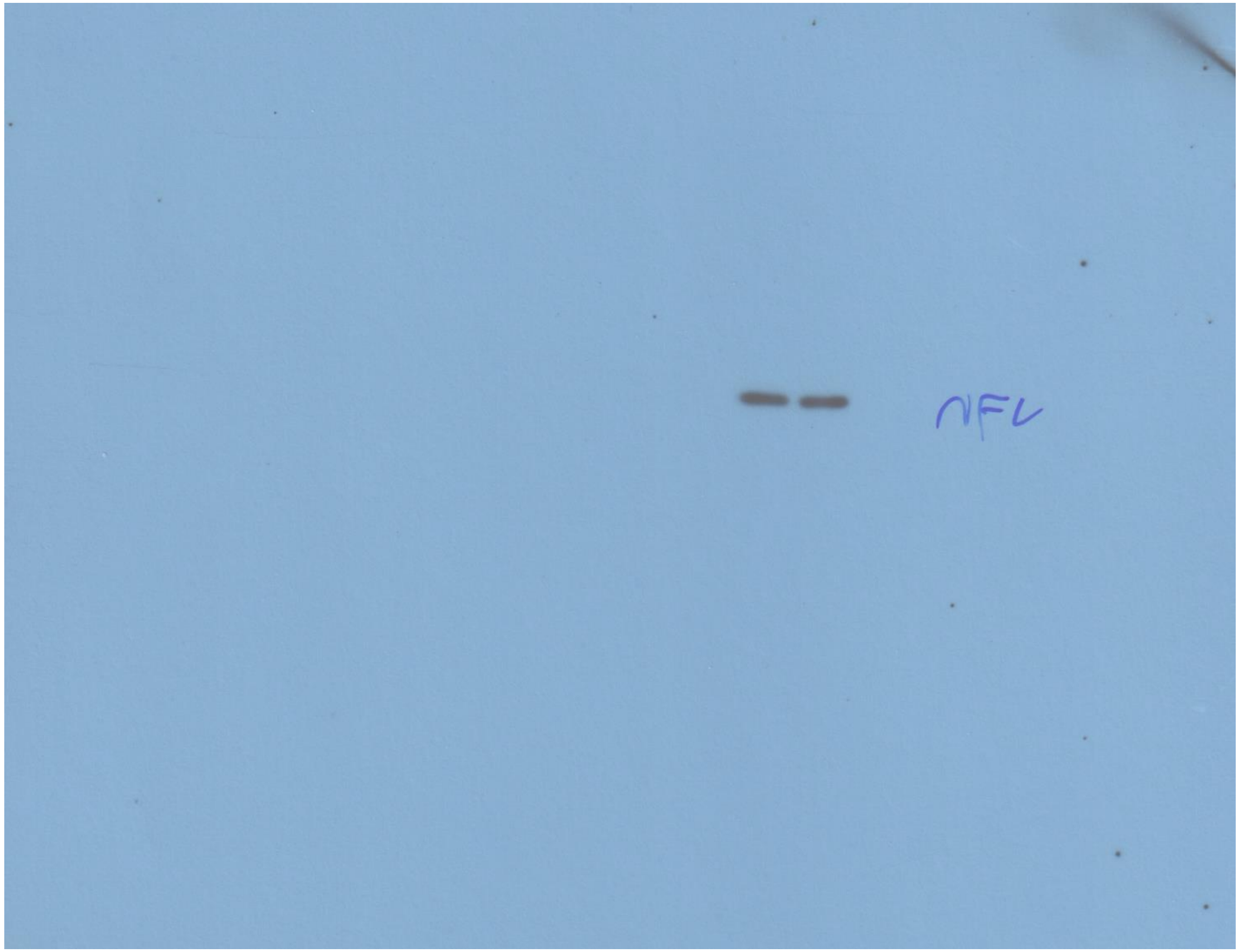

figure 4C—Pankcr

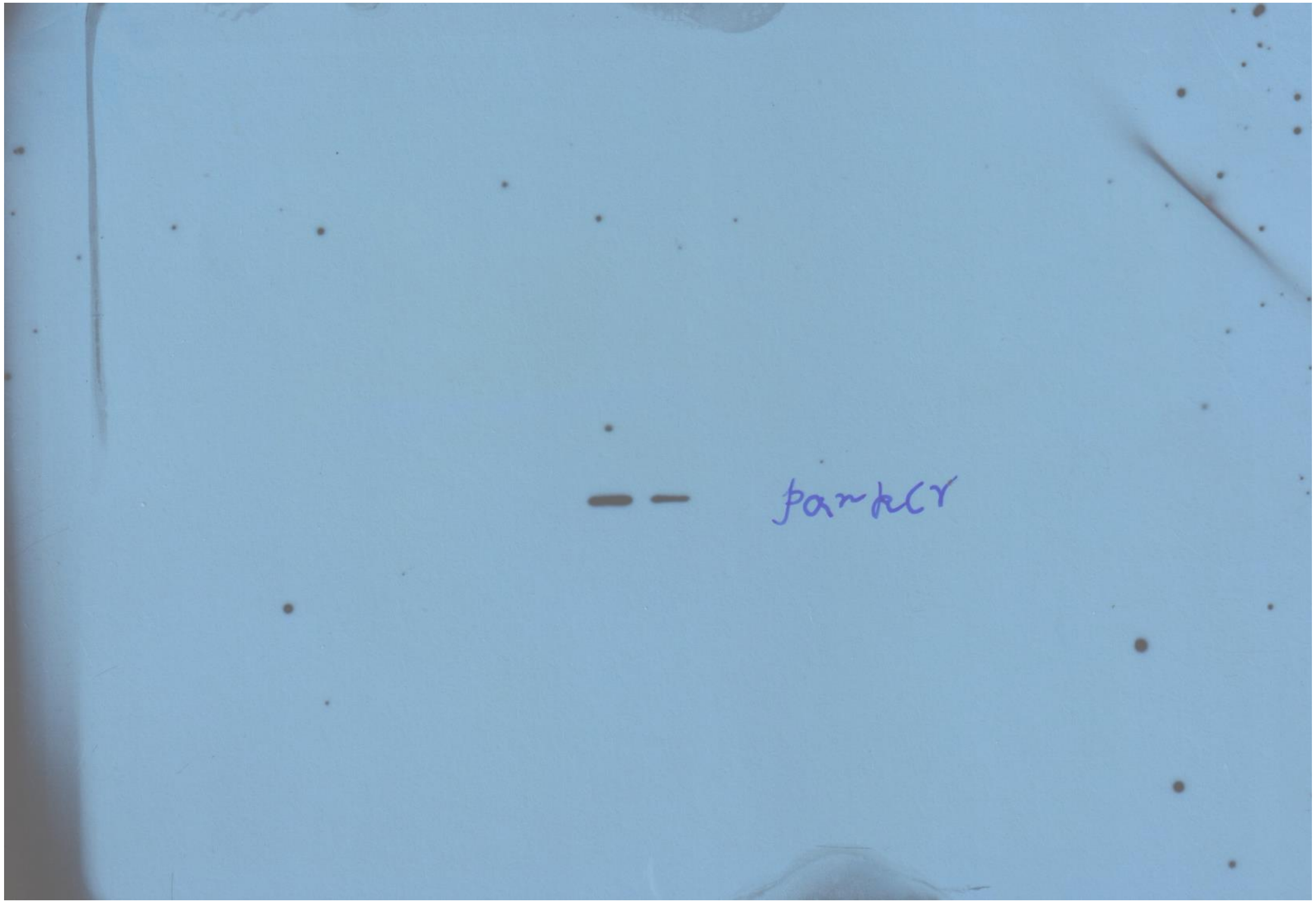

figure 4C—NFL

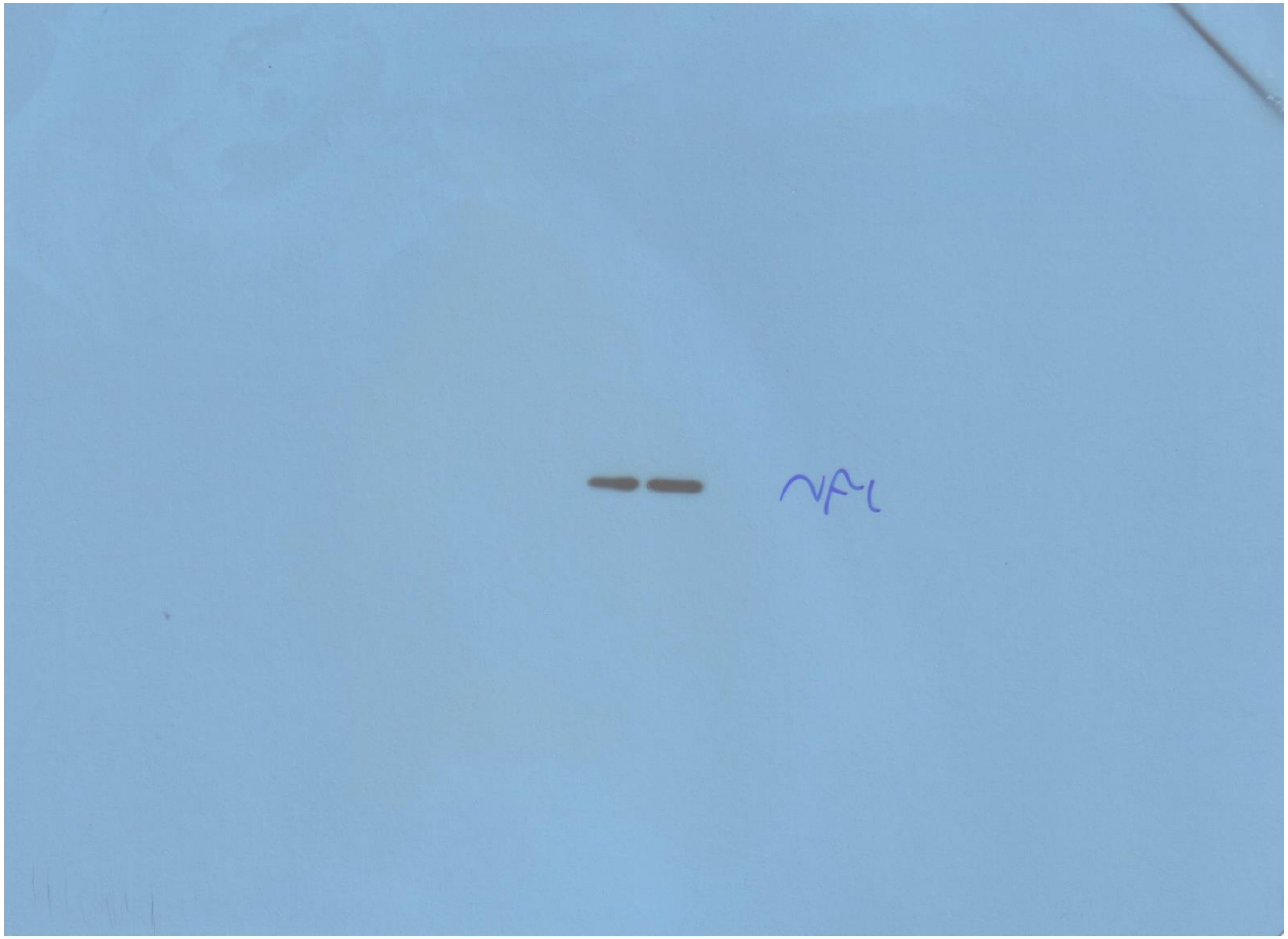

figure 4E—NFL

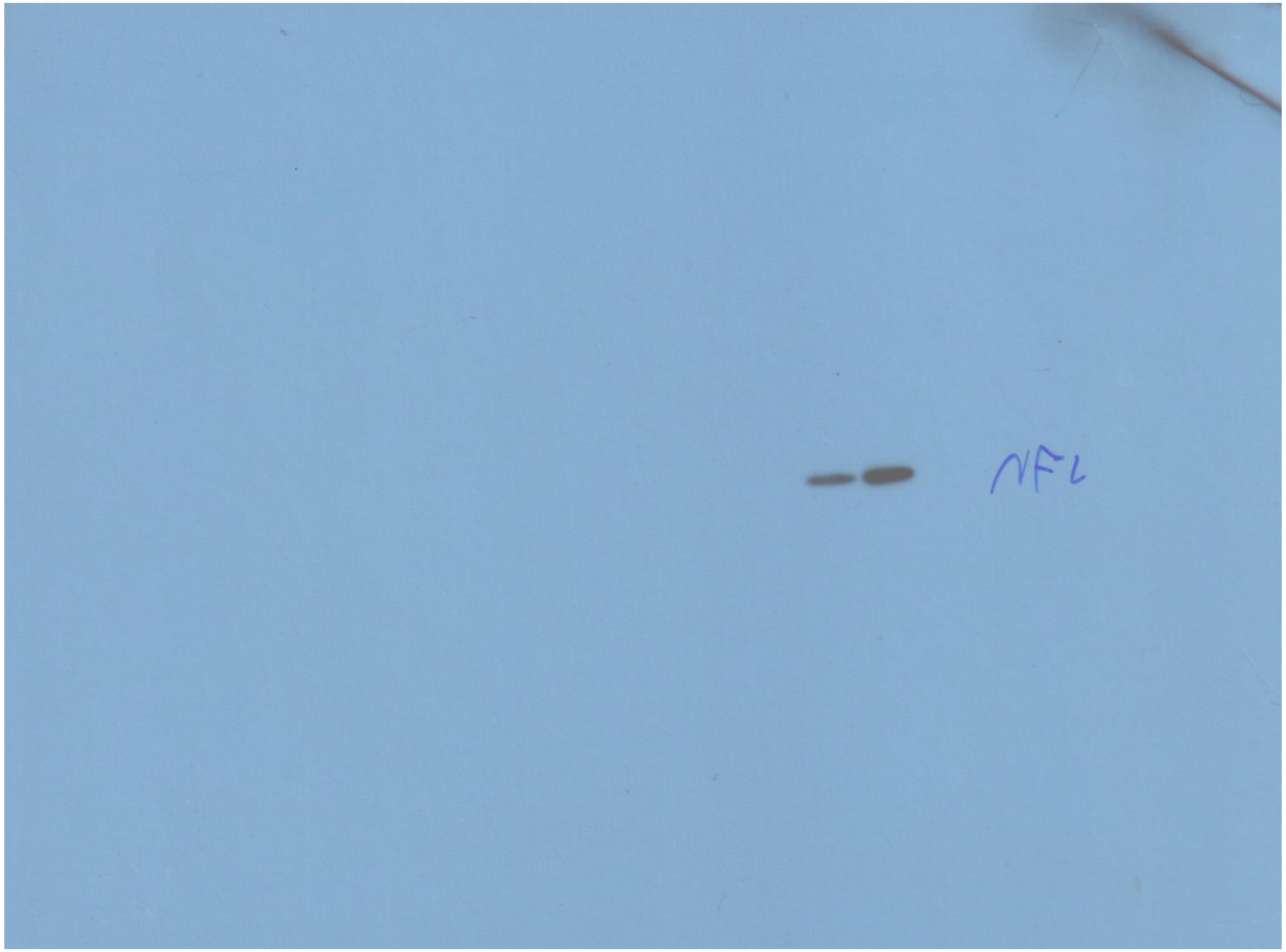

figure 4E—SIRT2

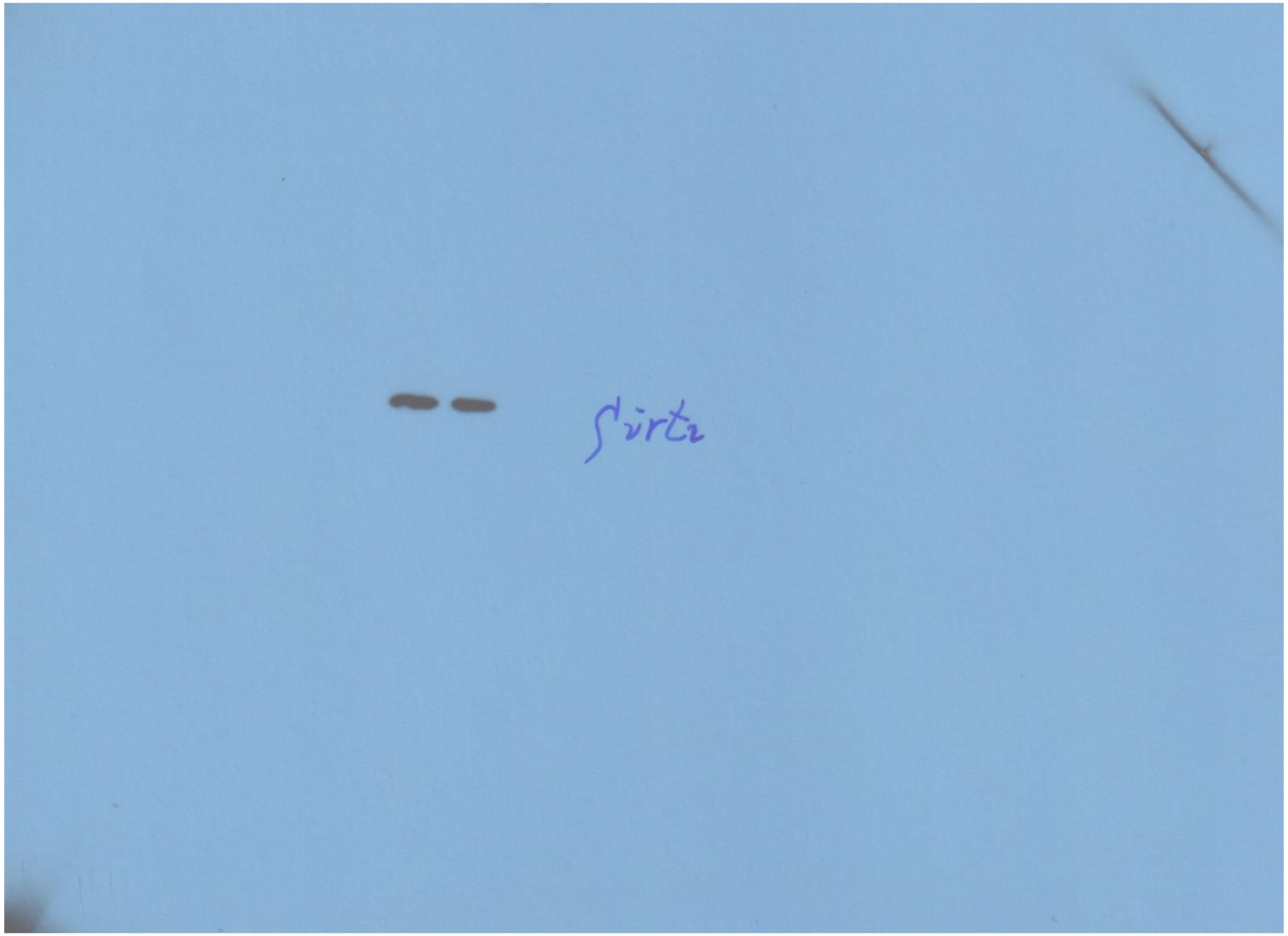

figure 4F—NFL

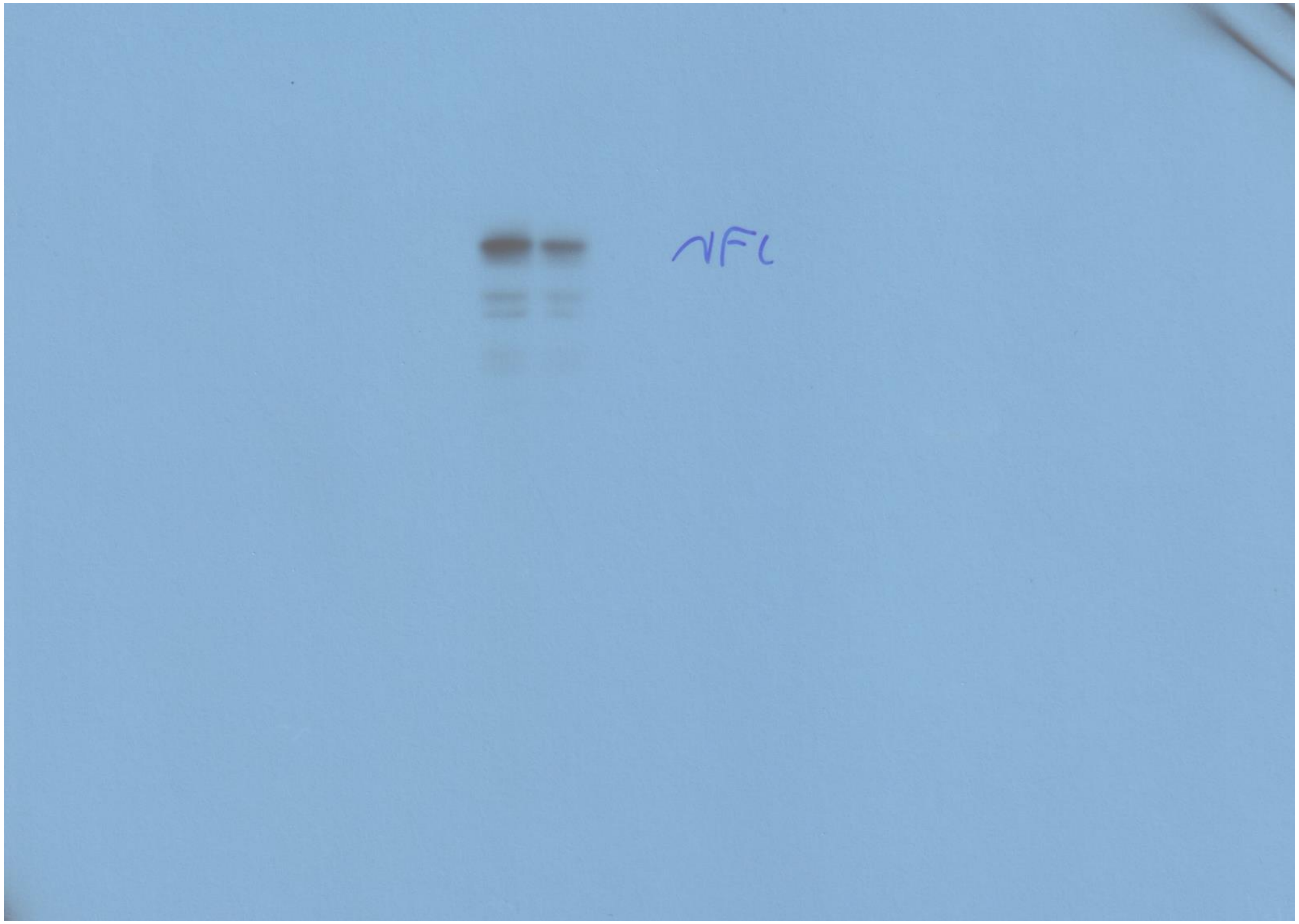

figure 4F—HUWE1

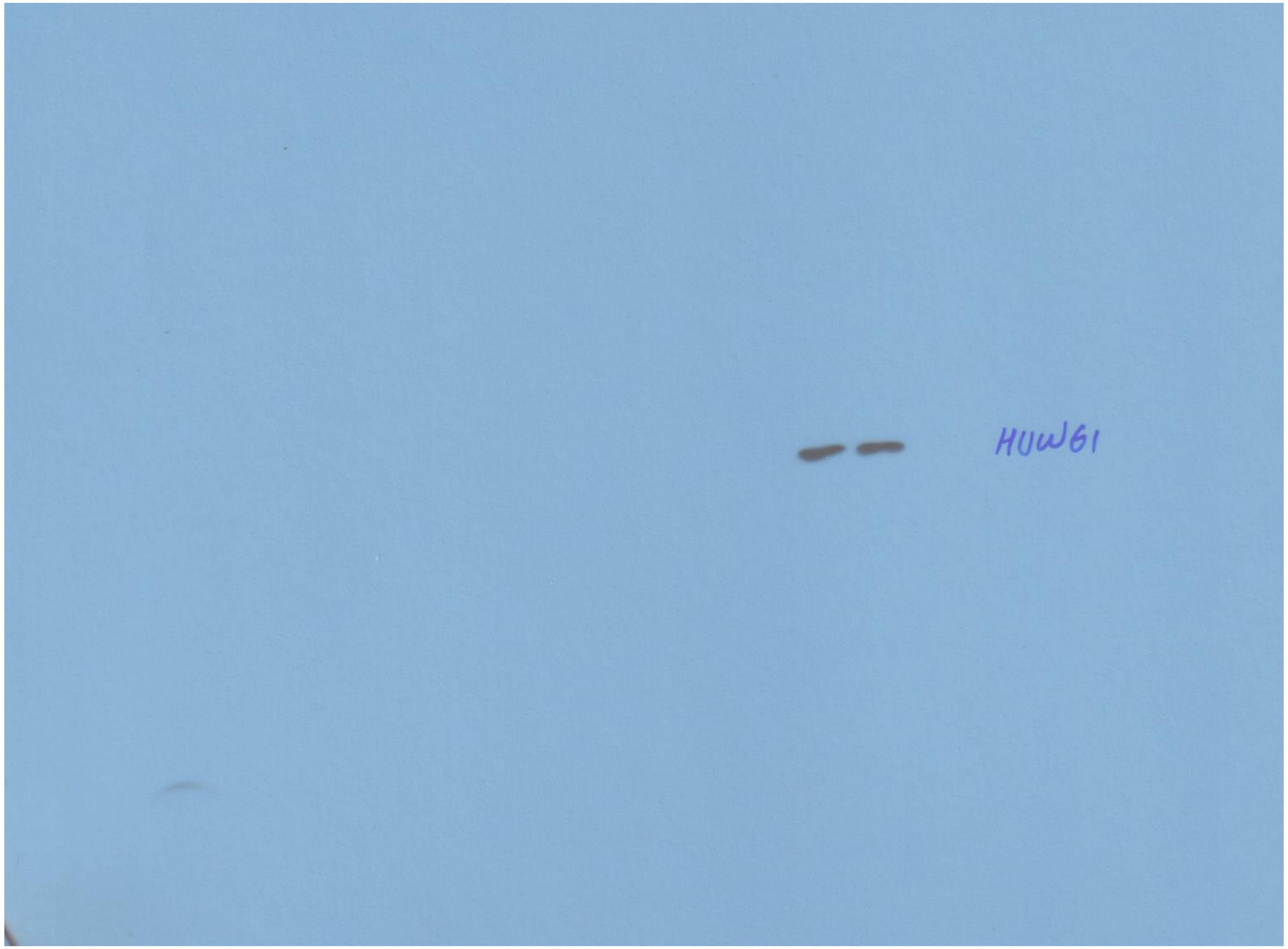

figure 5B—NFL

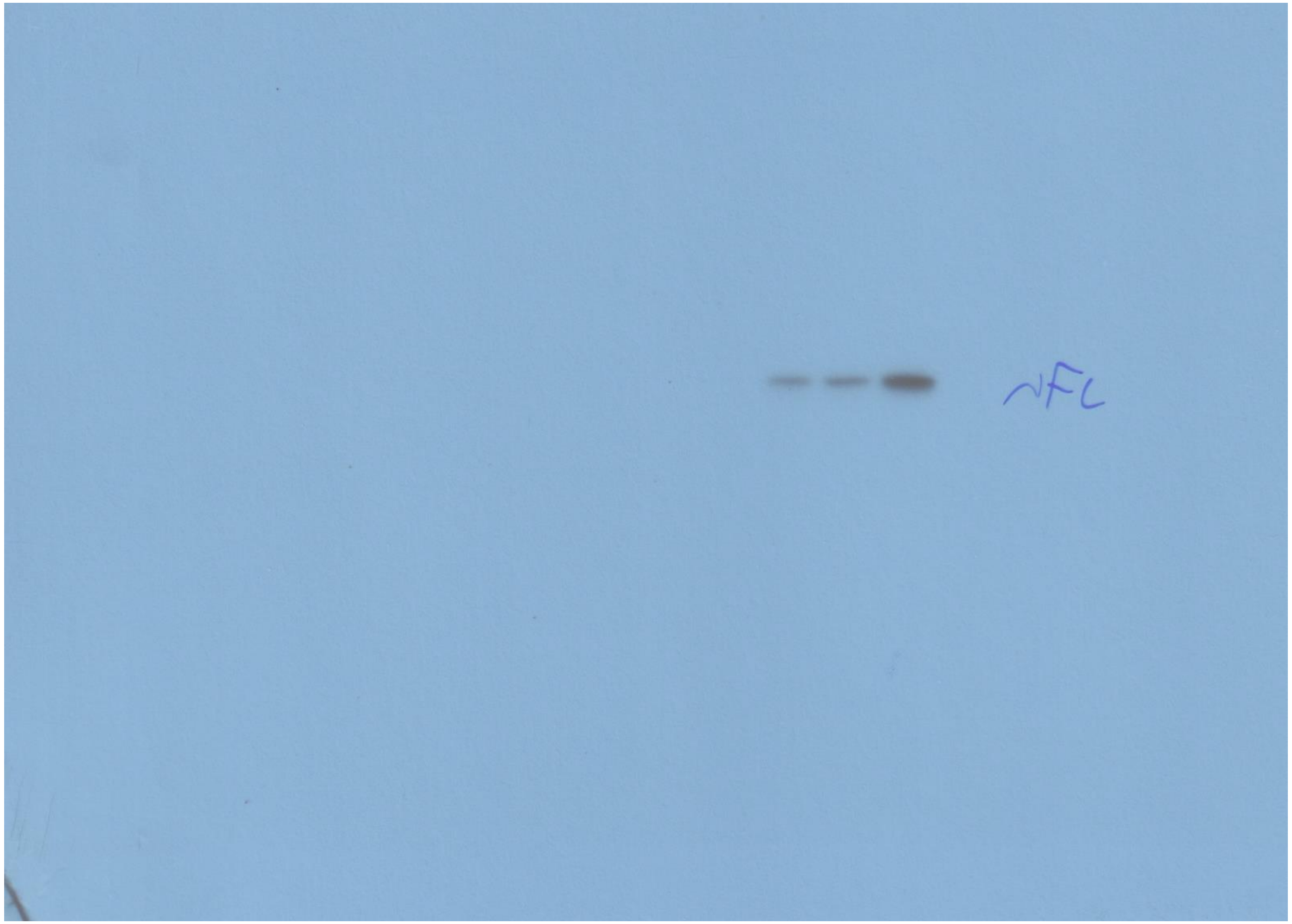

figure 5B—β-actin

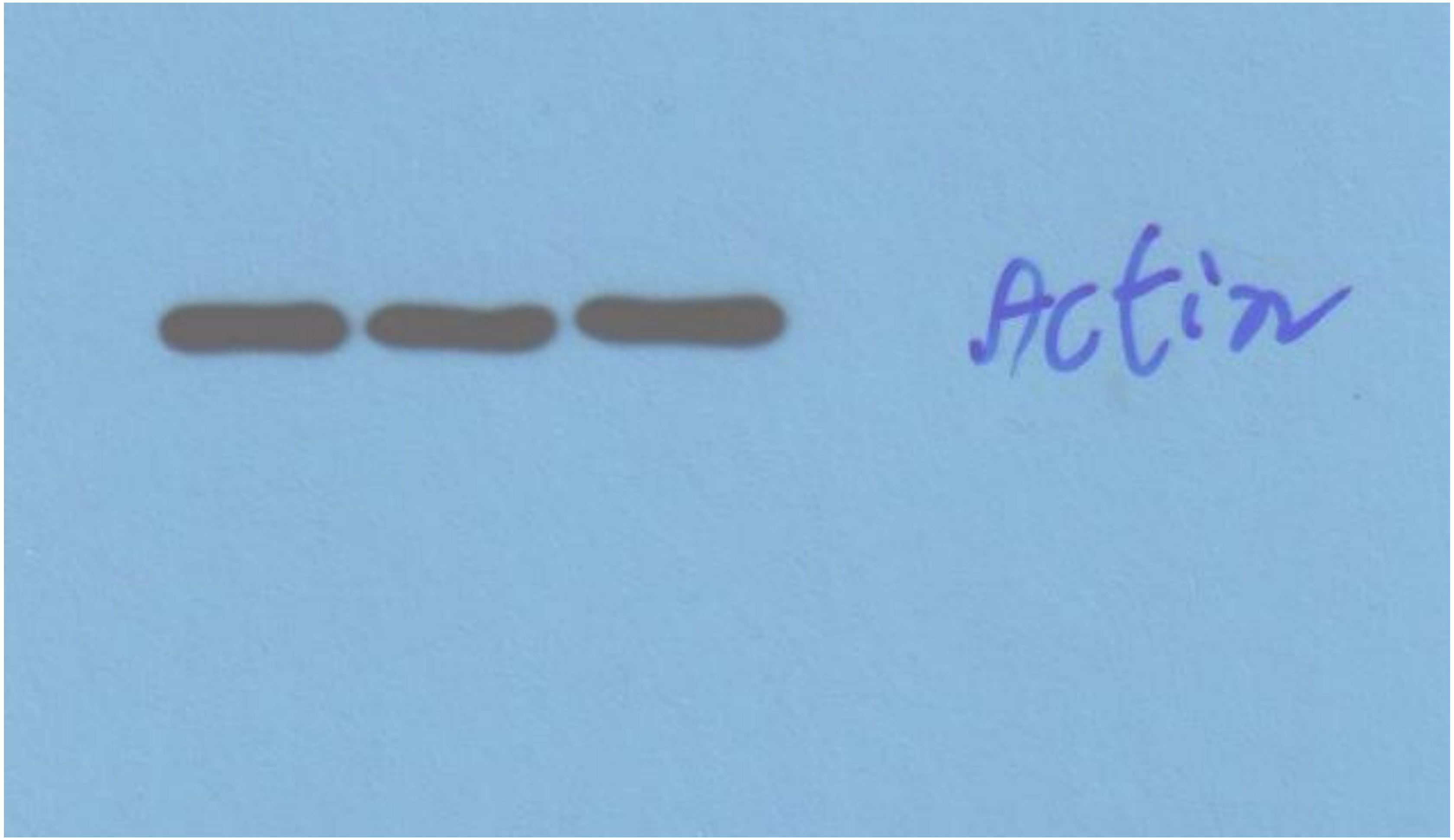

figure 5B—SIRT2

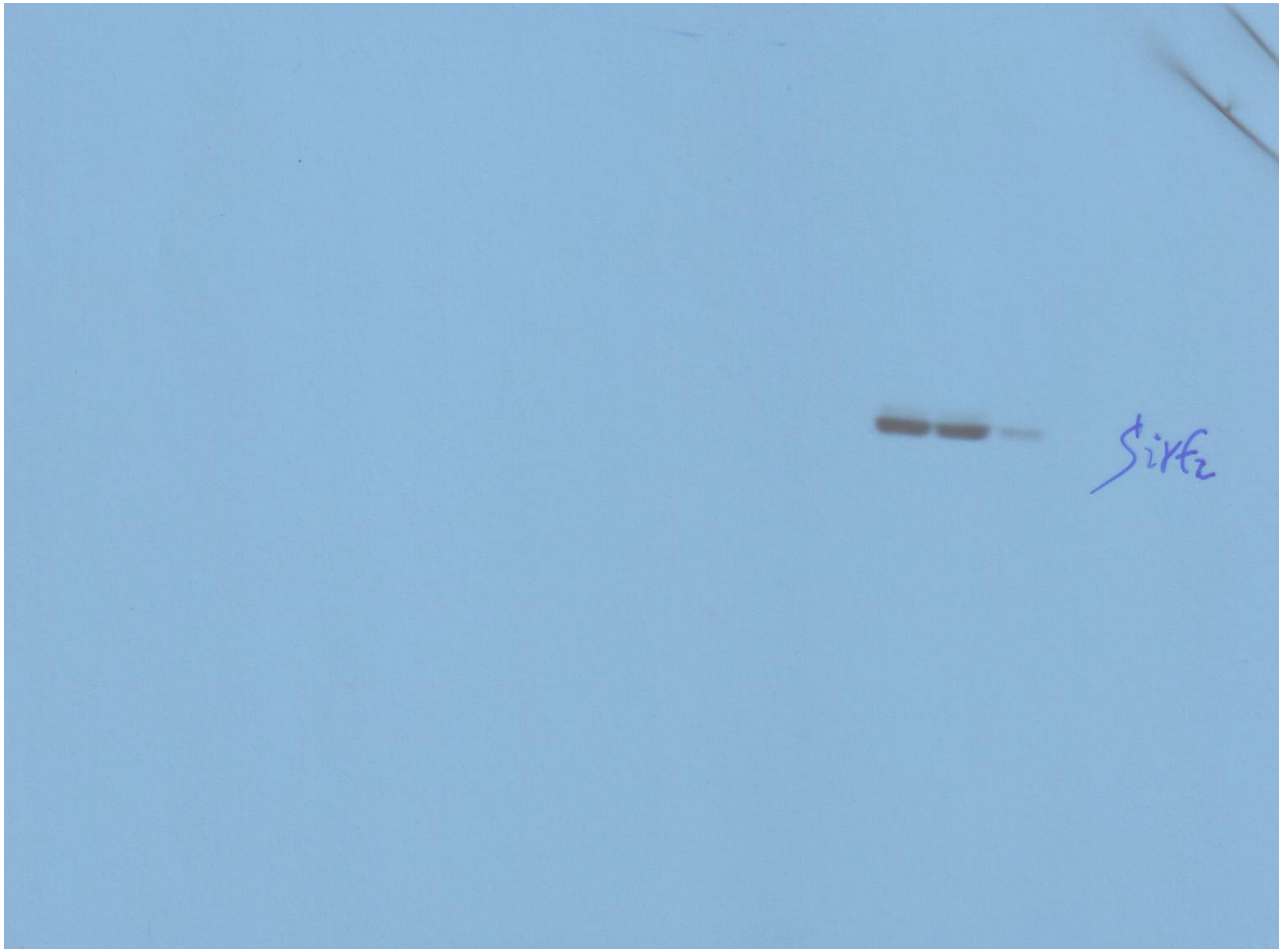

figure 5B—β-actin

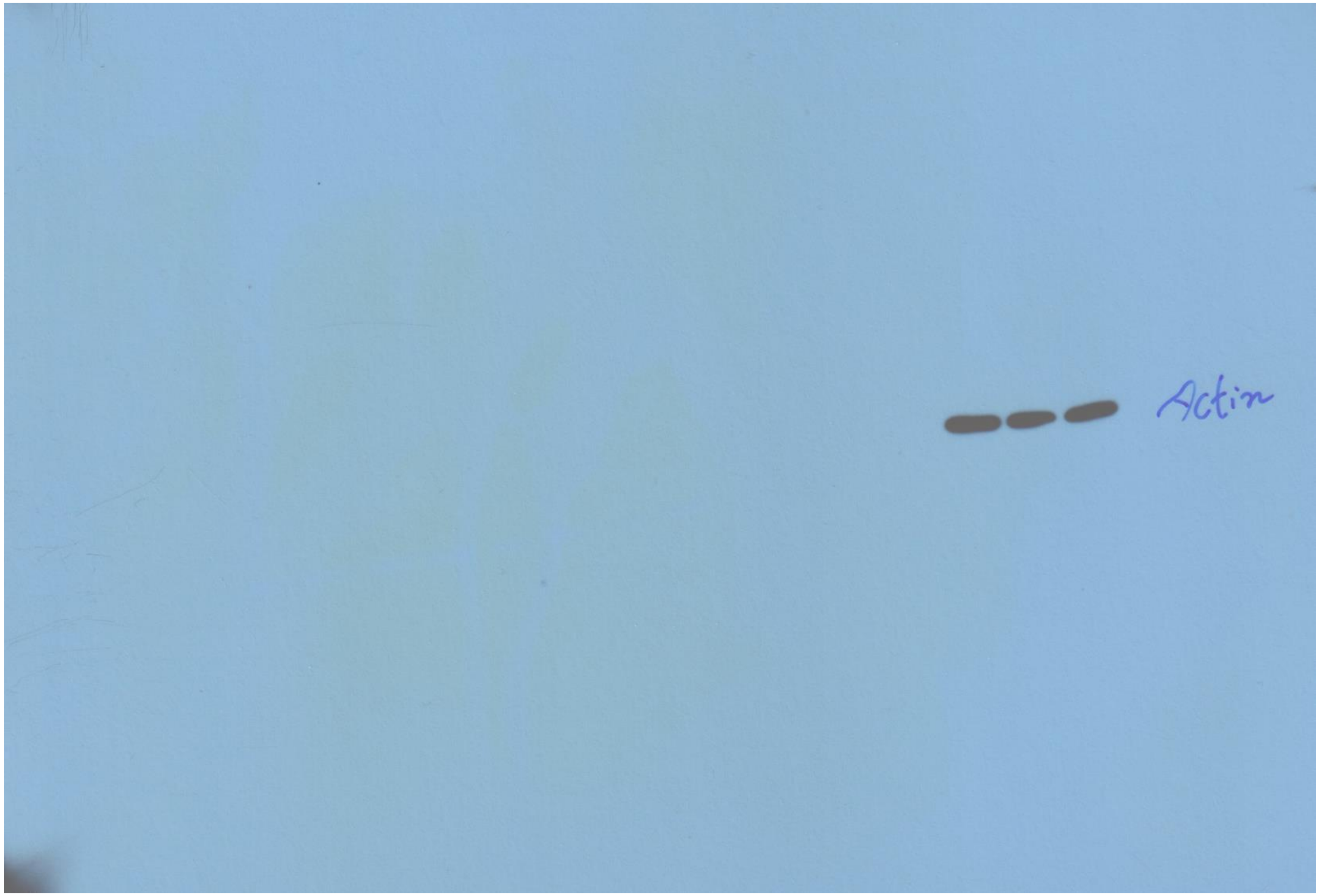

figure 5C—Ubiquitin

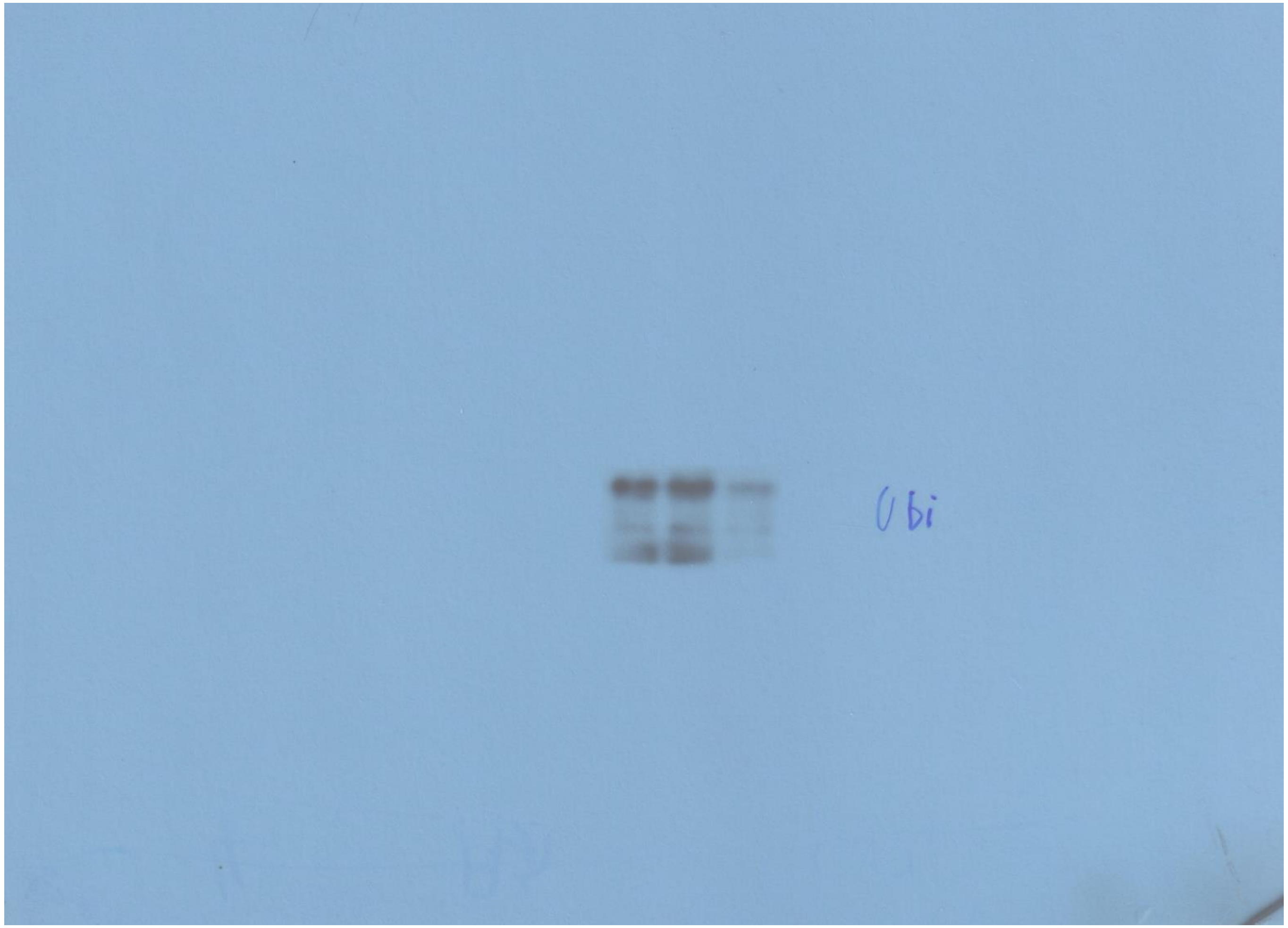

figure 5C—NFL

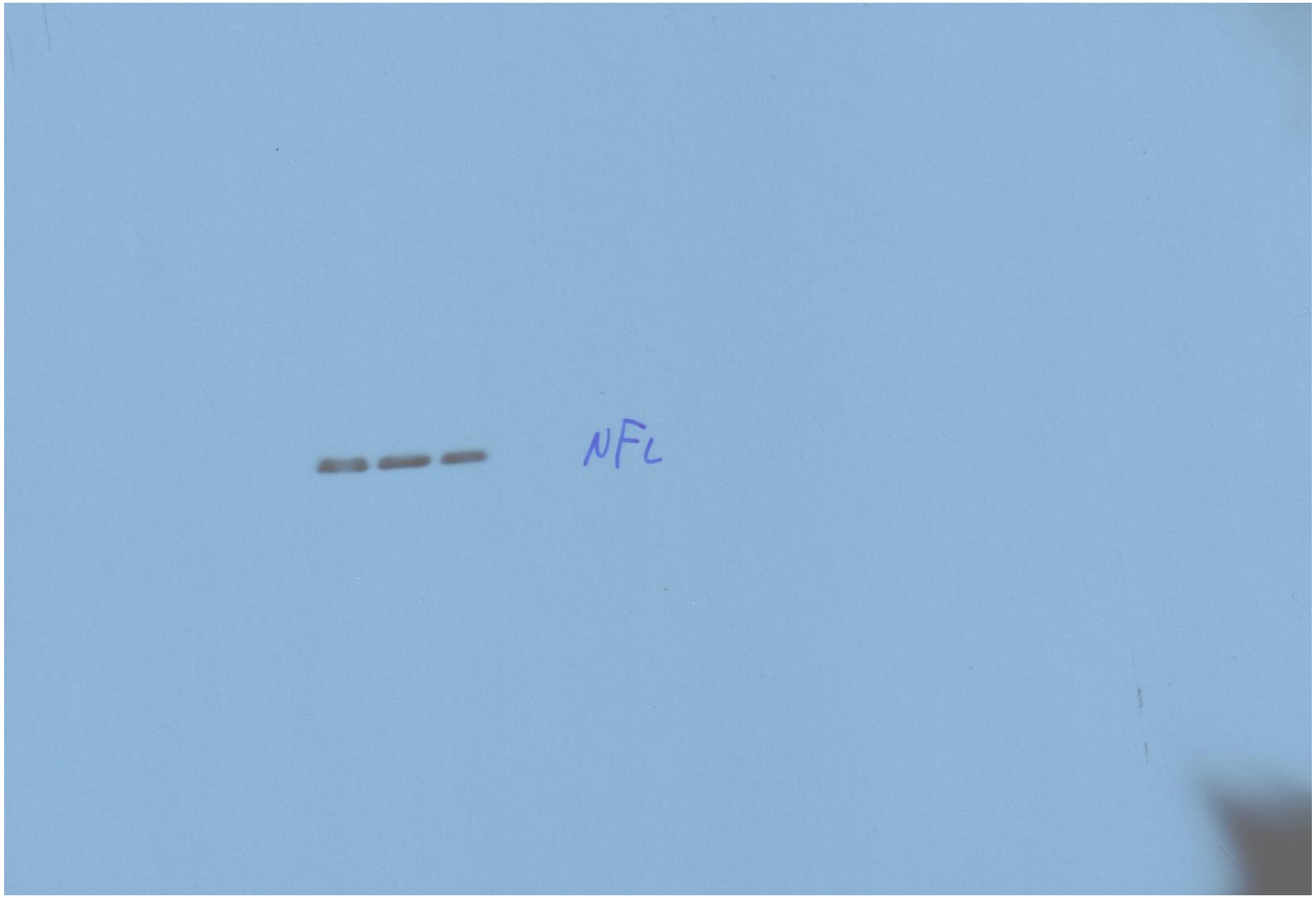

figure 5D—Pankcr

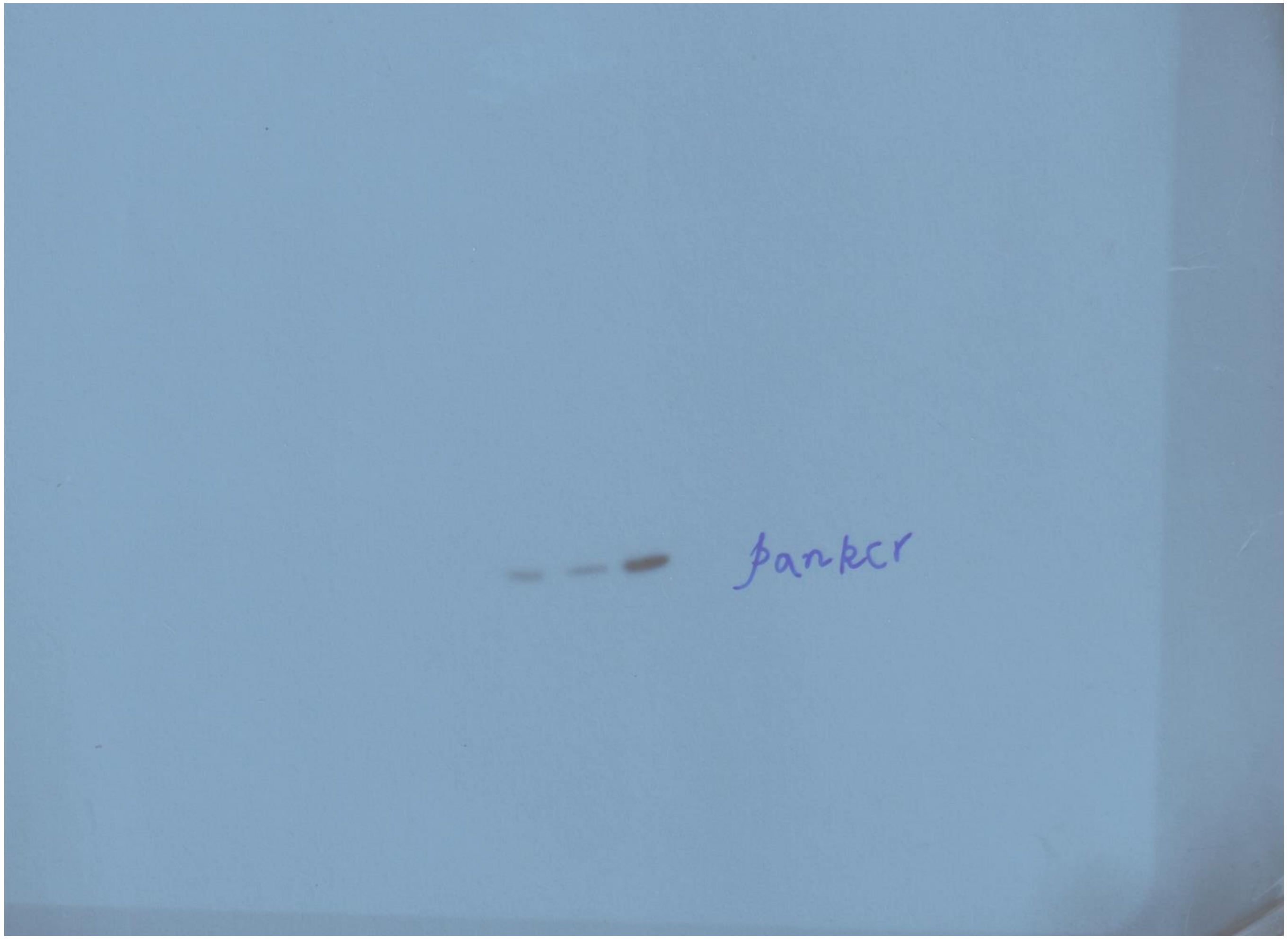

figure 5D—NFL

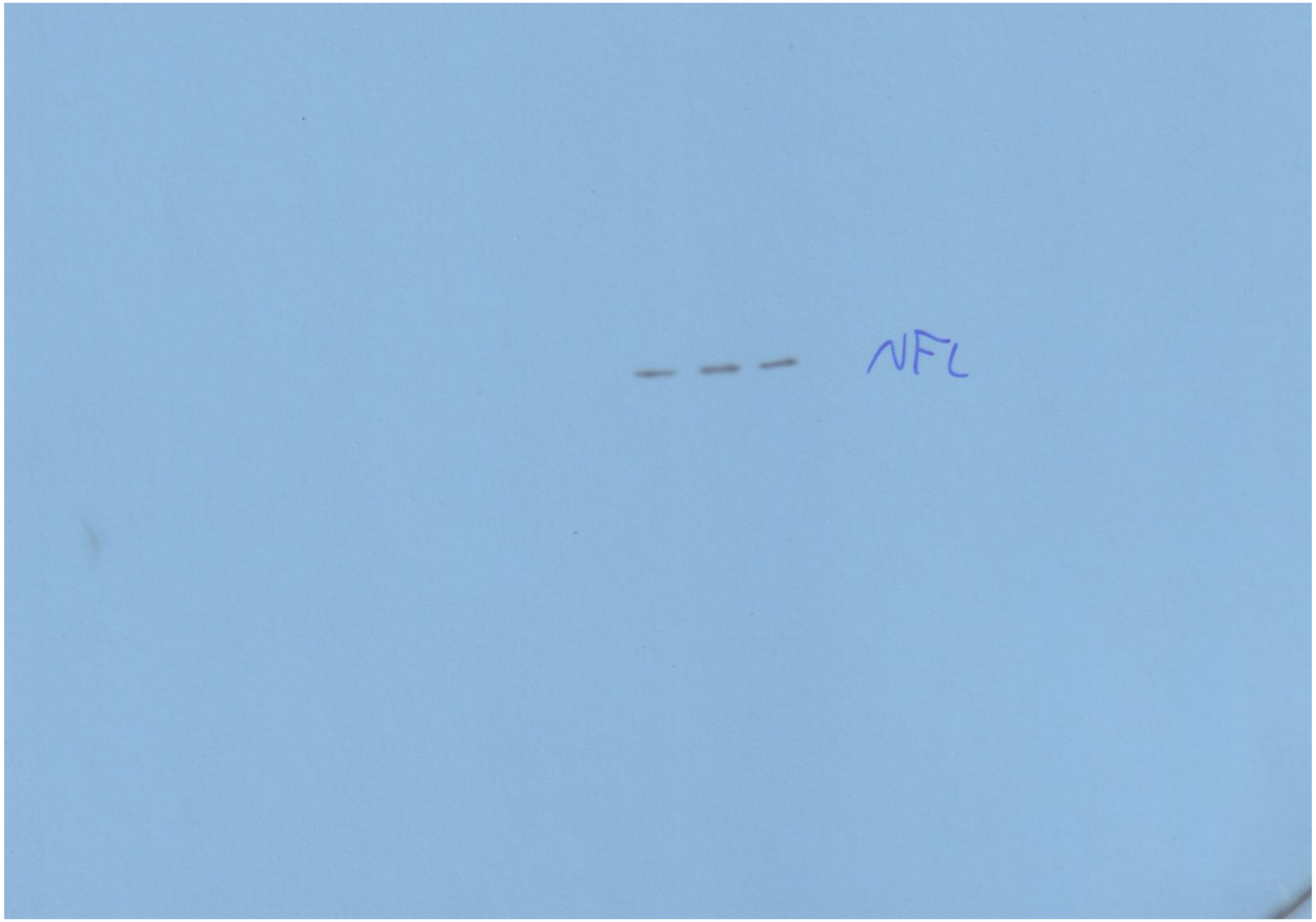

figure 5E—SIRT2

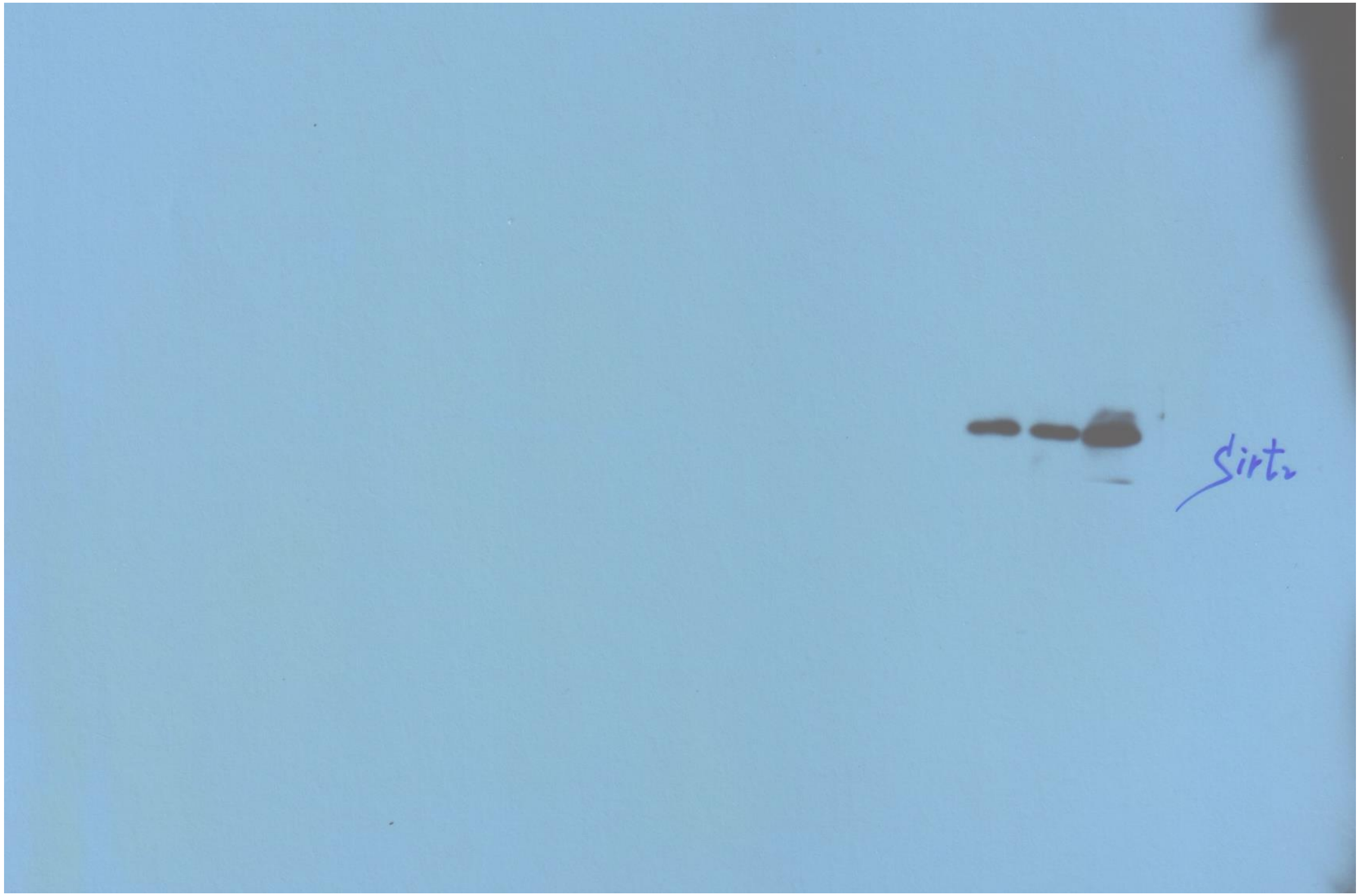

figure 5E—β-actin

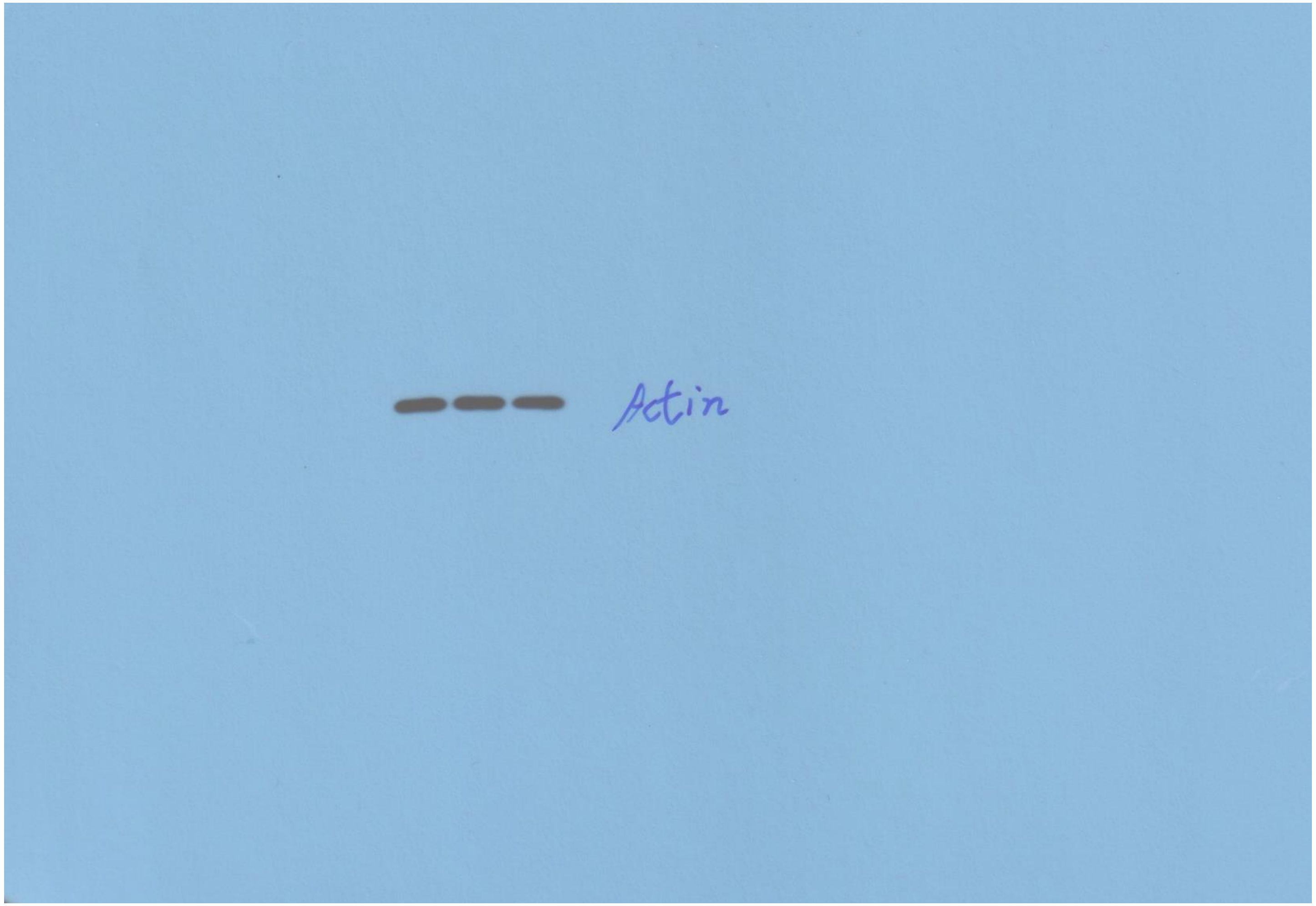

figure 5F—NFL

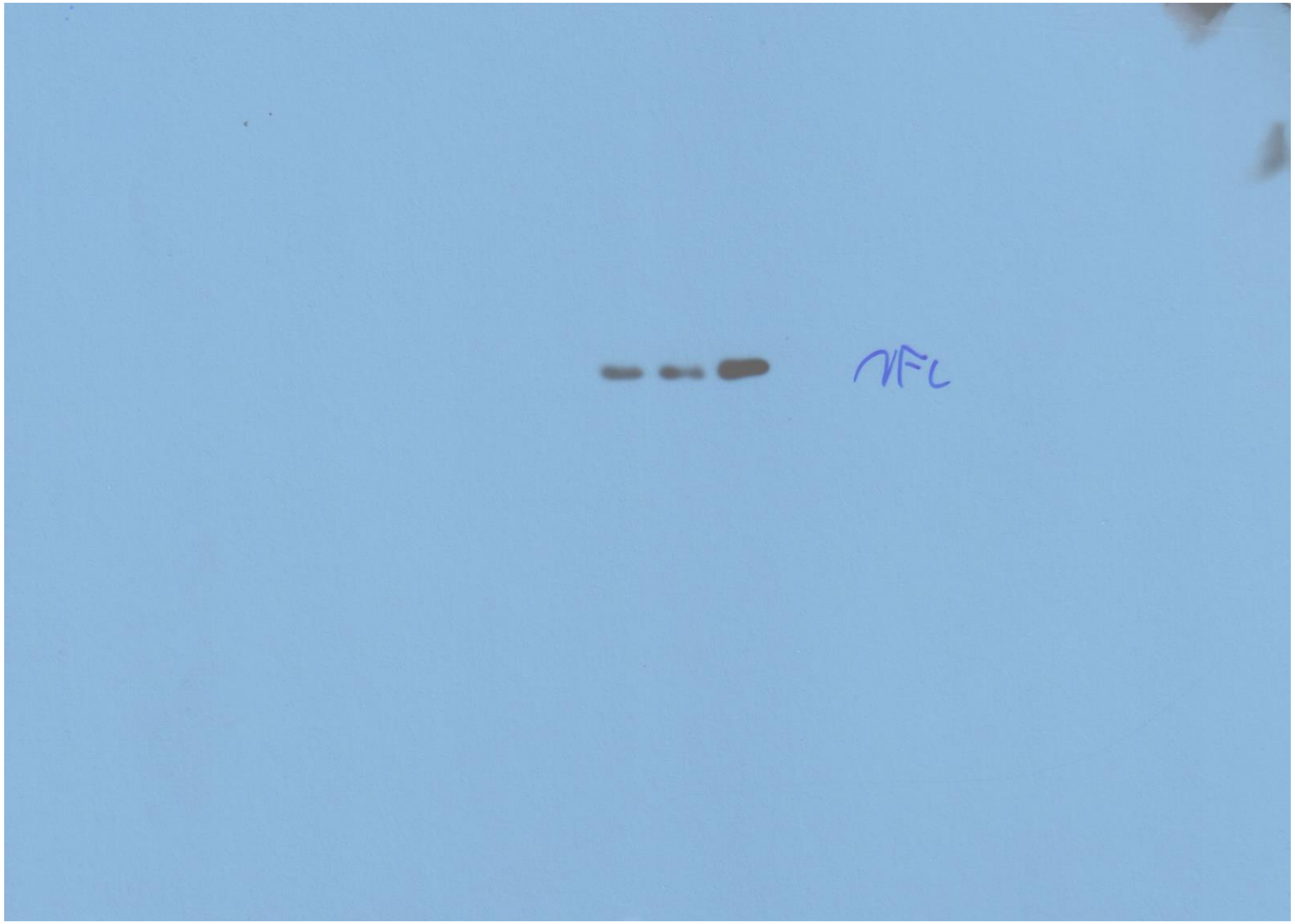

figure 5F—β-actin

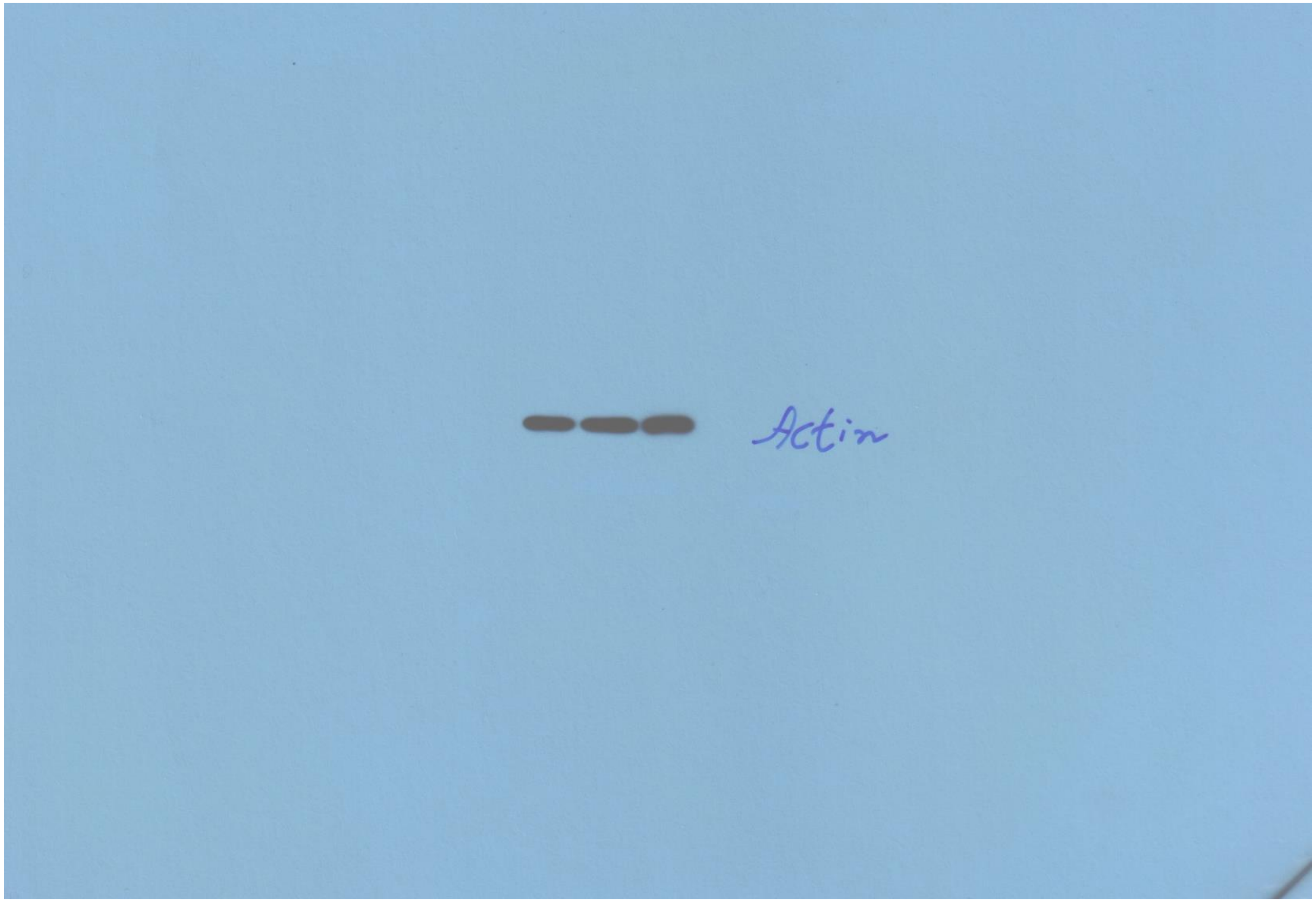

figure 5G—Pankcr

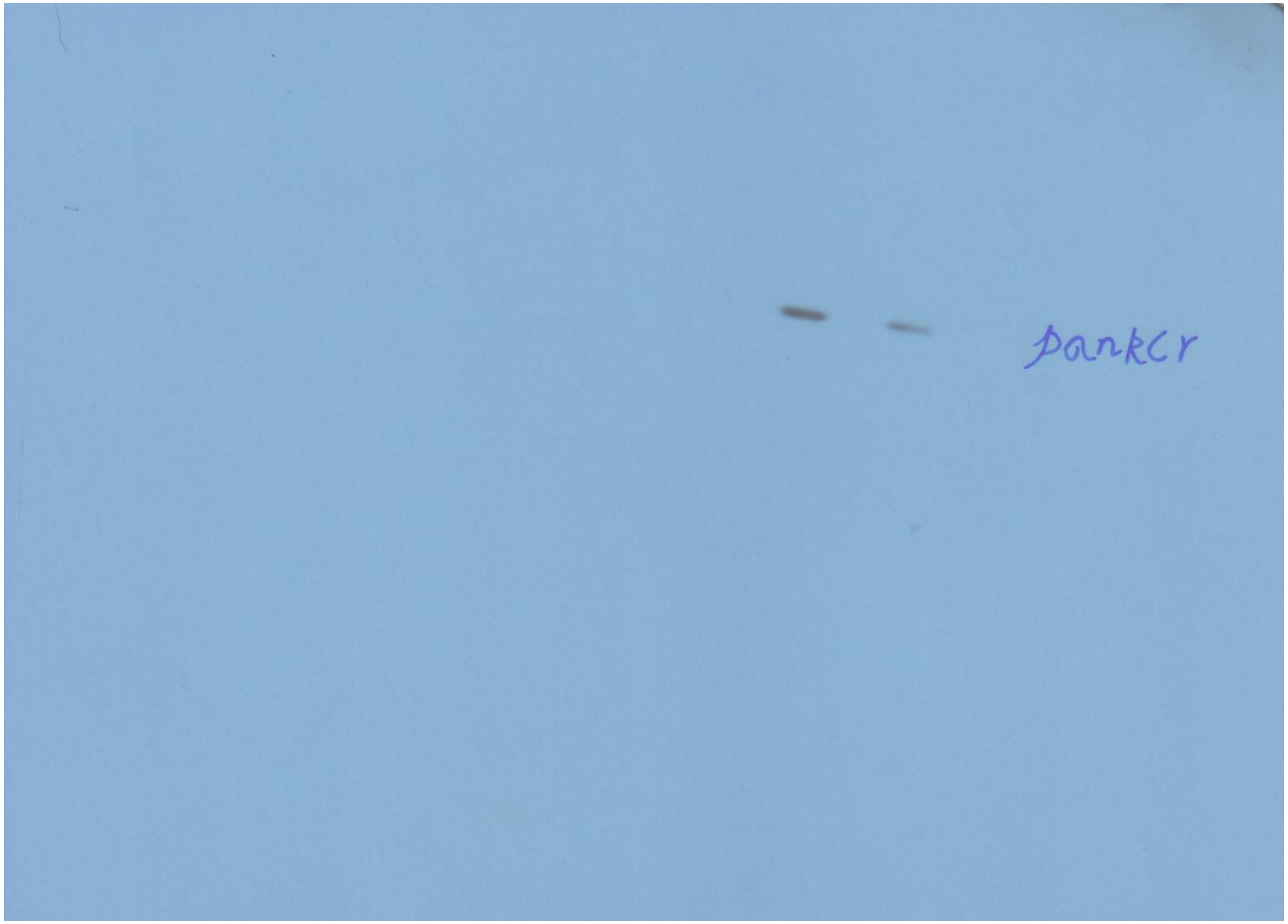

figure 5G—Flag

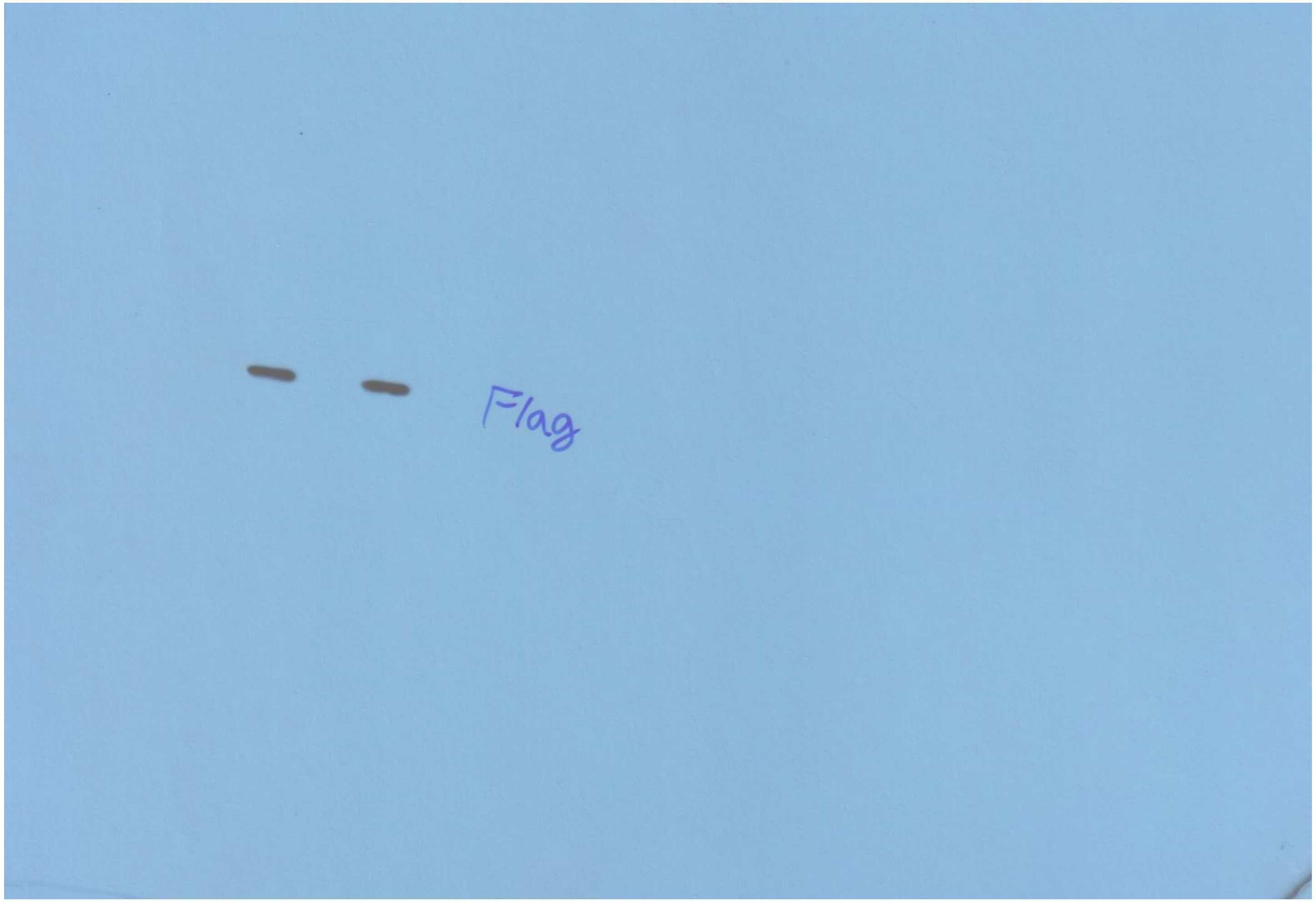

figure 5H—Ubiquitin

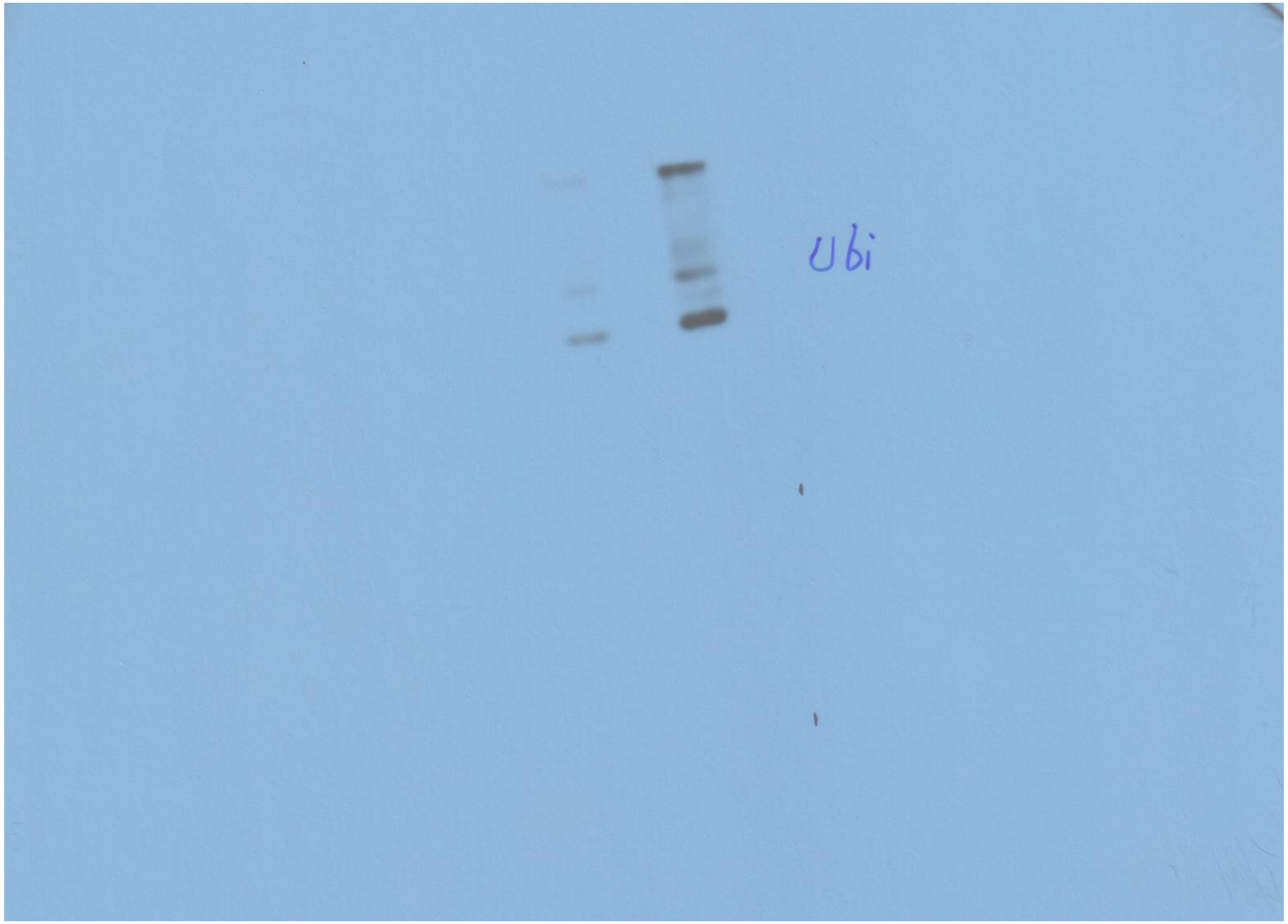

figure 5H—Flag

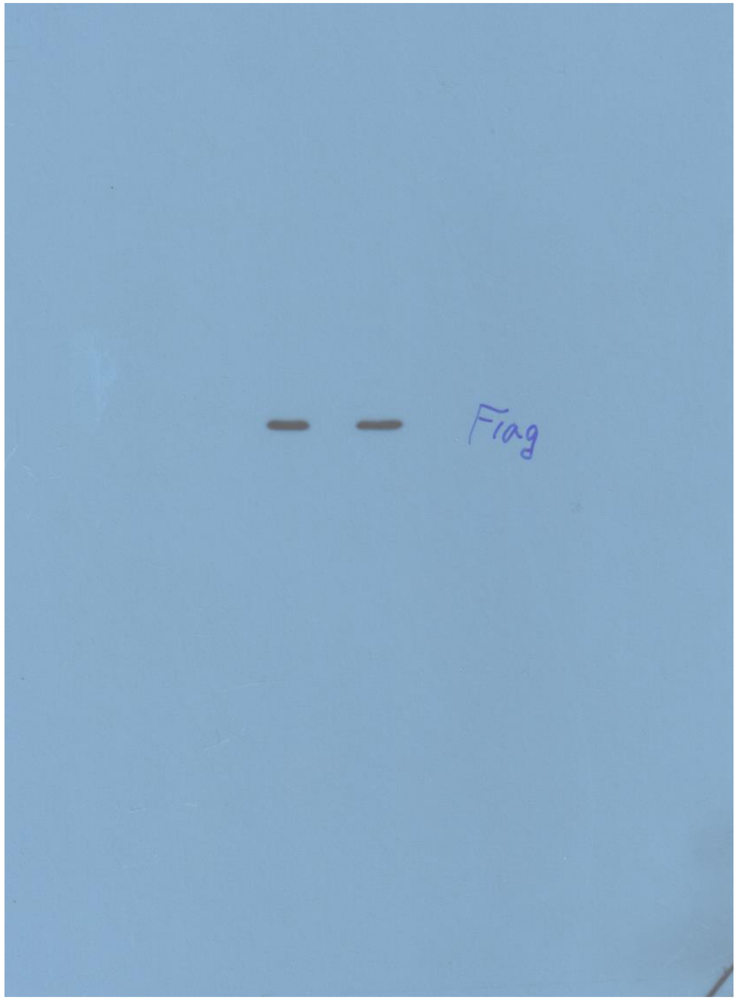

figure 6B—Statin

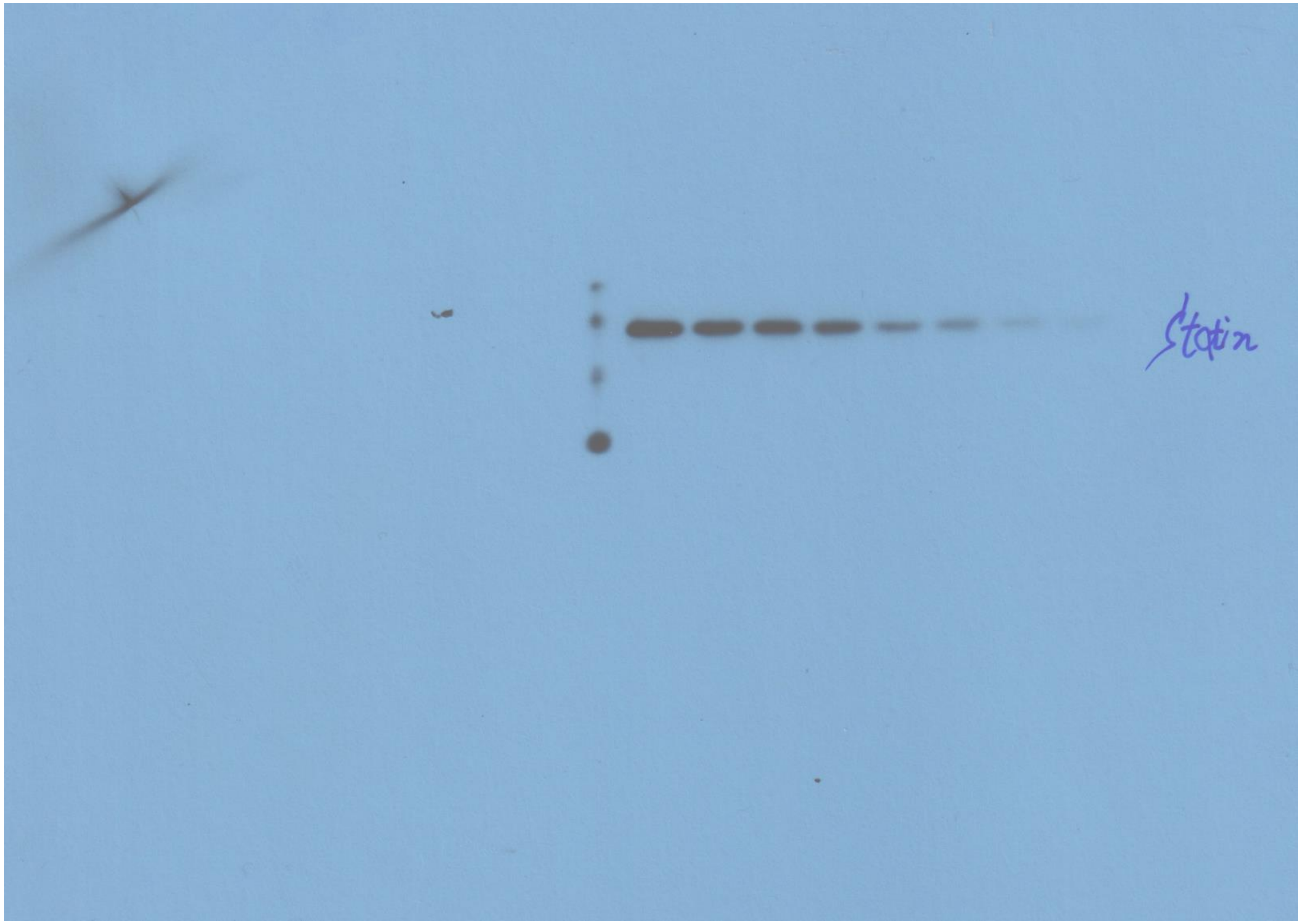

figure 6B—DMSO

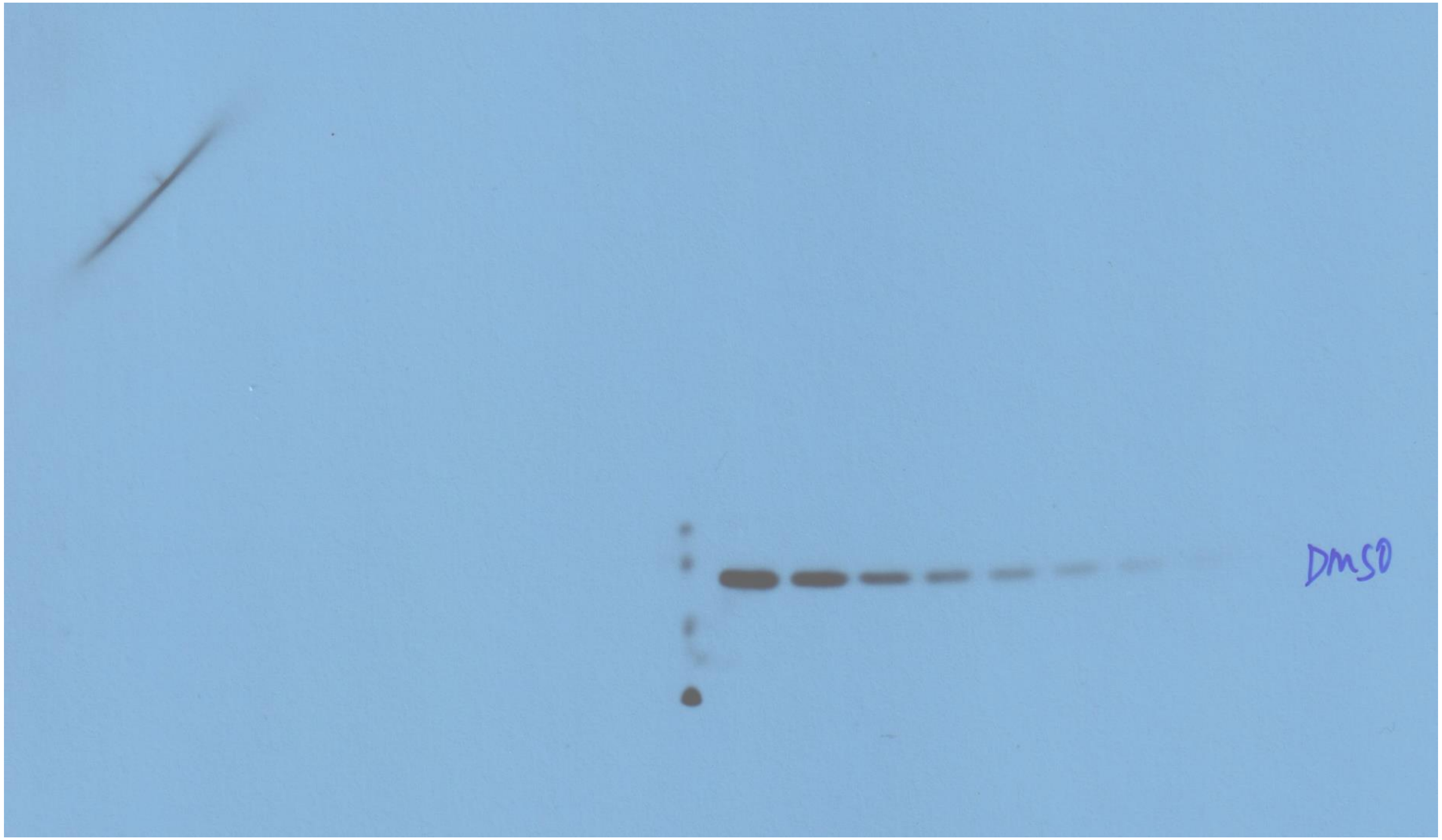

figure 6C—Pankcr

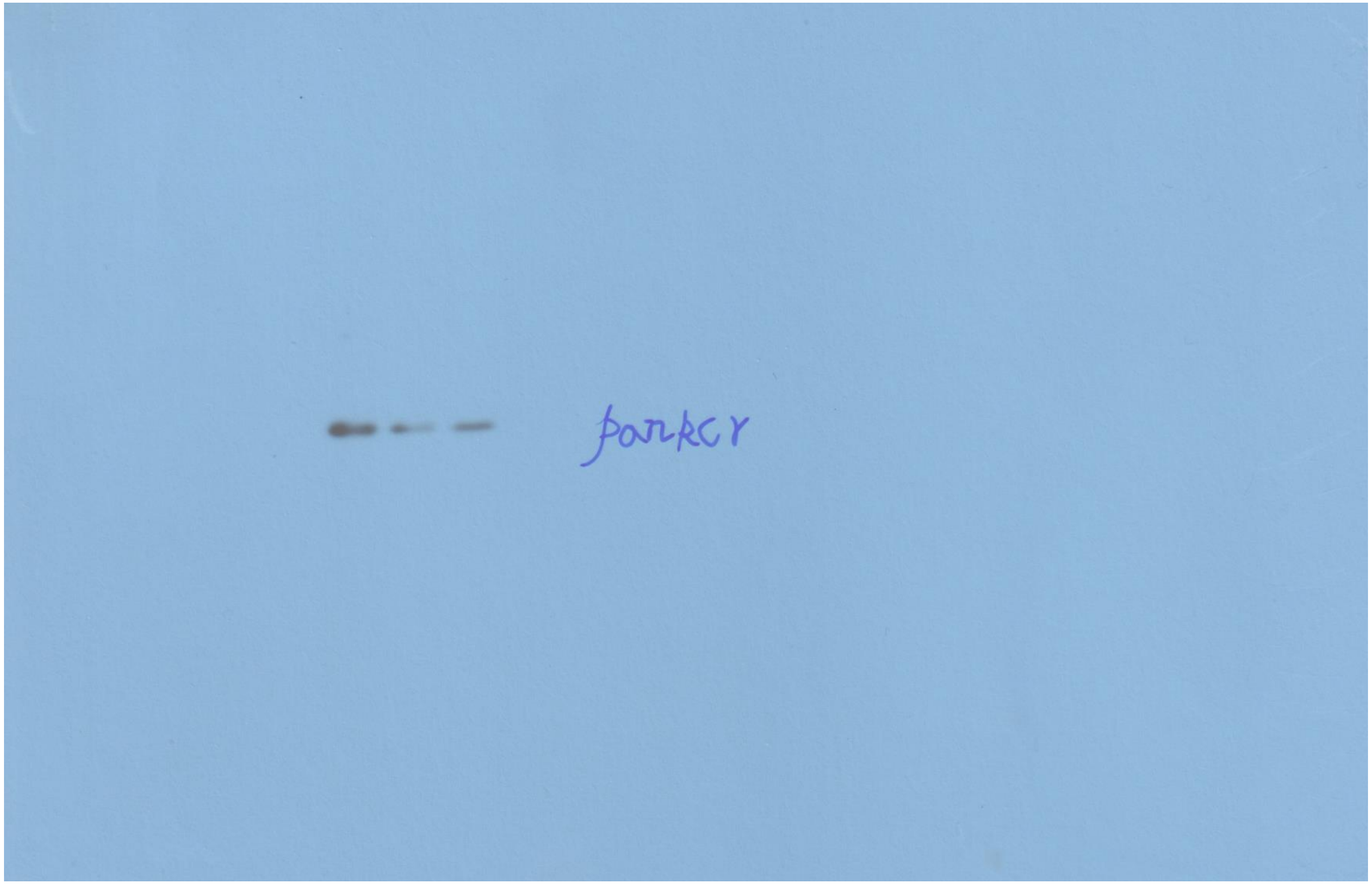

figure 6C—NFL

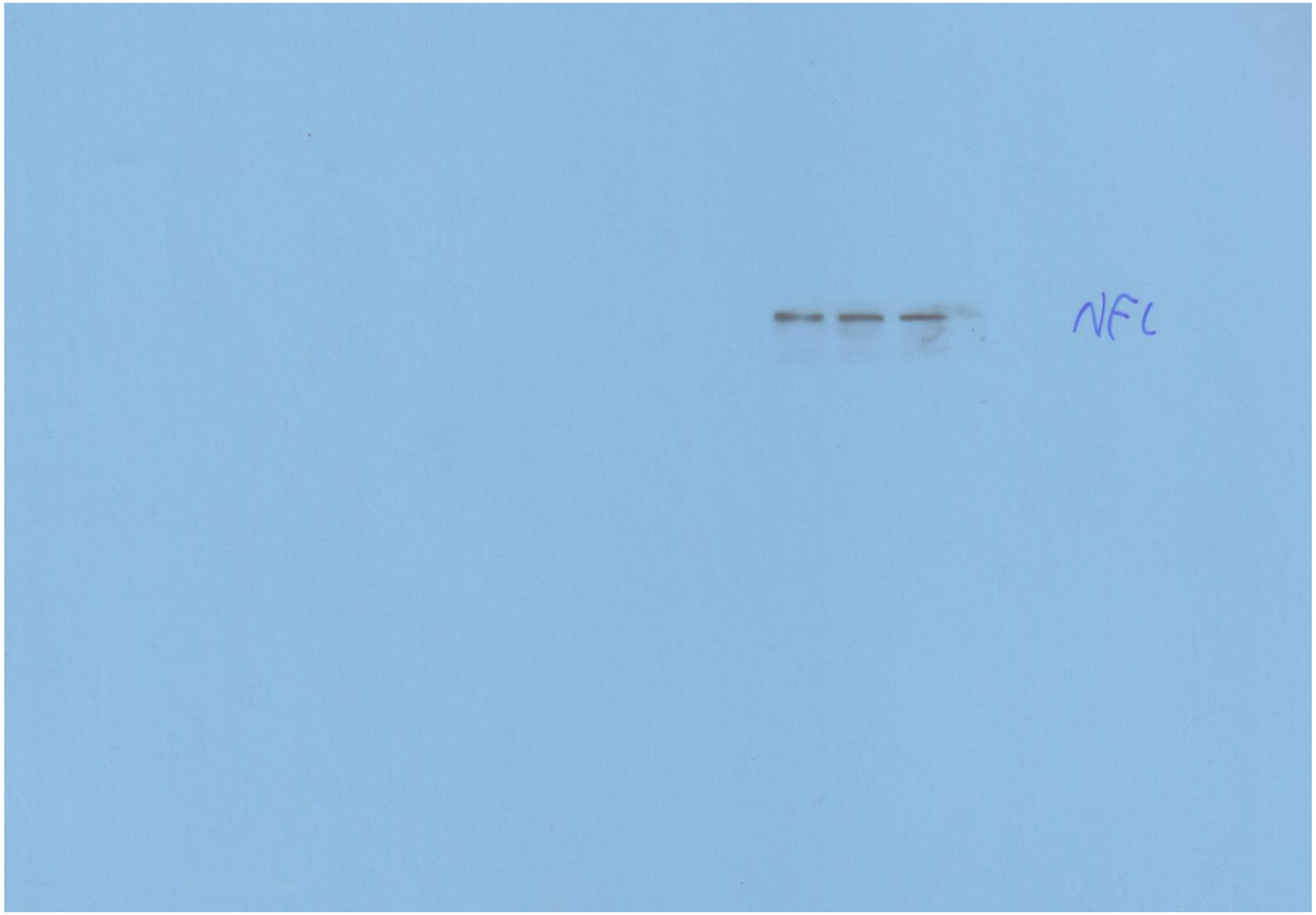

figure 6D—Ubiquitin

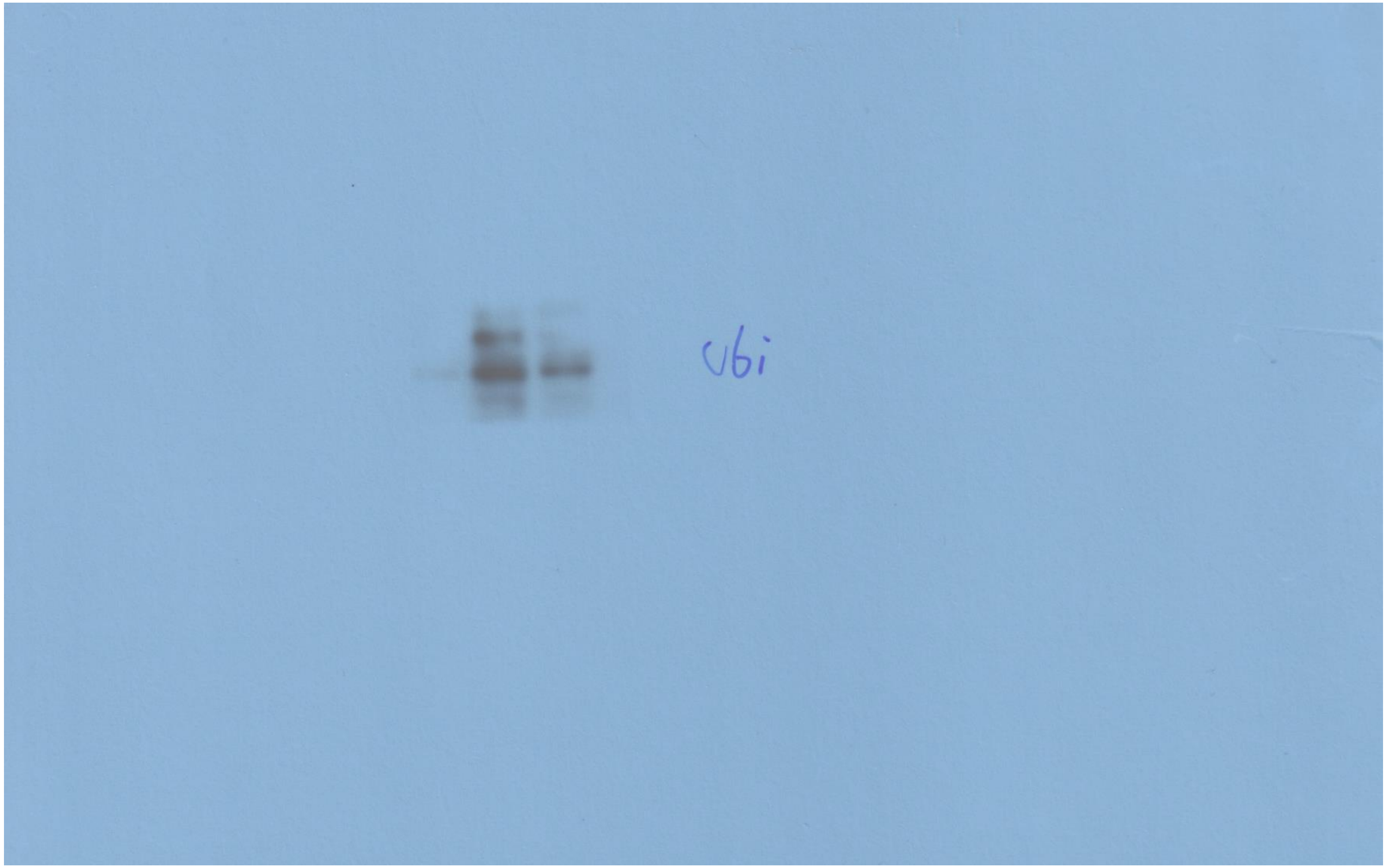

figure 6D—NFL

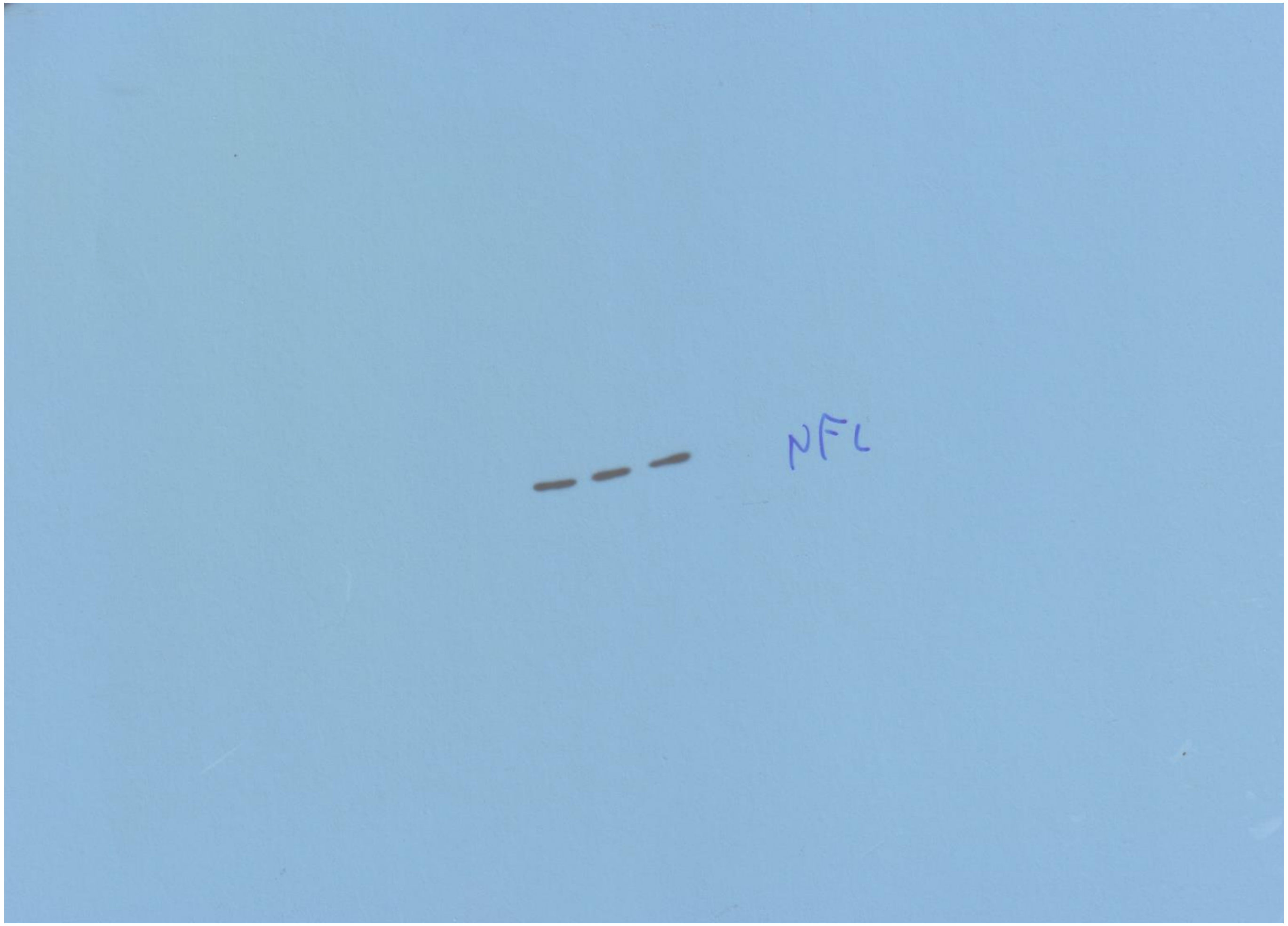

figure 7A—Flag

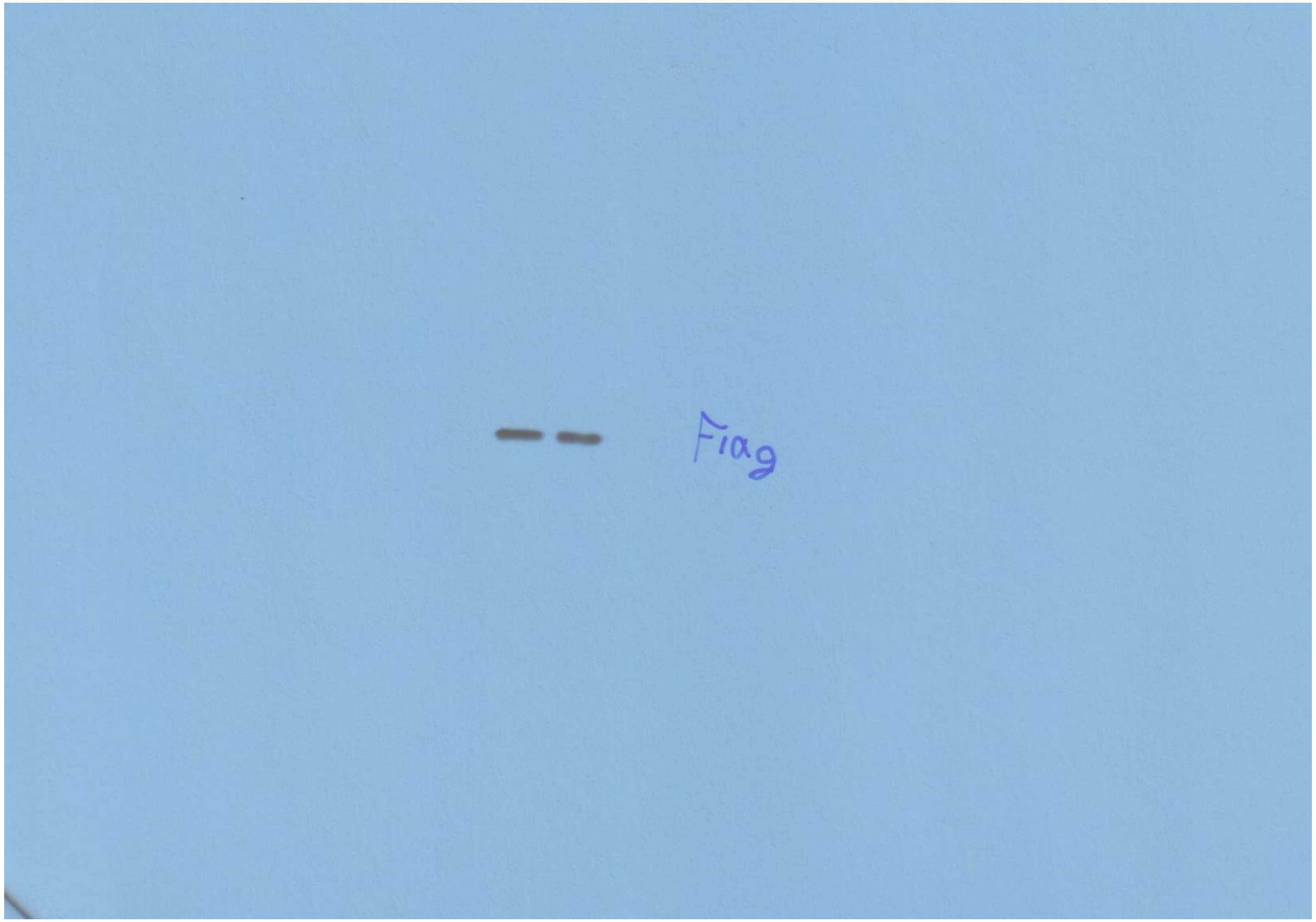

figure 7A—β-actin

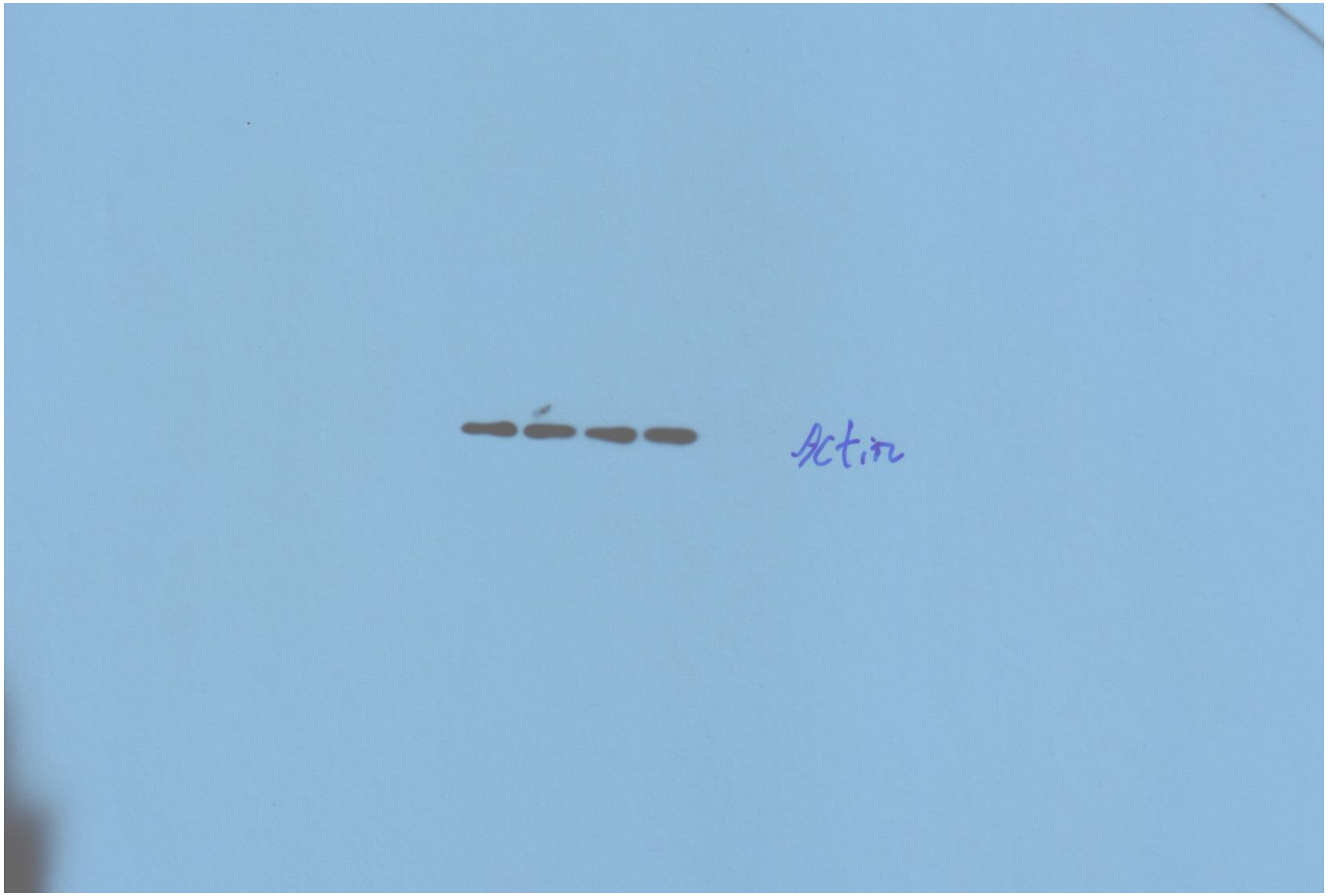

figure 7C—NFL

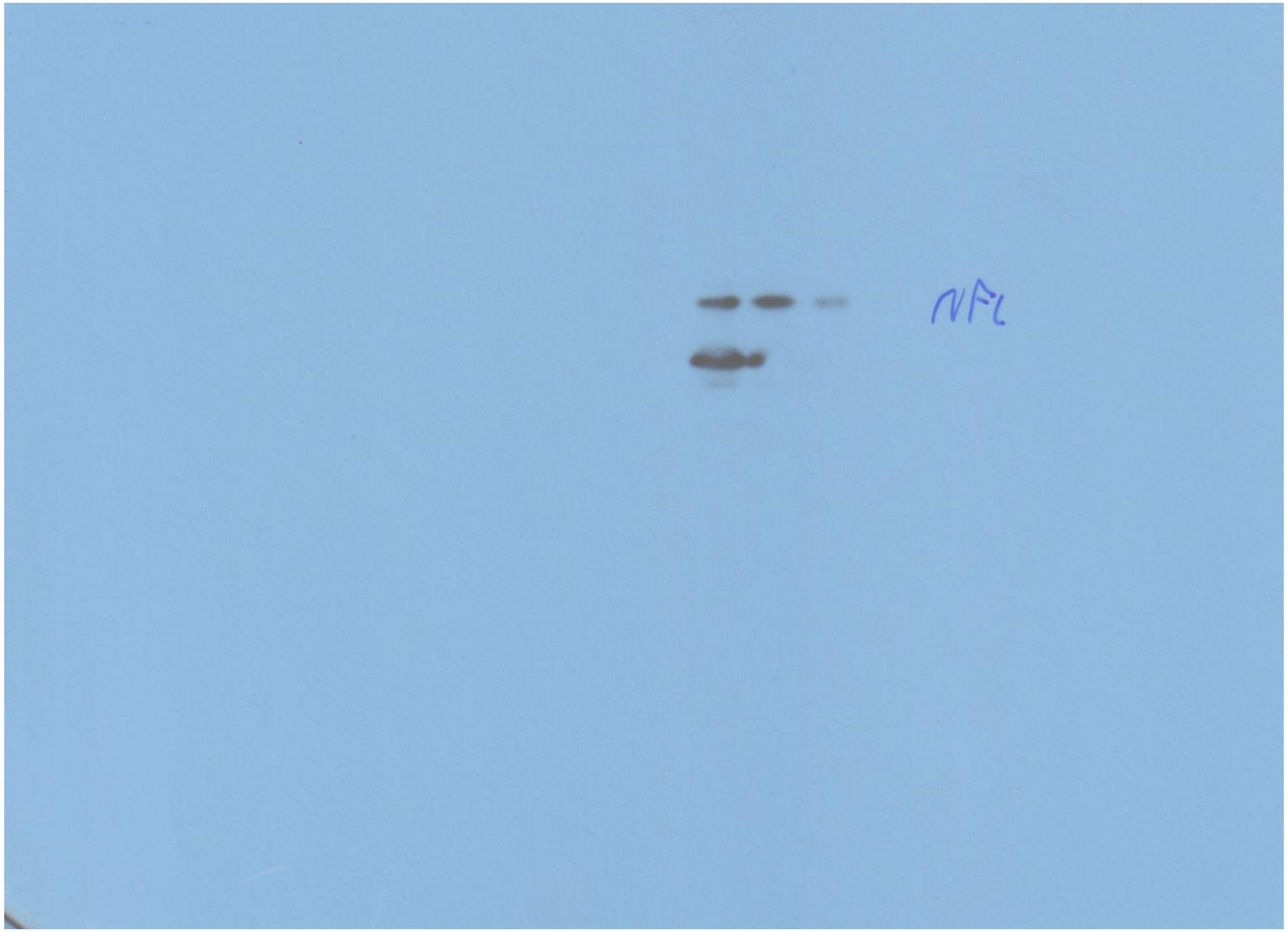

figure 7C—β-actin

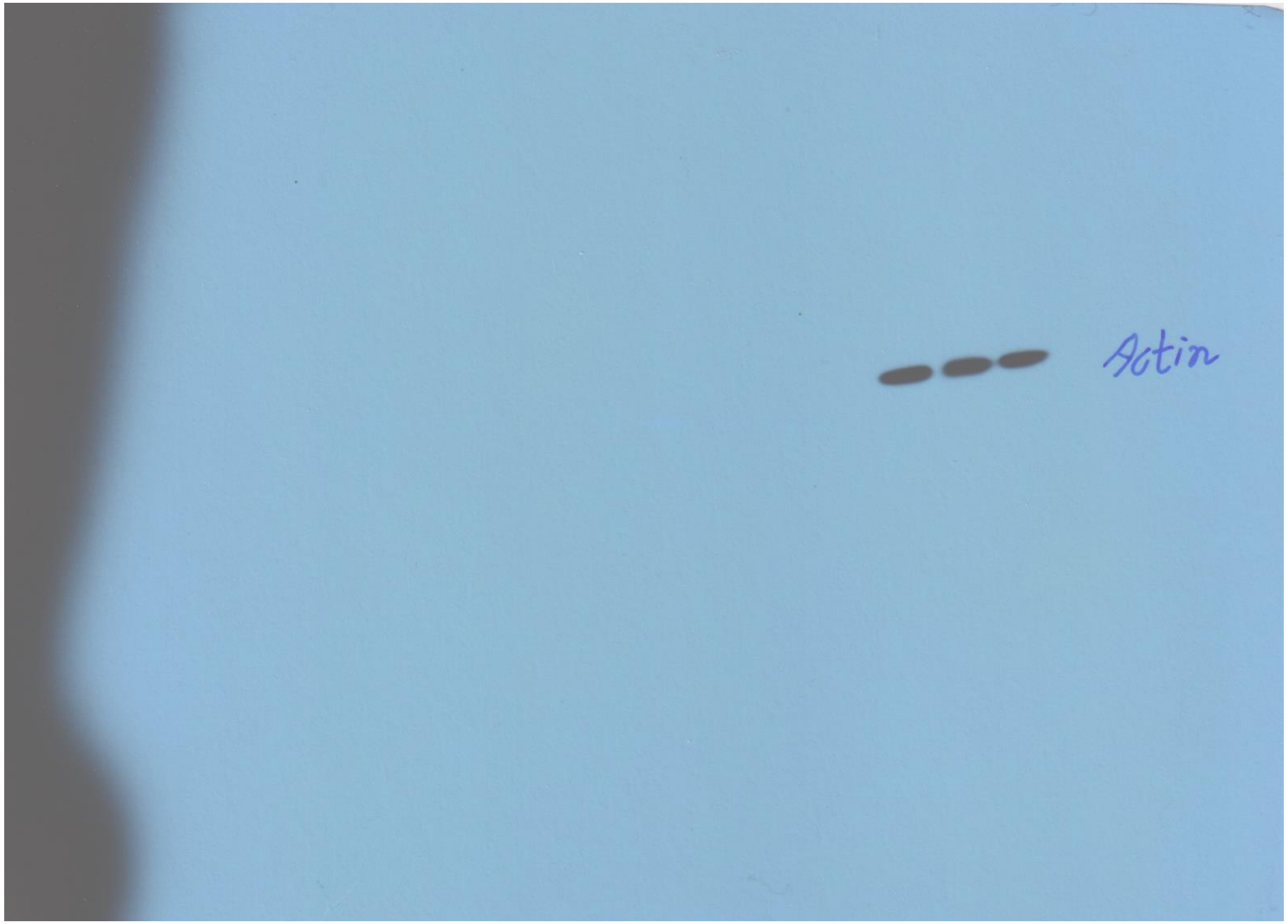

figure 7D—Pankcr

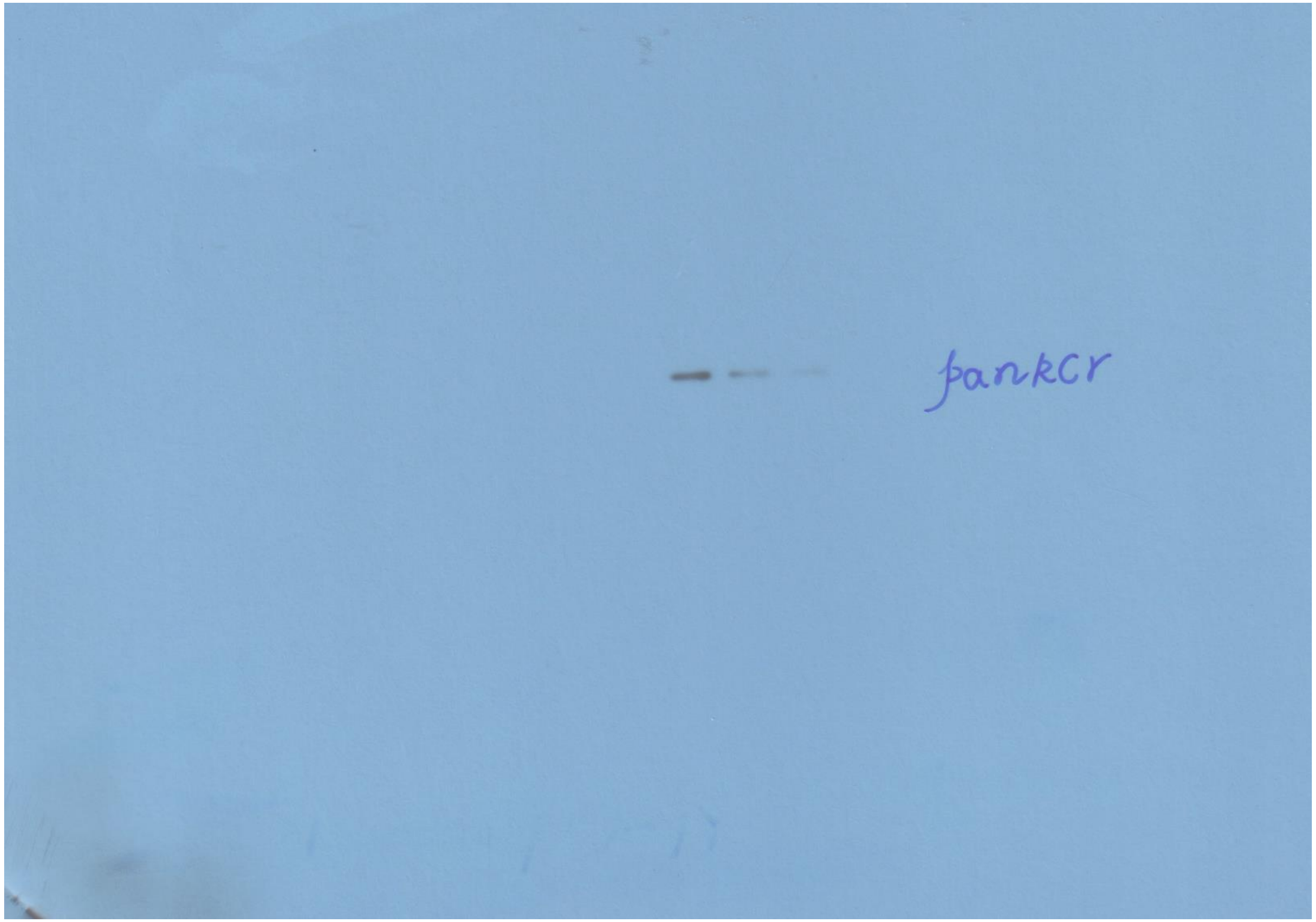

figure 7D—Flag

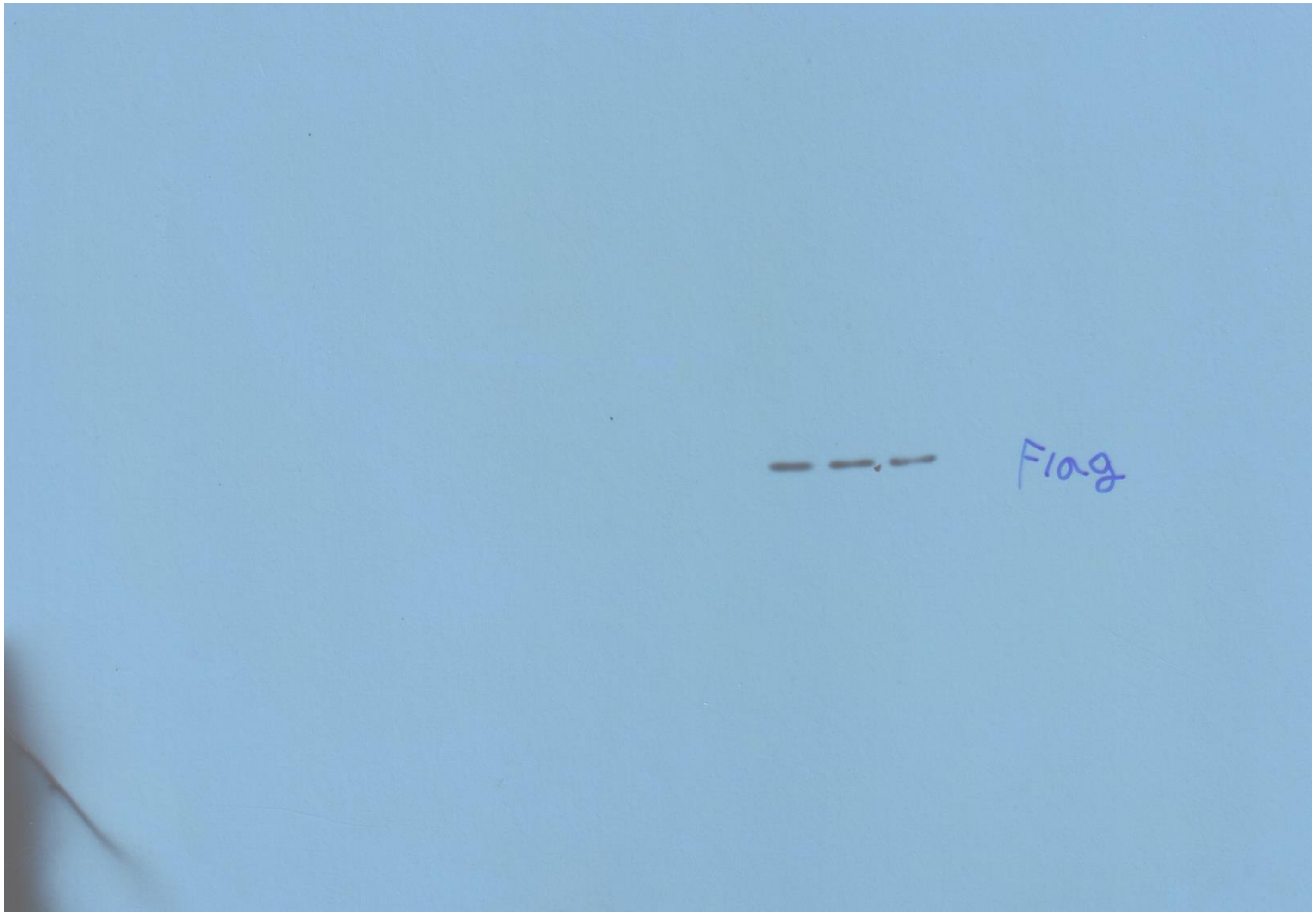

figure 7E—Ubiquitin

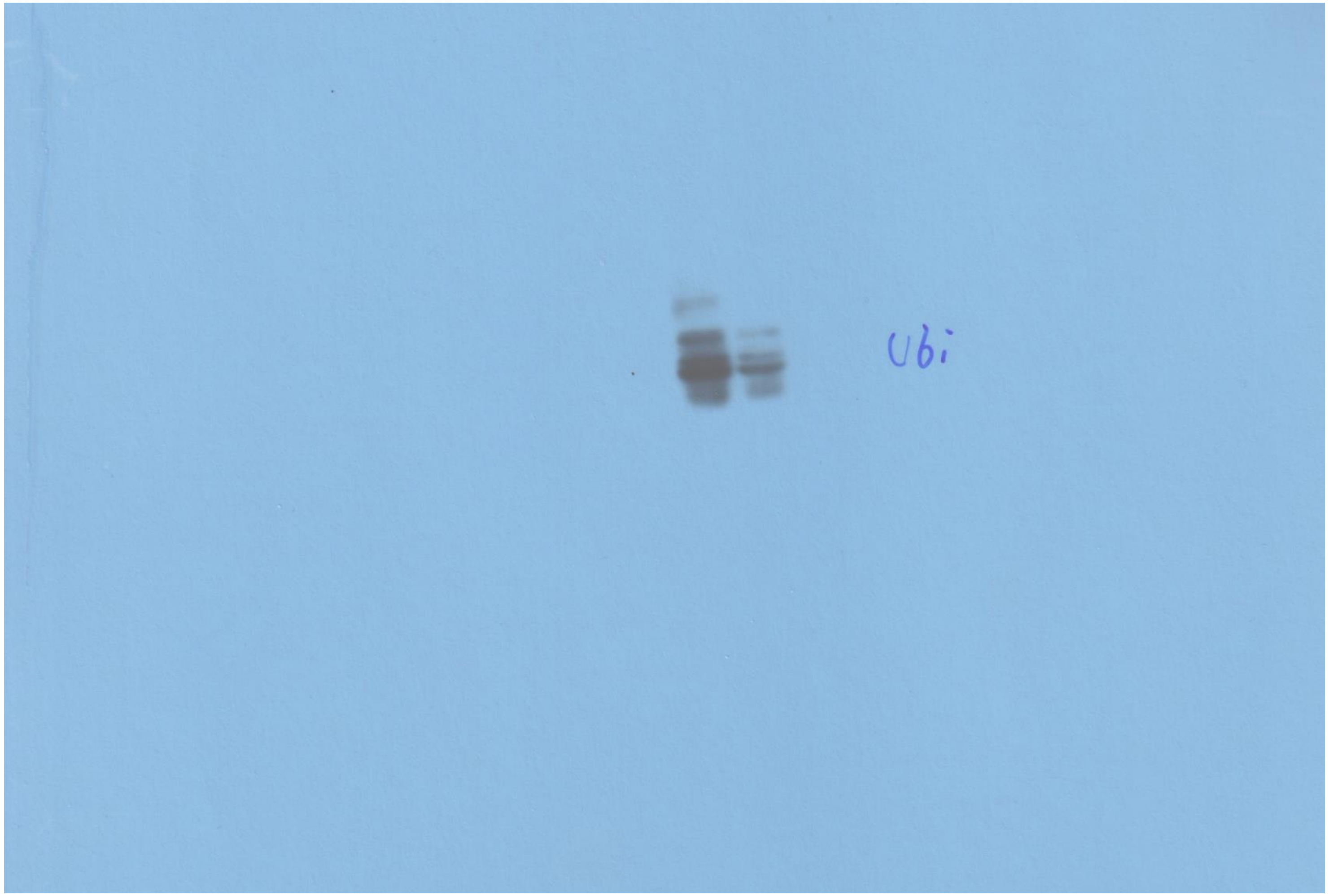

figure 7E—Flag

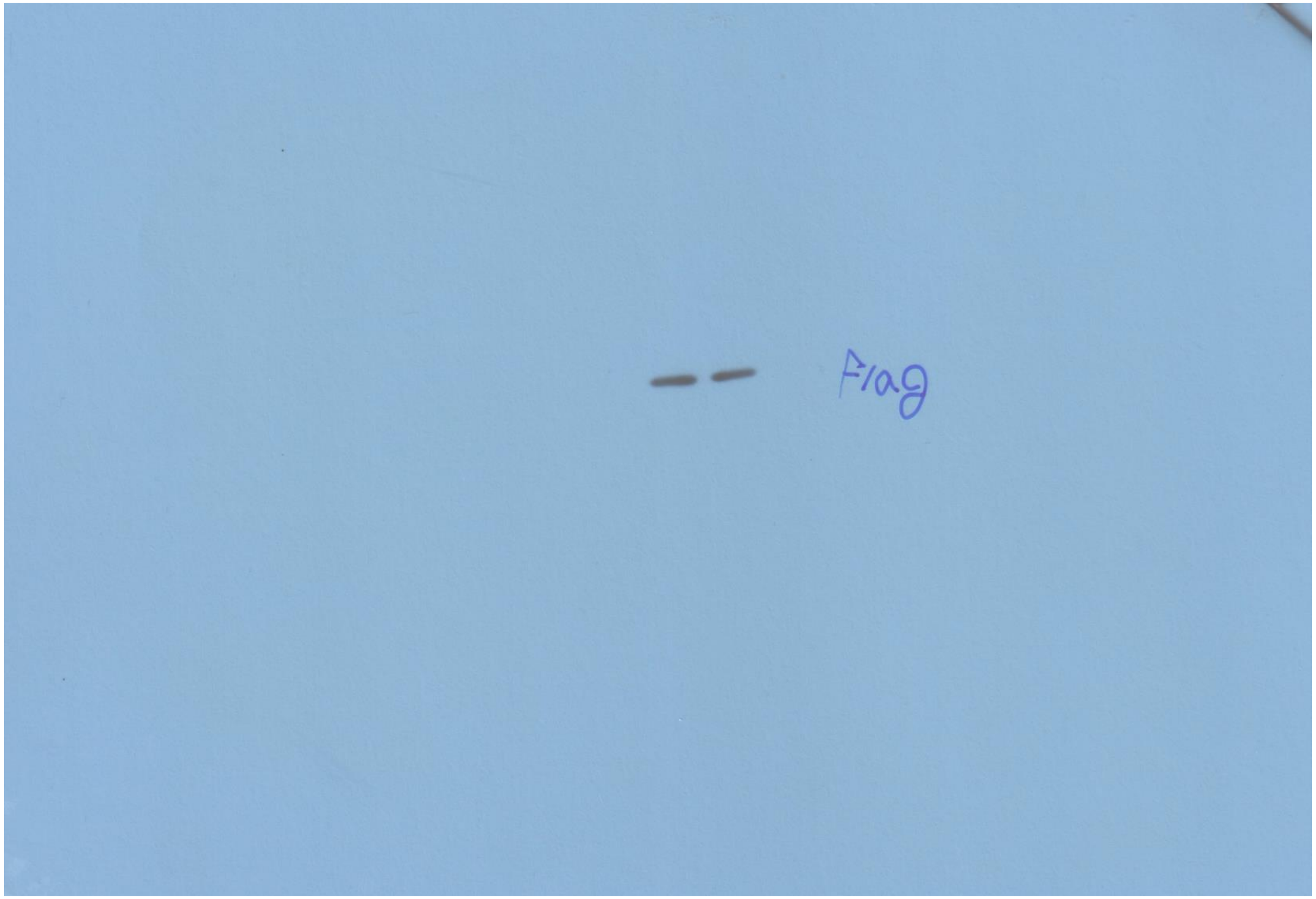

Supplement: Supplementary file 1 — original WB [file 41420_2025_2764_MOESM1_ESM.pdf]
